# Iodine-catalyzed electrophilic substitution of indoles: Synthesis of (un)symmetrical diindolylmethanes with a quaternary carbon center

**DOI:** 10.3762/bjoc.17.102

**Published:** 2021-06-18

**Authors:** Thanigaimalai Pillaiyar, Masoud Sedaghati, Andhika B. Mahardhika, Lukas L. Wendt, Christa E. Müller

**Affiliations:** 1 PharmaCenter Bonn, Pharmaceutical Institute, Pharmaceutical & Medicinal Chemistry, University of Bonn, An der Immenburg 4, D-53121 Bonn, Germany, phone: +49-228-73-2301; Fax: +49-228-73-2567; 2 Pharmaceutical Institute, Pharmaceutical/Medicinal Chemistry, University of Tuebingen, Auf der Morgenstelle 8, 72076 Tuebingen, Germany, phone: +49-7071-29-77458; 3 Research Training Group 1873, University of Bonn, 53127 Bonn, Germany

**Keywords:** alkylation of indole, anti-inflammatory, binding affinity, cannabinoid receptors, diindolylmethane, unsymmetrical 3,3'-diindolylmethane

## Abstract

A novel, versatile approach for the synthesis of unsymmetrical 3,3'-diindolylmethanes (DIMs) with a quaternary carbon center has been developed via iodine-catalyzed coupling of trifluoromethyl(indolyl)phenylmethanols with indoles. In contrast to previously reported methods, the new procedure is characterized by chemoselectivity, mild conditions, high yields, and scalability to obtain gram amounts for biological studies. Selected compounds were found to display affinity for cannabinoid receptors, which are promising drug targets for the treatment of inflammatory and neurodegenerative diseases.

## Introduction

**Figure 1:**
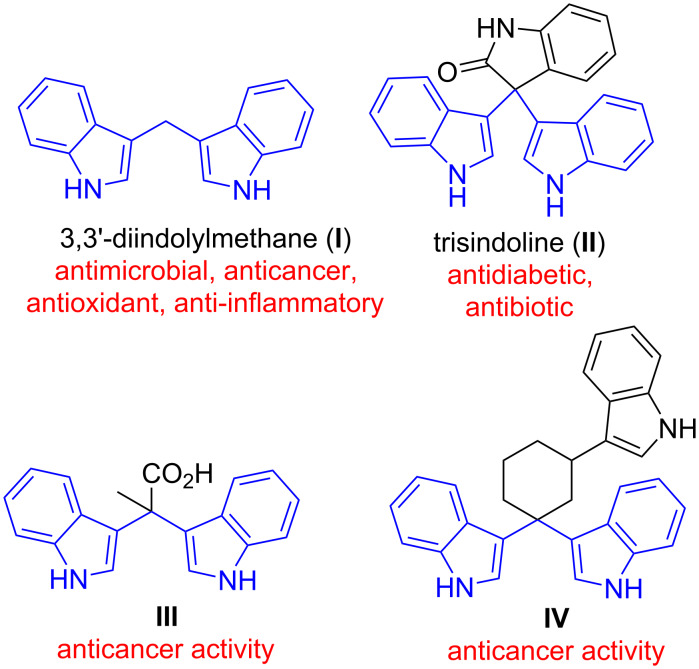
Diindolylmethanes and reported biological activities.

Diindolylmethanes (DIMs) represent an important class of indole alkaloids, that are constituents of pharmaceuticals [1–7] and agrochemicals [8–9]. DIM derivatives possess a variety of biological activities (Figure 1) [10]. Unsubstituted DIM (**I**), for example, exhibits antimicrobial [5], anticancer [11–13], and anti-inflammatory effects (Figure 1) [14]. There is preclinical evidence for activity against several types of cancer [15], and DIM has been clinically evaluated for the treatment of prostate cancer [16] and showed promise for the treatment of cervical dysplasia [17]. The related trisindoline (**II**) was reported to possess antibiotic activity [18], while DIM derivatives **III** and **IV** also showed anticancer activities (Figure 1). Owing to their exciting biological activities, DIM derivatives have recently received increasing attention from synthetic organic chemists, biologists, and pharmacologists.

In general, DIMs can be synthesized via electrophilic substitution of indoles by aldehydes or ketones in the presence of conventional Lewis or Brønsted acids as catalysts [19]. This strategy is straightforward, but it always only provides symmetrical DIMs. The synthesis of unsymmetrical DIM derivatives, however, remains challenging, and merely sporadic examples are reported in literature [20–21].

Methods for the introduction of fluorinated groups into organic molecules are of high interest due to fluorine’s unique physical and chemical properties, such as its small size, high electronegativity, and high C–F bond dissociation energy [22–24]. Organofluoro compounds developed as drug molecules often display increased metabolic stability and bioavailability compared to non-fluorinated analogs [25]. Considering the ever-growing demand for organofluorine compounds, the development of new methodologies that allow the incorporation of fluorine atoms into bioactive molecules is highly desired and will also be addressed herein.

Recently, the use of indol-3-ylmethanols as electrophiles has emerged as a powerful strategy for constructing synthetically valuable indol-3-yl-containing molecules. In particular, the reaction of indol-3-ylmethanols with indoles has become a useful route for the preparation of tertiary unsymmetrical 3,3'-DIMs [26–35]. However, synthetic methods for efficient synthesis of unsymmetrical 3,3'-DIMs with a quaternary carbon center, including trifluoromethyl-substituted 3,3'-DIMs, are still rare.

**Figure 2:**
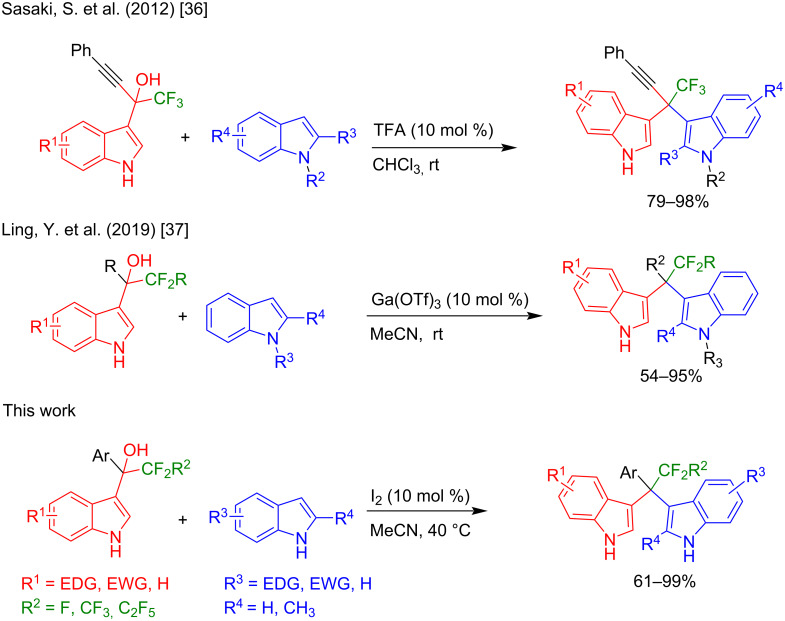
Synthetic strategies toward trifluoromethylated unsymmetrical quaternary DIMs.

Sasaki et al. reported the reaction of trifluoromethyl(indolyl)phenylmethanols with indoles in the presence of trifluoroacetic acid (TFA) and CHCl_3_ (Figure 2) [36]. Very recently, Ling et al. reported the same reaction in the presence of Ga(OTf)_3_ in acetonitrile (Figure 2) [37]. Although these methods are certainly useful, they have several undeniable drawbacks, including the use of heavy-metal catalysts and the necessity of employing indoles bearing bulky substituents at their 2-position (Ling et al.), or the need for chlorinated solvents (Sasaki et al.), as well as difficulty to scale up the reactions to a multigram scale, as well as a generally rather limited substrate scope. Therefore, finding a robust method with a broad substrate scope and functional group tolerance is highly desirable.

As part of our continuous efforts to prepare biologically active DIM derivatives [38], we herein report an innovative approach to synthesize unsymmetrical 3,3'-diindolylmethanes (DIMs) with a fluoromethyl-containing quaternary carbon center via an iodine-catalyzed coupling reaction of trifluoromethyl(indolyl)phenylmethanol with indole derivatives. This method has also been extended to the synthesis of pentafluoro-ethylated and heptafluoro-propylated DIMs in excellent yields. Selected compounds were evaluated in radioligand binding studies for their affinities towards cannabinoid CB_1_ and CB_2_ receptors.

## Results and Discussion

### Optimization of the reaction

The reaction conditions were optimized using 2,2,2-trifluoro-1-(5-methoxy-1*H*-indol-3-yl)-1-phenylethan-1-ol (**1a**, 5 mmol) and 1*H*-indole (**2a**, 5 mmol) as model substrates (Table 1). At first, the reaction was attempted in trifluoroethanol (TFE), and water, respectively, as these solvents had been utilized for the preparation of unsymmetrical DIMs from (1*H*-indol-3-yl)(phenyl)methanol by Xiao and co-workers [39–40]. However, no product was formed in either solvent even at high temperatures (Table 1, entries 1–3). This is likely due to the steric hindrance of the CF_3_-substituted quaternary carbon atom in substrate **1a**. Therefore, the solvent was changed to H_2_SO_4_ (5%) in water (Table 1, entry 4) or glacial acetic acid (entry 5), and the reactions were performed at room temperature. While no reaction occurred in 5% H_2_SO_4_, traces of product were observed in acetic acid (entry 5). Therefore, the reaction mixture was gradually heated to 50 °C (Table 1, entry 6), 80 °C (entry 7), and 100 °C (entry 8). To our delight, the formation of the expected product was steadily increased to 32, 47, and 56%, respectively. Nevertheless, it was not possible to further increase the yield of the product using this solvent.

**Table 1:** Optimization of the reaction conditions for the preparation of 5-methoxy-3-(2,2,2-trifluoro-1-(1*H*-indol-3-yl)-1-phenylethyl)-1*H*-indole (**3a**)^a^.

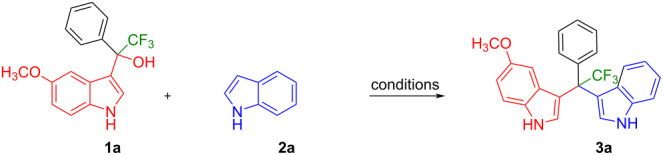					

Entry	Solvent	Catalyst	Temp. (°C)	Time (h)	Yield (%)^b^

1	trifluoroethanol	–	rt	24	0^c^
2	trifluoroethanol	–	80	24	0^c^
3	H_2_O	–	100	24	0^c^
4	5% H_2_SO_4_ in H_2_O	–	rt	24	0^c^
5	CH_3_COOH	–	rt	24	traces
6	CH_3_COOH	–	50	24	32
7	CH_3_COOH	–	80	24	47
8	CH_3_COOH	–	100	24	56
9	MeCN	AlCl_3_ (10 mol %)	rt	24	10
10	MeCN	FeCl_3_ (10 mol %)	rt	24	17
11	MeCN	I_2_ (10 mol %)	rt	24	51
12	MeCN	*p*-TsOH (10 mol %)	rt	24	5
13	MeCN	InCl_3_ (10 mol %)	rt	12	traces
14	MeCN	FeCl_3_ (10 mol %)	40	12	67
15	MeCN	*p*-TsOH (10 mol %)	40	24	15
**16**	**MeCN**	**I_2 _(10 mol %)**	**40**	**5**	**98**
17	MeCN	I_2_ (10 mol %)	80	5	95
18	MeCN	I_2_ (5 mol %)	40	12	89

^a^Reactions of **1a** (5 mmol) and **2a** (5 mmol) were performed in 5 mL of solvent. ^b^Isolated yields after column chromatography. ^c^No reaction. MeCN, acetonitrile. rt, room temperature.

For subsequent attempts, we investigated the reaction using different Lewis acid catalysts, including AlCl_3_ (Table 1, entry 9, 10% yield), FeCl_3_ (entry 10, 17%), I_2_ (entry 11, 51%), *p*-TsOH (entry 12, 5%), and InCl_3_ (entry 13, traces), in acetonitrile at room temperature. Among these, the presence of I_2_ led to the highest yield of 51% (Table 1, entry 11). This trend was consistent: Upon heating to 40 °C, reactions with FeCl_3_ (Table 1, entry 14) or *p*-TsOH (entry 15) yielded 67% and 15% of product, respectively, while 98% of the product was obtained in the presence of I_2_ (Table 1, entry 16). However, further increase of the reaction temperature to 80 °C did not significantly affect the generation of the product (Table 1, entry 17). Lowering the amount of catalyst from 10 mol % to 5 mol % reduced the product formation (Table 1, entry 18).

Having optimized the reaction conditions (I_2,_ 10 mol %, 40 °C for 5 h in MeCN; entry 16, Table 1), we explored the scope of the reaction. At first, we employed differently substituted indole derivatives (Table 2). A large variety of substituted indoles was well tolerated, and their reactions with **1a** provided the desired products in good to excellent yields (61–99%). Reaction of **1a** with indoles bearing electron-donating substituents, such as methoxy (**2b**) or hydroxy (**2f**), afforded the products in good yields (**3b**: 67%; **3f**: 61%). Coupling of **1a** with indoles substituted with electron-withdrawing groups, including cyano (**2c**), fluoro (**2d**, **2e**, **2h**), and bromo (**2g**), likewise resulted in good to excellent yields of the desired products (**3c**: 65%, **3d**: 99%, **3e**: 96%, **3g**: 95%, **3h**: 91%).

**Table 2:** Substrate scope of the reaction with differently substitute indole derivatives **2**.

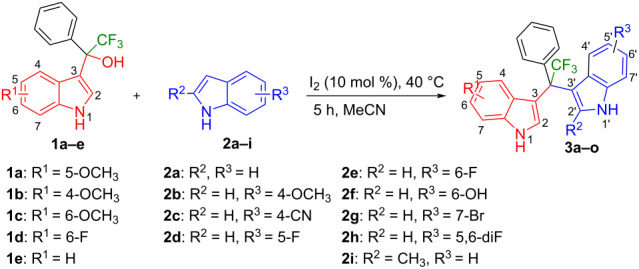		

Yields of products **3a–o**		

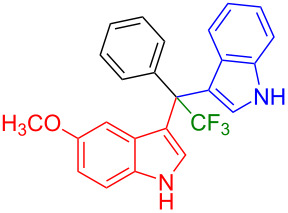 **3a** (98%) (81%)[35]	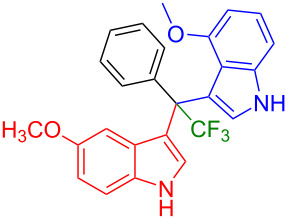 **3b** (67%)	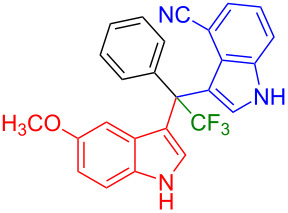 **3c** (65%)
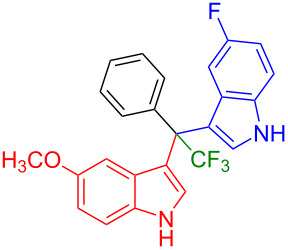 **3d** (99%), (80%)[35]	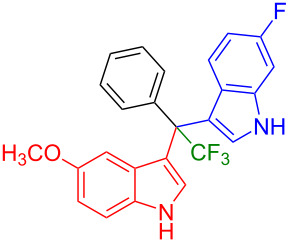 **3e** (96%)	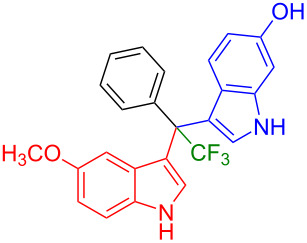 **3f** (61%)
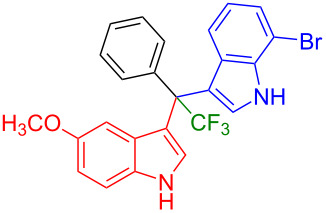 **3g** (95%)	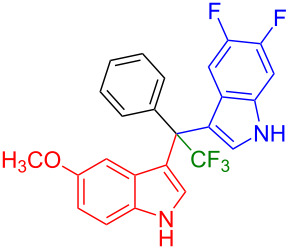 **3h** (91%)	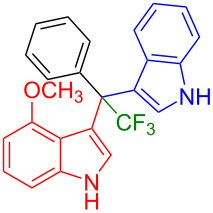 **3i** (77%)
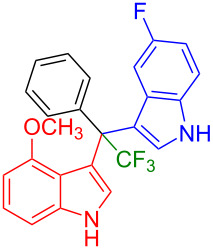 **3j** (89%)	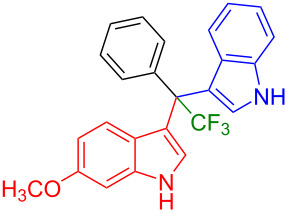 **3k** (70%)	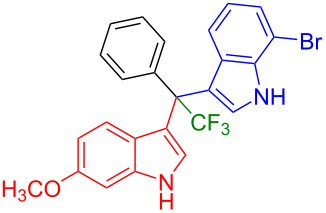 **3l** (72%)
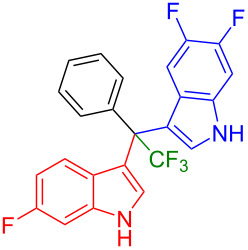 **3m** (89%)	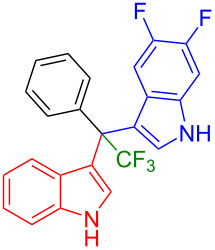 **3n** (99%)	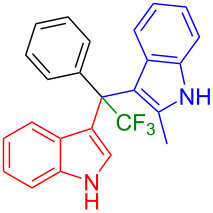 **3o** (87%)

Shifting the position of the methoxy group of **1a** from position 5 to 4 (**1b**) or 6 (**1c**) also led to the formation of the products in very good yields (**3i**: 77%, **3j**: 89%, **3k**: 70%, **3l**: 72%). The intermediates **1d** with 6-fluoro or **1e** without substituent on the indole ring reacted with 5,6-difluoroindole (**2h**) and formed the desired products in excellent yields of 89% (**3m**) and 99% (**3n**). Besides, 2-methylindole (**2i**) smoothly reacted with **1e**, affording product **3o** in 87% yield.

Next, we studied the substrate scope of the trifluoromethyl(indolyl)phenylmethanols **1** with respect to the substitution of the phenyl ring (Table 3). The derivatives bearing *p*-tolyl (**1f**), *p*-fluorophenyl (**1g**), or *p*-bromophenyl (**1g**) rings reacted with a series of halogenated indoles (**2d**, **2e**, **2f**, **2g**, **2h**, and **2j**) providing the unsymmetrical DIMs (**3p**: 99%, **3q**: 98%, **3r**: 87%, **3s**: 90%, **3t**: 82%, **3u**: 92%) in excellent yields ranging from 82–99%. It was interesting to see that a compound, in which the phenyl ring of **1** was replaced by the heteroaryl moiety thiophene (**1i**), reacted efficiently with a series of indole derivatives (**2a**, **2d**, **2h**) providing yields of 82–90% (**3v**–**x**).

**Table 3:** Substrate scope of the reaction of **1f**–**i** with trifluoromethyl(indolyl)phenylmethanols **1**: modification of the aryl group.

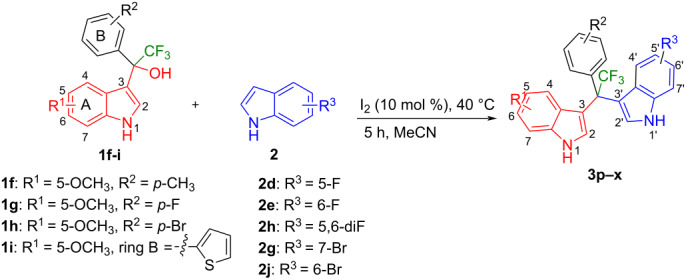		

Yields of products **3p–x**		

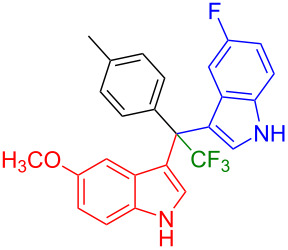 **3p** (99%)	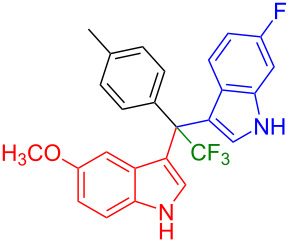 **3q** (98%)	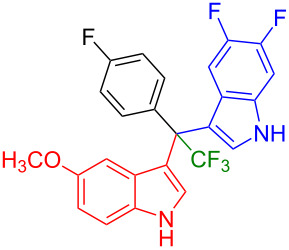 **3r** (87%)
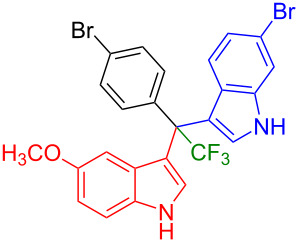 **3s** (90%)	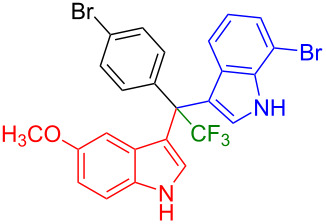 **3t** (82%)	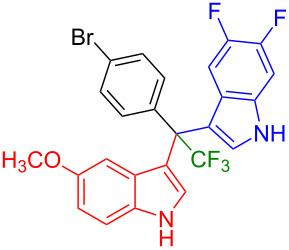 **3u** (92%)
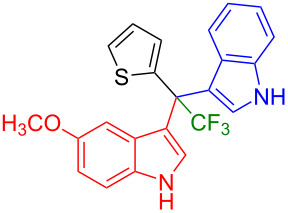 **3v** (89%)	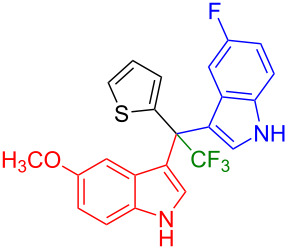 **3w** (90%)	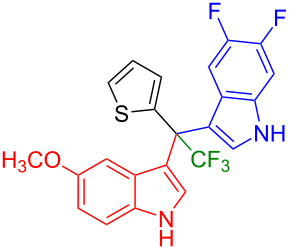 **3x** (82%)

We further extended this protocol to the preparation of unsymmetrical pentafluoroethylated and heptafluoropropylated DIM derivatives (Table 4). The (indol-3-yl)phenylmethanol derivative bearing a pentafluoroethyl residue (**1j**) was efficiently reacted with a series of indole derivatives (**2d**, **2e**, **2h**, **2j**, and **2k**) substituted either both electron-donating or electron-withdrawing groups, and provided the desired products (**3y**: 96%, **3z**: 89%, **3aa**: 90%, **3ab**: 86%, **3ac**: 92%, **3ad**: 94%) in excellent yields (86–96%).

**Table 4:** Substrate scope of the reaction of **1j**–**l** with trifluoromethyl(indolyl)phenylmethanols **1**: modification of the trifluoromethyl group.

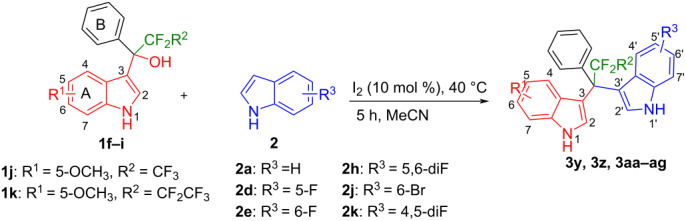		

Yields of products **3y,z,aa–ag**		

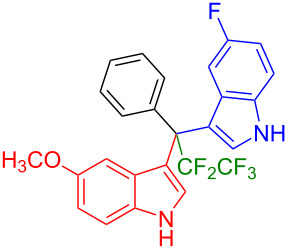 **3y** (96%)	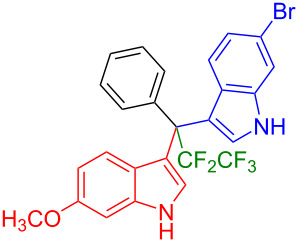 **3z** (89%)	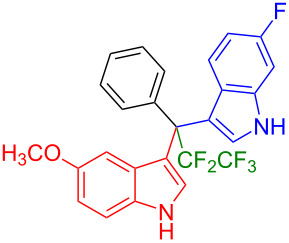 **3aa** (90%)
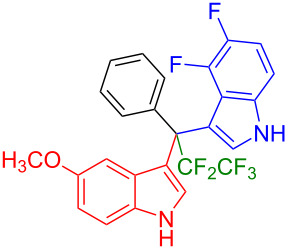 **3ab** (86%)	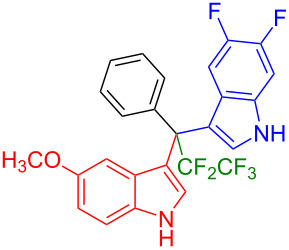 **3ac** (92%)	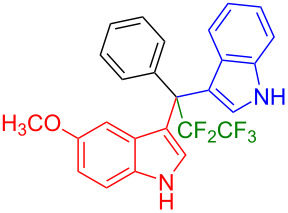 **3ad** (94%)
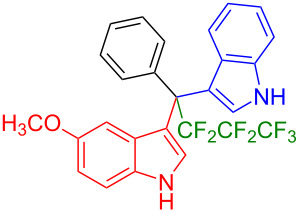 **3ae** (65%)	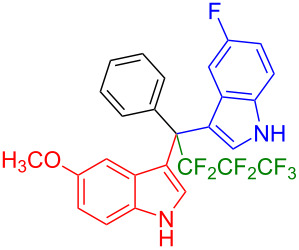 **3af** (72%)	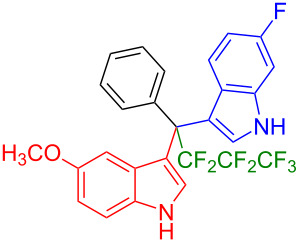 **3ag** (70%)

The (indol-3-yl)phenylmethanol derivative bearing a heptafluoropropyl residue (**1k**) underwent coupling reactions with indole derivatives (**2a**, **2d**, and **2e**) and yielded the expected products (**3ae**: 65%, **3af**: 72%, **3ag**: 70%) in very good yields (65–72%). It was observed that the yield of heptafluoropropylated DIMs was slightly lower compared to their pentafluoroethylated congeners (compare **3y**: 96% vs **3af**: 72%, and **3aa**: 90% vs **3ag** 70%).

**Figure 3:**
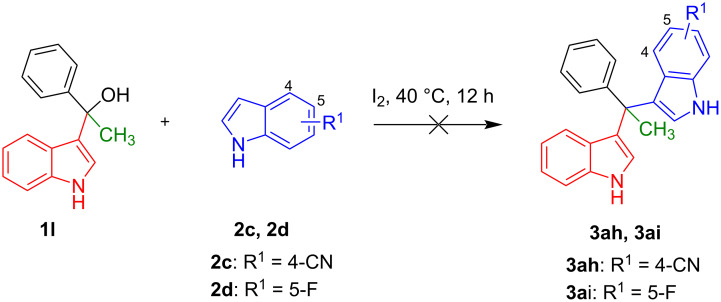
Reactions performed to study the scope of the method.

Next, the necessity of the fluoroalkyl substituent was investigated to study the scope of the reaction. The non-fluorinated (indol-3-yl)phenylmethanol derivative bearing a methyl residue (**1l)** and **2c** or **2d** were reacted applying the optimized conditions. However, the desired products **3ah** and **3ai** were not produced (Figure 3). These experiments indicated that the presence of a fluoroalkyl substituent was indeed essential for the alkylation of indoles.

**Figure 4:**
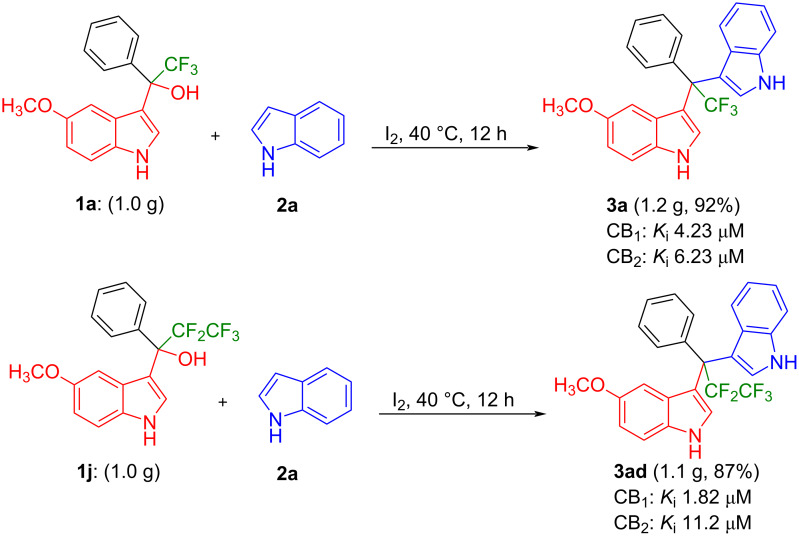
Gram-scale synthesis of unsymmetrical DIMs **3a** and **3ad**.

We further investigated the new method’s feasibility for large-scale synthesis (Figure 4). Thus, 2,2,2-trifluoro-1-(5-methoxy-1*H*-indol-3-yl)-1-phenylethan-1-ol (**1a**, 1.0 g, 3.10 mmol) and 2,2,3,3,3-pentafluoro-1-(5-methoxy-1*H*-indol-3-yl)-1-phenylpropan-1-ol (**1j**, 1.0 g, 2.7 mmol) were reacted with indole (**2a**, 0.40 g, 3.4 mmol; and 0.347 g, 2.9 mmol, respectively). The reactions proceeded without significant loss in efficiency, affording 1.2 g of **3a** (92% yield) and 1.10 g of **3ad** (87% yield).

The unsubstituted diindolylmethane (**I**, Figure 1) was previously reported to bind to the cannabinoid receptors, CB_1_ (*K*_i_ 4.3 µM) and CB_2_ (*K*_i_ 1.1 µM) [41]. Both receptors are considered important therapeutic targets, e.g. for neurodegenerative and inflammatory diseases. Selected final products (**3a**, **3b, 3e**, **3g**, **3h**, **3n**, **3ad**) were tested for their binding affinities towards human CB_1_ and CB_2_ receptors (Table 5).

**Table 5:** Binding affinities of unsymmetrical fluoromethyl-substituted DIM derivatives for cannabinoid receptors.

Compound	Structure	Human CB_1_ receptor	Human CB_2_ receptor

		Radioligand binding assay	

		*K*_i_ ± SEM (µM) (vs [^3^H]CP55,940)	*K*_i_ ± SEM (µM) (vs [^3^H]CP55,940)

**I** [41]	See Figure 1 for structure	4.3	1.1
**3a**	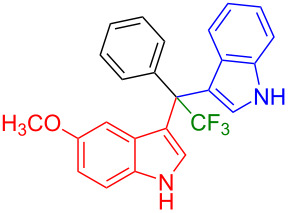	4.23 ± 0.03	6.04 ± 0.11
**3b**	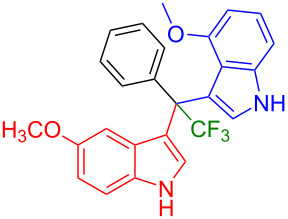	7.64 ± 0.80	4.75 ± 0.34
**3e**	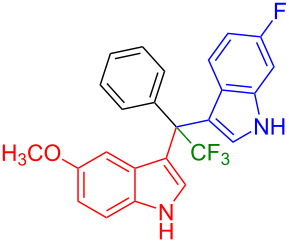	3.21 ± 0.25	4.47 ± 0.12
**3g**	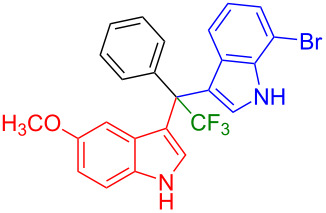	4.02 ± 0.22	4.62 ± 0.33
**3h**	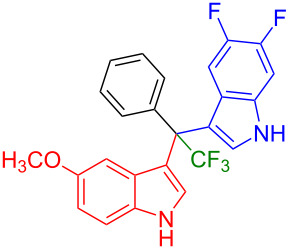	4.78 ± 1.26	9.90 ± 1.45
**3n**	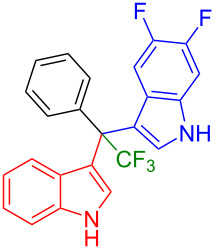	2.04 ± 0.08	6.14 ± 0.13
**3ad**	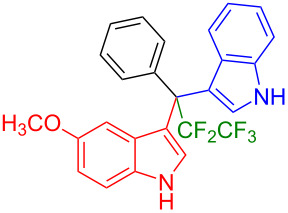	1.82 ± 0.09	11.2 ± 0.5

At the CB_1_ receptor, compound **3a** with a methoxy substituent on one of the two indole rings showed equipotent affinity to lead compound **I**, while introducing an additional 4-methoxy moiety into the second indole ring reduced binding affinity (**3b**). Compounds bearing 5-OMe,6'-F (**3e**), 5-OMe,7'-Br (**3g**), and 5-OMe,5',6'-diF substitution (**3h**) exhibited similar binding affinities to lead compound **I**. 5,6-DiF-DIM derivative **3n** (CB_1_: *K*_i_ 2.04 µM) showed a slightly improved binding affinity compared to lead compound **I**. These results suggest that compounds with small substituents like fluoro on only one indole ring are favorable for CB receptor binding. The pentafluoroethylated DIM derivative **3ad** was the best CB_1_ ligand of the present series with a *K*_i_ value of 1.82 µM. The binding curves of **3e** and **3ad** are depicted in Supporting Information File 1, Figure S1.

Compound **3e** showed similar binding affinities at both CB_1_ and CB_2_ receptor. Therefore, it was selected to determine and compare its functional activity at both receptor subtypes. Compound **3ad** was selected due to its high CB_2_ receptor affinity and selectivity. It is well known that CB_1_ receptors exhibit high constitutive activity [41]. Compound **3e** reduced the basal activity of CB_1_ receptors (Supporting Information File 1, Figure S2A) but not that of CB_2_ receptors indicating that this compound acts as an inverse agonist (EC_50_ 0.786 ± 0.233 µM) at CB_1_ receptors (Supporting Information File 1, Figure S2B). This effect was less pronounced for **3ad**. Non-transfected cells used as controls also did not show any effect after treatment with **3ad** (Supporting Information File 1, Figure S2C). DIM was previously shown to be weak inverse agonist at CB_1_ receptors which is consistent with our current findings for DIM derivatives **3ad** and especially **3e** [42]. Next, we investigated the antagonistic effect of **3e** at CB_1_ receptors (Supporting Information File 1, Figure S3A). Compound **3e** blocked CB_1_ receptor activation with an IC_50_ value of 5.68 ± 0.54 µM, while it was weaker in inhibiting CB_2_ receptor activation. Similarly, **3ad** was also able to fully block CB_1_ receptor activation (IC_50_ value of 5.22 ± 0.68 µM). Our results indicate that the new DIM derivatives act as potent CB_1_ receptor antagonists with inverse agonistic activity, i.e., they stabilize the inactive receptor conformation. Further optimization is warranted. This class of compounds also possesses potential for the development of CB_2_-selective or dual CB_1_/CB_2_-receptor antagonists.

**Figure 5:**
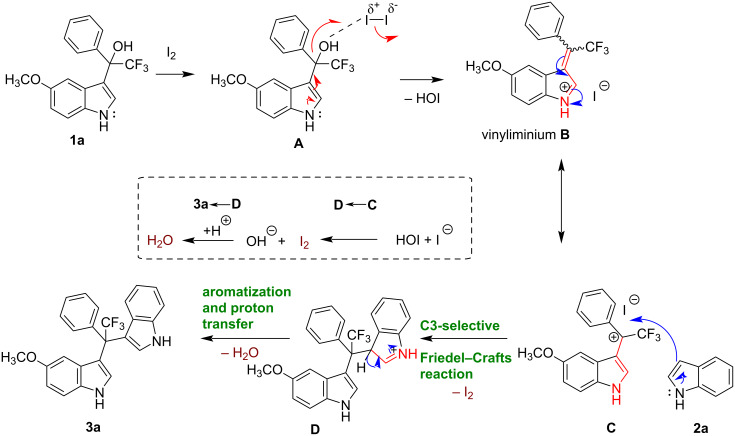
Plausible reaction mechanism for the synthesis of fluoromethylated unsymmetrical DIMs, shown for compound **3a** as an example.

Based on previous reports [25–35,43–53], a plausible reaction mechanism is proposed for the synthesis of **3a** as an example, as depicted in Figure 5. We suggest that the reaction is initiated by iodine-mediated activation of the secondary alcohol in compound **1a** (**A**), followed by elimination of HOI to generate the vinyliminium ion species **B** (see mesomeric structure **C**) [52]. This electrophilic intermediate undergoes a C3-selective Friedel−Crafts reaction with **2a** to deliver intermediate **D**, and the catalyst I_2_ is regenerated by the reaction of HOI and I^−^ (see **C** to **D** in box highlighted by dashed line). The intermediate **D** is stabilized by aromatization yielding product **3a** and H_2_O.

## Conclusion

The described novel and efficient synthetic protocol provides a convenient access to a wide range of unsymmetrical trifluoromethylated 3,3'-diindolylmethanes via I_2_-catalyzed Friedel–Crafts alkylation reaction of trifluoromethylated (indol-3-yl)-1-phenylethan-1-ols with substituted indoles. The method was also extended to the synthesis of pentafluoroethylated and heptafluoropropylated-DIMs. It constitutes an important addition to the active field of DIM syntheses facilitating the preparation of unsymmetrical quaternary DIMs without the need for chlorinated solvents, high temperatures, or heavy-metal catalysts. A broad range of substrates is tolerated and the reaction is suitable for large-scale preparation of the target compounds. The outlined methodology allows for the rapid generation of structurally diverse DIM derivatives to study structure–activity relationships, to optimize biological activity and other properties in order to prepare tool compounds and future drugs. Several compounds displayed micromolar binding affinities toward CB_1_ and CB_2_ receptors acting primarily as CB_1_ receptor antagonists/inverse agonists. We are confident that our straightforward new approach will enable us and others to extensively investigate these bioactive molecules and their targets in future studies.

## Supporting Information

File 1 Experimental and analytical data.
