# Supplementary material for: Iodine-catalyzed electrophilic substitution of indoles: Synthesis of (un)symmetrical diindolylmethanes with a quaternary carbon center
Source: Beilstein J Org Chem. 2021 Jun 18;17:1464–75. doi: 10.3762/bjoc.17.102 (PMC8218542; doi:10.3762/bjoc.17.102)
Supplement: File 1 — Experimental and analytical data. [file Beilstein_J_Org_Chem-17-1464-s001.pdf]

BEILSTEIN JOURNAL OF ORGANIC CHEMISTRY

Supporting Information

for

Iodine-catalyzed electrophilic substitution of indoles: Synthesis of (un)symmetrical diindolylmethanes with a quaternary carbon center

Thanigaimalai Pillaiyar, Masoud Sedaghati, Andhika B. Mahardhika, Lukas L. Wendt and Christa E. Müller

Beilstein J. Org. Chem. **2021,** *17,* 1464–1475. doi:10.3762/bjoc.17.102

Experimental and analytical data

License and Terms: This is a supporting information file under the terms of the Creative Commons Attribution License (https://creativecommons.org/licenses/by/4.0). Please note that the reuse, redistribution and reproduction in particular requires that the author(s) and source are credited and that individual graphics may be subject to special legal provisions.
The license is subject to the *Beilstein Journal of Organic Chemistry* terms and conditions: (https://www.beilstein-journals.org/bjoc/terms)

Entry No:	Table of Contents	Page
1	Radioligand binding assays at CB_1 and CB_2 receptors	S3
2	Functional assays at CB_1 and CB_2 receptors	S3-S4
3	**Figure S1.** Affinities of **3e** and **3ad** at cannabinoid CB_1 and CB_2 receptors	S5
4	**Figure S2.** Functional effects of **3e** and **3ad** at human CB_1 and CB_2 receptors	S5
5	**Figure S3.** Antagonistic effects of **3e** and **3ad** at human cannabinoid receptors	S36
6	Experimental section (general information)	S7
	General experimental procedures for the synthesis of trifluoromethylated unsymmetrical 3,3′- bis(indolyl)methanes and Analytical Data	S8-27
7	**Figure S4-S36.** ^{1}H and ^{13}C-NMR spectra of compounds	S28-S60
8	References	S61

Radioligand binding assays at CB_1 and CB_2 receptors

Competition binding assays were performed using the CB receptor agonist radioligand [^{3}H](−)-*cis*-3-[2-hydroxy-4-(1,1-dimethylheptyl)phenyl]-*trans*-4-(3-hydroxypropyl)-cyclohexanol as previously described [S1]. Briefly, membrane preparations of Chinese hamster ovary (CHO) cells stably expressing human CB_1 or CB_2 receptor were used (30 µg of protein/well for CB_1 and 16 µg of protein/well for CB_2-receptor preparations). All stock solutions of test compounds (10 µM) were prepared in DMSO. After 2 h of incubation, a mixture containing 15 µL of the test compound (in DMSO), 60 µL of [^{3}H]CP55,940 solution in assay buffer (50 mM TRIS, 3 mM $MgCl_2$, 0.1% BSA, pH 7.4), 60 µL of membrane preparation (in 50 mM TRIS buffer) and 465 µL of assay buffer, were filtered through GF/C glass fiber filters (presoaked for 0.5 h in 0.3% aq. polyethyleneimine solution), using a Brandel 96-channel cell harvester (Brandel, Gaithersburg, MD). Radioactivity on the filters was then measured in a liquid scintillation counter (Topcount NXT, Packard/Perkin-Elmer) after 10 h of preincubation with 50 µl of scintillation cocktail (Multiscint 25, Perkin-Elmer). For the calculation of K_i values the Cheng-Prusoff equation and K_D values of 2.4 nM ([^{3}H]CP55,940 at CB_1) and 0.7 nM ([^{3}H]CP55,940 at CB_2) were used.

Functional assays at CB_1 and CB_2 receptors

Functional assays of the selected compounds were assessed using the Trupath BRET^2 assay system as recently described [S2]. The Trupath plasmids were a gift from Bryan Roth's Laboratory (Addgene kit #1000000163). Briefly, HEK293 cells were seeded in a 6 well-plate at adensity of 700.000 cells/well and incubated for 4h. Plasmids containing $G\alpha_i$1-Rluc8, ß3 and γ9-GFP2 and either human CB_1 or CB_2 in pcDNA3.1(+) at a ratio of 1:1:1:1 were mixed with Lipofectamine 2000 and transfected according to the manufacturer's protocol. The cells were further incubated overnight, then harvested and seeded at a density of 40.000 cells/well in 96 well-plates with full growth medium, and cultured for another 24h. On the day of the assay,

culture medium was carefully discarded and replaced by 60 μL of HBSS-HEPES buffer, followed by the addition of 10 μL of 50 μM Coelenterazine 400a. For testing of agonistic activity, the test compounds were added to the wells in a volume of 30 μL and incubated for 5 min. For antagonist testing, compounds were added in a volume of 15 μL, incubated for 5 min, and 15 μL of the CB receptor agonist CP,55940 at its EC_{80} was added, and the mixture was incubated for another 5 min. Test compounds were dissolved in DMSO and the final DMSO concentration in the assays was 1%. Finally, the signal was measured using a Mitras LB400 according to previously published literature[S3]. Data were obtained from 3-4independent experiments performed in duplicate.

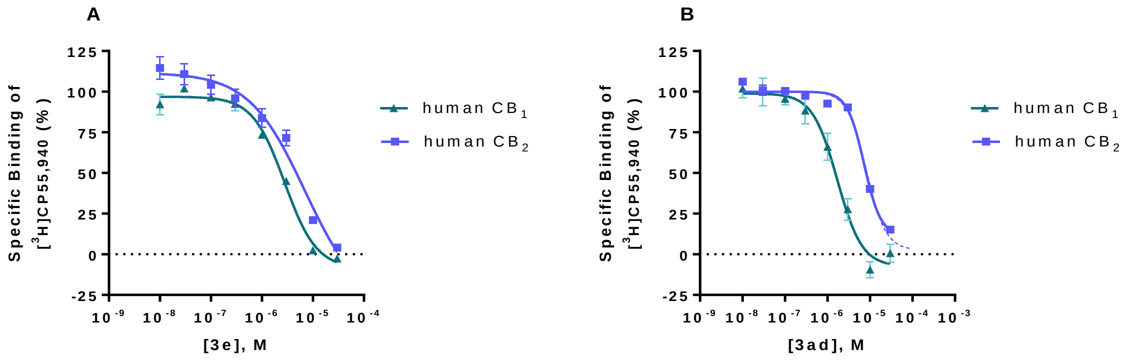

Figure S1. Affinities of **3e** (A) and **3ad** (B) for human cannabinoid CB_1 and CB_2 receptors determined in radioligand binding studies. Data points represent means ± SEM of at least three independent experiments.

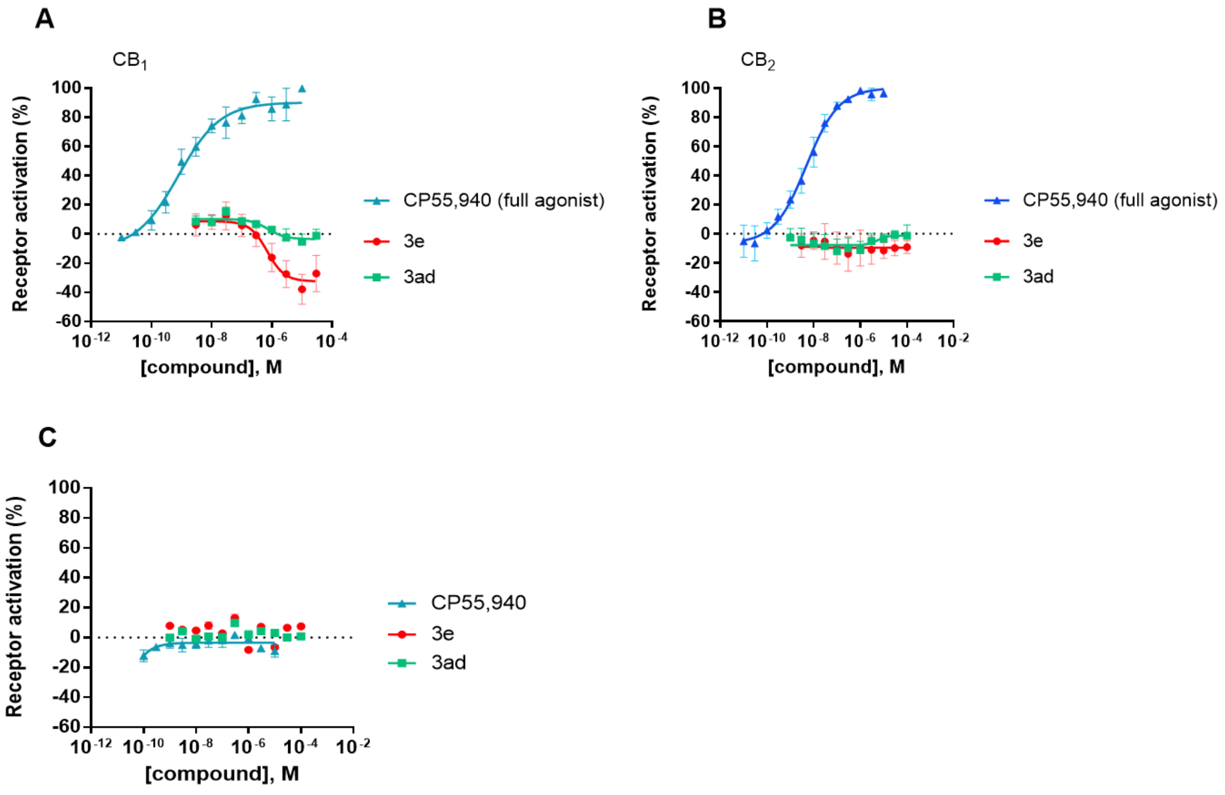

Figure S2. Functional effects of compounds **3e** and **3ad** at human CB_1 (A) and CB_2 (B) receptors, and control HEK293 cells transfected with only Gα$_i$1-Rluc8, ß3 and γ9-GFP2 (C), determined using the Trupath $BRET^2$ assay. The effect of the full agonist CP55,940 was set

as 100% receptor activation (EC_{50} at CB_1 = 0.00105 ± 0.00050 µM; EC_{50} at CB_2 = 0.0100 ± 0.0005 µM). Compounds **3e** and **3ad** showed no agonistic activity, but inverse agonistic activity at the CB_1, not at the CB2 receptor. Data points represent means ± SEM of minimum three independent experiments.

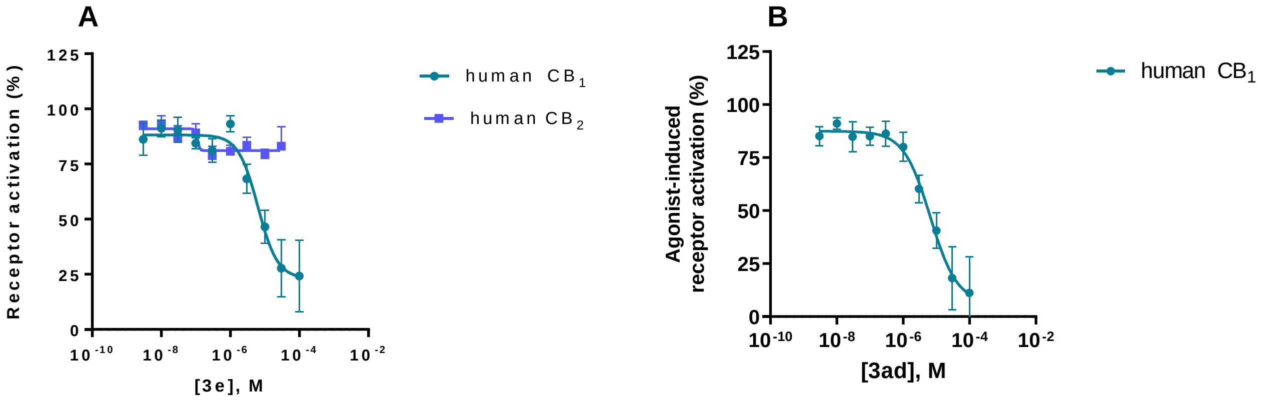

Figure S3. Determination of antagonistic activity of **3e** at human CB_1 and CB_2 (A) and of **3ad** at human CB_1 receptors (B), determined using the Trupath $BRET^2$ assay. Receptor stimulation was achieved using CP55,940 at a concentration representing its EC_{80} value at the respective receptor. Data points represent means ± SEM of at least three independent experiments.

Experimental Section

General information

Chemicals were purchased from Merck (Darmstadt, Germany), ABCR (Karlsruhe, Germany), or TCI (Eschborn, Germany). Thin layer chromatography (TLC) was performed on TLC plates F_{254} (Merck) and analyzed using UV light. An LCMS instrument coupled to electrospray ionisation mass spectrometry (LC ESI-MS) was used to determine the purities of the isolated products using the following procedure: the compounds were dissolved at a concentration of 1.0 mg/mL in acetonitrile, containing 2 mM NH_4CH_3COO. Then, 10 µL of the sample was injected into an HPLC column (Phenomenex Luna 3 µ C18, 50 × 2.00 mm). Elution was performed with a gradient of water: methanol (containing 2 mM NH_4CH_3COO) from 90:10 to 0:100 starting the gradient immediately at a flow rate of 250 µL/min for 15 min followed by washing with 100% methanol for another 15 min. UV absorption was detected from 200 to 600 nm using a diode array detector (DAD). The purity of the compounds was determined at 220−400 nm and was ≥95% for all products. ^{1}H, ^{13}C and ^{19}F NMR data were measured in $CDCl_3$ or DMSO-d_6 as a solvent. Chemical shifts are reported in parts per million (ppm) relative to the deuterated solvents (DMSO-d_6), ^{1}H: 2.49 ppm, ^{13}C: 39.70 ppm; ($CDCl_3$) ^{1}H: 7.25 ppm, ^{13}C: 77.17 ppm; coupling constants J are given in Hertz and spin multiplicities are given as s (singlet), d (doublet), t (triplet), q (quartet), sext (sextet), m (multiplet), br (broad). HRMS was recorded on a micrOTOF-Q mass spectrometer (Bruker) with ESI-source coupled with an HPLC Dionex Ultimate 3000 (Thermo Scientific) using an EC 50/2 Nucleodur C18 Gravity 3 µm column (MachereyNagel). The column temperature was 425 °C. Ca. 1 µL of a 1 mg/mL solution of the sample in acetonitrile was injected and a flow rate of 0.3 mL/min was used. HPLC was started with a solution of acetonitrile in water (10:90), containing 2 mM CH_3COONH_4. The gradient was started after 1 min reaching 100% acetonitrile within 9 min

and then flushed with this concentration for another 5 min. Melting points were measured on a melting point apparatus (BÜCHI melting point B-545) and are uncorrected.

General Experimental Procedure for the Synthesis of diindolylmethanes 3: To the solution of **1** [S4] (0.2 mmol) and appropriate indole derivatives (**2**, 0.2 mmol) in acetonitrile (5 mL), I_2 (0.1 eq., 10%) was added at rt. The mixture was heated to 40 °C. The reaction was monitored by TLC with UV detection. After the reaction was completed, the mixture was poured onto ice water, and extracted with EtOAc (2 x 25 mL). The combined organic layers were washed with brine, dried with $MgSO_4$, filtered, and concentrated under reduced pressure. The crude product was purified by silica gel column chromatography.

5-Methoxy-3-(2,2,2-trifluoro-1-(4-methoxy-1*H*-indol-3-yl)-1-phenylethyl)-1*H*-indole (3b): The compound **3b** was synthesized by the reaction of 2,2,2-trifluoro-1-(5-methoxy-1*H*-indol-3-yl)-1-phenylethan-1-ol (**1a**, 64 mg) and 4-methoxy-1*H*-indole (**1h**, 30 mg, 0.2 mmol) in acetonitrile (5 mL) in the presence of iodine (5 mg, 10 mol%). The product was isolated as brown solid (60 mg, 67%). M.p: 110.5 °C. ^{1}H NMR (600 MHz, DMSO-d_6) δ [ppm] = 11.22 (s, 1H, NH), 10.89 (s, 1H, NH), 7.43 – 7.30 (m, 6H), 7.07 (d, $^{4}J_{H,H}$, 1H), 7.02 – 6.99 (m, 1H), 6.70 (dd, $^{3}J_{H,H}$ = 8.8 Hz, $^{4}J_{H,H}$ = 2.4 Hz, 1H), 6.49 – 6.46 (m, 1H), 6.08 (d, $^{4}J_{H,H}$ = 2.4 Hz, 1H), 5.88 (d, $^{4}J_{H,H}$ = 2.4 Hz, 1H) ,3.83 – 3.76 (m, 1H), 3.79 (s, 3H, CH_3), 3.25 (s, 3H, CH_3). ^{13}C NMR (151 MHz, DMSO-d_6) δ [ppm] = 152.8, 152.5, 138.1, 138.1, 134.4, 131.6, 128.8 (2C, CH), 128.39 (q, $^{1}J_{C,F}$ = 266.2 Hz, 1C, CF_3), 128.2, 128.0, 126.6, 126.3, 122.4, 117.0, 112.3, 111.1, 111.0, 105.2, 102.4, 101.2, 99.0, 55.73 (q, $^{2}J_{C,F}$ = 25.4, 1C, C_q), 54.8 (1C, CH_3), 54.7 (1C, CH_3). ^{19}F NMR (565 MHz, DMSO-d_6) *δ* [ppm] = -63.29 (s, 3F, CF_3). LC-MS: positive [*m/z*] = 451.3 $[M+H]^+$. Purity by HPLC-UV (254 nm) ESI-MS: 96.0%. HRMS (ESI-QTOF) calculated for $C_{26}H_{21}F_3N_2O_2$ $[M + H]^+$: 451.1633 found: 451.1630.

3-(2,2,2-Trifluoro-1-(5-methoxy-1*H*-indol-3-yl)-1-phenylethyl)-1*H*-indole-4-carbonitrile (3c): The compound **3c** was synthesized by the reaction of 2,2,2-trifluoro-1-(5-methoxy-1*H*-indol-3-yl)-1-phenylethan-1-ol (**1a**, 64 mg, 0.2 mmol) and 4-cyanoindole (**2c**, 28 mg, 0.2 mmol) in acetonitrile (5 mL) in the presence of iodine (5 mg, 10 mol%). The product was isolated as white solid (58 mg, 65%). M.p: 201-203 °C. ^{1}H NMR (600 MHz, DMSO-d_6) δ [ppm] = 11.91 (s, 1H, NH), 11.13 (s, 1H, NH), 7.81 (dd, $^{3}J_{H,H}$ = 8.1 Hz, $^{4}J_{H,H}$ = 1.1 Hz, 1H), 7.60 – 7.54 (m, 2H), 7.48 (s, 1H), 7.39 – 7.31 (m, 3H), 7.28 – 7.15 (m, 4H), 6.63 (dd, $^{3}J_{H,H}$ = 8.7 Hz, $^{4}J_{H,H}$ = 2.4 Hz, 1H), 6.17 (s, 1H), 3.36 (s, 3H, CH_3). ^{13}C NMR (151 MHz, DMSO-d_6) δ [ppm] = 152.5, 138.6, 137.5, 131.8, 129.7, 129.60 (q, $^{1}J_{C,F}$ = 282.9, 1C, CF_3), 129.5, 128.5, 128.3, 128.0 (2C, CH), 127.7, 126.5, 124.5, 121.0, 118.7, 117.3, 112.6, 112.1, 110.9, 110.6, 103.4, 102.9, 55.05 (q, $^{2}J_{C,F}$ = 25.8 Hz, 1C, C_q), 54.8 (1C, CH_3). ^{19}F NMR (565 MHz, DMSO-d_6) δ [ppm] = -63.36 (s, 3F, CF_3). LC-MS: positive [*m/z*] = 446.0 ($[M+H]^+$. Purity by HPLC-UV (254 nm) ESI-MS: 99.0%. HRMS (ESI-QTOF) calculated for $C_{26}H_{18}F_3N_3O$ $[M + H]^+$: 446.1480 found: 446.1467.

6-Fluoro-3-(2,2,2-trifluoro-1-(5-methoxy-1*H*-indol-3-yl)-1-phenylethyl)-1*H*-indole (3e): The compound **3e** was synthesized by the reaction of 2,2,2-trifluoro-1-(5-methoxy-1*H*-indol-3-yl)-1-phenylethan-1-ol (**1a**, 64 mg, 0.2 mmol) and 6-fluoro-1*H*-indole (**2e**, 27 mg, 0.22 mmol) in acetonitrile (5 mL) in the presence of iodine (5 mg, 10 mol%). The product was isolated as light brown solid (84 mg, 96%). M.p: 102-104 °C. ^{1}H NMR (600 MHz, DMSO-d_6) δ [ppm] = 11.24 (d, $^{2}J_{H,H}$ = 2.8 Hz, 1H, NH), 11.07 (d, $^{2}J_{H,H}$ = 2.8 Hz, 1H, NH), 7.46 – 7.38 (m, 2H), 7.36 (m, 3H), 7.29 (d, $^{3}J_{H,H}$ = 8.8 Hz, 1H), 7.18 (dd, $^{3}J_{H,H}$ = 9.9 Hz, $^{4}J_{H,H}$ = 2.4 Hz, 1H), 6.94 (d, $^{4}J_{H,H}$ = 2.6 Hz, 2H), 6.83 (dd, $^{2}J_{H,H}$ = 9.0 Hz, $^{2}J_{H,H}$ = 5.5 Hz, 1H), 6.72 – 6.60 (m, 2H), 6.20 – 6.14 (m, 1H), 3.38 (s, 3H, CH_3). ^{13}C NMR (151 MHz, DMSO-d_6) δ [ppm] = 158.42 (d, $^{1}J_{C,F}$ = 235.2 Hz, 1C, CF_{arom}), 152.6, 138.9, 136.8, 136.7, 129.0, 128.0, 128.02 (q, $^{1}J_{C,F}$ = 287.9, 123.5 Hz, 1C, CF_3), 127.6, 127.4, 126.7, 126.3, 122.8, 122.00 (d, $^{2}J_{C,F}$ = 13.8 Hz, 1C, CF_{arom}), 113.3, 112.3, 112.2, 110.8, 107.4, 107.2, 103.0, 97.6, 97.4, 55.09 (q, $^{2}J_{C,F}$ = 24.2 Hz, 1C, C_q), 54.8

(1C, CH_3). ^{19}F NMR (565 MHz, DMSO-d_6) δ [ppm] = -62.49 (s, 3F, CF_3), -120.78 – -123.43 (m, 1F, F_{arom}). LC-MS: positive [*m/z*] = 439.1[M+H]$^+$. Purity by HPLC-UV (254 nm) ESI-MS: 99.0% HRMS (ESI-QTOF) calculated for $C_{25}H_{18}F_4N_2O$ [M + H]$^+$: 439.1434 found: 439.1422.

3-(2,2,2-Trifluoro-1-(5-methoxy-1*H*-indol-3-yl)-1-phenylethyl)-1*H*-indol-5-ol (3f): The compound **3f** was synthesized by the reaction of 2,2,2-trifluoro-1-(5-methoxy-1*H*-indol-3-yl)-1-phenylethan-1-ol (**1a**, 64 mg, 0.2 mmol) and 6-hydroxy-1*H*-indole (**2f**, 27 mg, 0.2 mmol) in acetonitrile (5 mL) in the presence of iodine (5 mg, 10 mol%). The product was isolated (53 mg, 61%) as white solid. M.p: 218-220 °C. ^{1}H NMR (600 MHz, DMSO-d_6) δ [ppm] = 11.19 (d, $^4J_{H,H}$ = 2.8 Hz, 1H, NH), 10.45 (d, $^4J_{H,H}$ = 2.4 Hz, 1H, NH), 8.89 (s, 1H, OH), 7.39 (dd, $^3J_{H,H}$ = 5.5 Hz, $^4J_{H,H}$ = 1.9 Hz, 3H), 7.36 – 7.33 (m, 2H), 7.30 (d, $^3J_{H,H}$ = 8.8 Hz, 1H), 7.23 (d, $^4J_{H,H}$ = 8.5 Hz, 1H), 7.06 (d, $^4J_{H,H}$ = 2.6 Hz, 1H), 6.77 (d, $^4J_{H,H}$ = 2.1 Hz, 1H), 6.68 (dd, $^3J_{H,H}$ = 8.8 Hz, $^4J_{H,H}$ 2.4 Hz, 1H), 6.50 (dd, $^3J_{H,H}$ = 8.4 Hz, $^4J_{H,H}$ = 2.2 Hz, 1H), 5.98 (d, $^4J_{H,H}$ = 2.2 Hz, 1H), 5.90 (d, $^4J_{H,H}$ = 2.4 Hz, 1H), 3.23 (s, 3H, CH_3). ^{13}C NMR (151 MHz, DMSO-d_6) δ [ppm] = 153.3, 152.8, 138.3, 138.0, 133.9, 131.6, 128.8 (2C, CH_3), 128.1 (2C, CH_3), 127.9, 127.23 (q, $^1J_{C,F}$ = 282.8 Hz, 1C, CF_3), 126.6, 126.4, 120.4, 119.9, 112.2, 111.2, 111.0, 109.8, 103.9, 102.4, 96.8, 55.77 (q, $^2J_{C,F}$ = 13.4 Hz, 1C, C_q), 54.6 (1C, CH_3). ^{19}F NMR (565 MHz, DMSO-d_6) δ [ppm] = -63.27 (s, 3F, CF_3). LC-MS: positive [*m/z*] = 437.0 [M+H]$^+$. Purity by HPLC-UV (254 nm) ESI-MS: 99.0%. HRMS (ESI-QTOF) calculated for $C_{25}H_{19}F_3N_2O_2$ [M + H]$^+$: 437.1477 found: 437.1478.

7-Bromo-3-(2,2,2-trifluoro-1-(5-methoxy-1*H*-indol-3-yl)-1-phenylethyl)-1*H*-indole (3g): The compound **3g** was synthesized by the reaction of 2,2,2-trifluoro-1-(5-methoxy-1*H*-indol-3-yl)-1-phenylethan-1-ol (**1a**, 64 mg, 0.2 mmol) and 7-bromo-1*H*-indole (**2g**, 39 mg, 0.2 mmol) in acetonitrile (5 mL) in the presence of iodine (5 mg, 10 mol%). The product was isolated as brown solid (95 mg, 95%). M.p: 104-106 °C. ^{1}H NMR (600 MHz, DMSO-d_6) δ [ppm] = 11.42

(s, 1H, NH), 11.09 (s, 1H, NH), 7.43 – 7.40 (m, 2H), 7.38 – 7.35 (m, 3H), 7.31 – 7.26 (m, 2H), 6.95 – 6.89 (m, 3H), 6.75 (t, $^3J_{H,H}$ = 7.9 Hz, 1H), 6.69 (dd, $^3J_{H,H}$ = 8.9 Hz, $^4J_{H,H}$ = 2.4 Hz, 1H), 3.39 (s, 3H, CH_3). ^{13}C NMR (151 MHz, DMSO) δ [ppm] = 152.8, 138.8, 135.2, 132.0, 129.2, 128.3 (2C, CH_{arom}), 128.24 (q, $^1J_{C,F}$ = 285.0 Hz), 128.0, 127.9, 127.8, 127.0, 126.4, 123.9, 120.8, 120.8, 120.4, 114.7, 112.5, 112.4, 111.1, 104.6, 103.1, 55.45 (1C, CH_3), 55.0 (1C, CH_3). ^{19}F NMR (565 MHz, DMSO-d_6) *δ* [ppm] = -62.39 (s, 3F, CF_3). LC-MS: positive [*m*/*z*] = 499.3 M+H]$^+$. Purity by HPLC-UV (254 nm) ESI-MS: 95.5%. HRMS (ESI-QTOF) calculated for $C_{25}H_{18}BrF_3N_2O$ [M + H]$^+$: 499.0633 found: 499.0630.

5,6-Difluoro-3-(2,2,2-trifluoro-1-(5-methoxy-1*H*-indol-3-yl)-1-phenylethyl)-1*H*-indole (3h): The compound **3h** was synthesized by the reaction of 2,2,2-trifluoro-1-(5-methoxy-1*H*-indol-3-yl)-1-phenylethan-1-ol (**1a**, 64 mg, 0.22 mmol) and 5,6-difluoro-1*H*-indole (**2h**, 31 mg, 0.2 mmol) in acetonitrile (5 mL) in the presence of iodine (5 mg, 10 mol%). The product was isolated as yellow solid (83 mg, 91%). M.p: 205-207 °C. ^{1}H NMR (600 MHz, DMSO-d_6) δ [ppm] = 11.38 (s, 1H, NH), 11.10 (s, 1H, NH), 7.44 – 7.35 (m, 6H), 7.29 (d, $^3J_{H,H}$ = 8.8 Hz, 1H), 7.07 (d, $^4J_{H,H}$ = 2.6 Hz, 1H), 6.96 (d, $^4J_{H,H}$ = 2.6 Hz, 1H), 6.68 (dd, $^3J_{H,H}$ = 8.8, $^4J_{H,H}$ = 2.4 Hz, 1H), 6.53 (m, 1H), 6.12 (d, $^4J_{H,H}$ = 2.4 Hz, 1H), 3.37 (s, 3H, CH_3). ^{13}C NMR (151 MHz, DMSO-d_6) δ [ppm] = 152.7, 146.25 (dd, $^1J_{C,F}$ = 238.4 Hz, $^2J_{C,F}$ = 15.7 Hz, 1C, CF_{arom}), 144.54 (dd, $^1J_{C,F}$ = 233.8 Hz, $^2J_{C,F}$ = 14.7 Hz, 1C, CF_{arom}), 138.5, 131.9, 131.87 (d, $^3J_{C,F}$ = 10.6 Hz, 1C, C_q), 129.0, 128.6, 128.1 (2C, CH), 128.05 (d, $^1J_{C,F}$ = 287.4 Hz, 1C, CF_3), 127.8, 126.8, 126.2, 121.41 (d, $^3J_{C,F}$ = 8.2 Hz, 1C, CH_{arom}), 113.3, 112.3, 112.0, 110.9, 107.02 (d, $^2J_{C,F}$ = 19.8 Hz, 1C, CH_{arom}),102.9, 99.6, 99.5, 54.83 (d, $^2J_{C,F}$ = 26.2 Hz, 1C, C_q). 54.8 (1C, CH_3). ^{19}F NMR (565 MHz, DMSO-d_6) *δ* [ppm] = -62.73 (s, 3F, CF_3) , -145.02 – -145.50 (m, 1F, F_{arom}), -148.00 – -148.43 (m, 1F, F_{arom}). LC-MS: positive [*m*/*z*] = 457.2 [M+H]$^+$. Purity by HPLC-UV (254 nm) ESI-MS: 99.0%. HRMS (ESI-QTOF) calculated for $C_{25}H_{17}F_5N_2O$ [M + H]$^+$: 457.1339 found: 457.1325.

4-Methoxy-3-(2,2,2-trifluoro-1-(1*H*-indol-3-yl)-1-phenylethyl)-1*H*-indole (3i): The compound **3i** was synthesized by the reaction of 2,2,2-trifluoro-1-(4-methoxy-1*H*-indol-3-yl)-1-phenylethan-1-ol (**1b**, 64 mg, 0.2 mmol) and 1*H*-indole (**2a**, 24 mg, 0.2 mmol) in acetonitrile (5 mL) in the presence of iodine (5 mg, 10 mol%). The product was isolated as dark brown solid (65 mg, 77%). M.p: 134-136 °C. ^{1}H NMR (600 MHz, DMSO-d_6) δ [ppm] = 11.19 (s, 1H, NH), 11.01 (s, 1H, NH), 7.55 – 7.48 (m, 1H), 7.45 – 7.31 (m, 2H), 7.30 – 7.21 (m, 4H), 7.05 – 6.97 (m, 2H), 6.96 – 6.82 (m, 2H), 6.81 – 6.74 (m, 2H), 6.17 (d, $^{3}J_{H,H}$ = 7.8, Hz, 1H), 2.91 (s, 3H, CH_3). ^{13}C NMR (151 MHz, DMSO-d_6) δ [ppm] = 153.2, 140.7, 138.2, 136.7, 129.5, 127.4, 127.01 (d, $^{1}J_{C,F}$ = 287.9 Hz, 1C, CF_3), 126.9, 126.7, 126.1, 123.7, 122.3, 121.0, 120.4, 118.5, 116.7, 113.8, 113.5, 113.1, 111.8, 111.6, 104.6, 100.3, 55.99 (d, $^{2}J_{C,F}$ = 25.5 Hz, 1C, C_q), 53.8 (1C, CH_3). ^{19}F NMR (565 MHz, DMSO-d_6) δ [ppm] = -62.73 (s, 3F, CF_3). LC-MS: positive [m/z] = 421.2 $[M+H]^+$. Purity by HPLC-UV (254 nm) ESI-MS: 98.0%. HRMS (ESI-QTOF) calculated for $C_{25}H_{19}F_3N_2O$ $[M + H]^+$: 421.1528 found: 421.1521.

5-Fluoro-3-(2,2,2-trifluoro-1-(4-methoxy-1*H*-indol-3-yl)-1-phenylethyl)-1*H*-indole (3j): The compound **3j** was synthesized by the reaction of 2,2,2-trifluoro-1-(4-methoxy-1*H*-indol-3-yl)-1-phenylethan-1-ol (**1a**, 64 mg, 0.2 mmol) and 5-fluoro-1*H*-indole (**2d**, 84 mg, 0.62 mmol) in acetonitrile (5 mL) in the presence of iodine (5 mg, 10 mol%). The product was isolated as brown solid (78 mg, 89%). M.p: 124-126 °C. ^{1}H NMR (600 MHz, DMSO-d_6) δ [ppm] = 11.23 (d, $^{4}J_{H,H}$ = 2.8 Hz, 1H, NH), 11.15 (d, $^{4}J_{H,H}$ = 3.1 Hz, 1H, NH), 7.50 (d, $^{3}J_{H,H}$ = 7.5 Hz, 2H), 7.35 (dd, $^{3}J_{H,H}$ = 8.8 Hz, $^{4}J_{H,H}$ = 4.9 Hz, 1H), 7.31 – 7.25 (m, 4H), 7.02 – 6.98 (m, 2H), 6.93 (t, $^{3}J_{H,H}$ = 7.9 Hz, 1H), 6.86 (td, $^{3}J_{H,H}$ = 9.0 Hz, $^{4}J_{H,H}$ = 2.6 Hz, 1H), 6.80 – 6.73 (m, 1H), 6.17 (d, $^{3}J_{H,H}$ = 7.7 Hz, 1H), 2.91 (s, 3H, CH_3). ^{13}C NMR (151 MHz, DMSO-d_6) δ [ppm] = 156.27 (d, $^{1}J_{C,F}$ = 229.8 Hz, 1C, CF_{arom}), 153.2, 140.2, 138.2, 133.3, 129.4, 129.3, 129.27 (q, $^{1}J_{C,F}$ = 283.9 Hz, 1C,CF_3), 127.1, 126.8, 126.34 (d, $^{4}J_{C,F}$ = 10.8 Hz, 1C, CH_{arom}), 123.6, 122.4, 116.7, 113.9, 113.0, 112.4, 112.4, 108.7, 108.6, 105.93 (dd, $^{2}J_{C,F}$ = 27.3 Hz, $^{3}J_{C,F}$ = 7.0 Hz, 1C, CH_{arom}), 104.7,

100.3, 55.70 (q, $^2J_{C,F}$ = 30.3 Hz, 1C, C_q), 53.8 (1C, CH_3). ^{19}F NMR (565 MHz, DMSO-d_6) δ [ppm] = -63.28 (s, 3F, CF_3), -124.18 – -126.25 (m, 1F, F_{arom})). LC-MS: positive [*m/z*] = 439.2 $[M+H]^+$. Purity by HPLC-UV (254 nm) ESI-MS: 97%. HRMS (ESI-QTOF) calculated for $C_{25}H_{18}F_4N_2O$ $[M + H]^+$: 439.1434 found: 439.1433.

6-Methoxy-3-(2,2,2-trifluoro-1-(1*H*-indol-3-yl)-1-phenylethyl)-1*H*-indole (3k): The compound **3k** was synthesized by the reaction of 2,2,2-trifluoro-1-(6-methoxy-1*H*-indol-3-yl)-1-phenylethan-1-ol (**1c**, 64 mg, 0.2 mmol) and 1*H*-indole (**2a**, 24 mg, 0.2 mmol) in acetonitrile (5 mL) in the presence of iodine (5 mg, 10 mol%). The product was isolated (59 mg, 70%) as light brown solid. M.p: 89-91 °C. ^{1}H NMR (600 MHz, DMSO-d_6) δ [ppm] = 11.16 (s, 1H, NH), 10.95 (s, 1H, NH), 7.46 – 7.30 (m, 6H), 7.02 (t, $^3J_{H,H}$ = 7.5 Hz, 1H), 6.94 – 6.85 (m, 3H), 6.80 – 6.71 (m, 3H), 6.44 (dd, $^3J_{H,H}$ = 8.9 Hz, $^4J_{H,H}$ = 2.4 Hz, 1H), 3.72 (s, 3H, CH_3). ^{13}C NMR (151 MHz, DMSO-d_6) δ [ppm] = 155.2, 139.2, 137.5, 136.7, 131.05 (q, $^1J_{H,H}$ = 283.1 Hz, 1C, CF_3), 129.0 (2C, CH), 127.9 (2C, CH), 127.5, 126.5, 125.9, 125.2, 121.6, 121.0, 120.9, 120.2, 118.7, 113.2, 113.1, 111.7, 109.0, 94.5, 55.19 (q, $^2J_{H,H}$ = 25.2 Hz, 1C, C_q), 55.0 (1C, CH_3). ^{19}F NMR (565 MHz, DMSO-d_6) δ [ppm] = - 62.30 (s, 3F, CF_3). LC-MS: positive [*m/z*] = 421.2 $[M+H]^+$. Purity by HPLC-UV (254 nm) ESI-MS: 99.0%. HRMS (ESI-QTOF) calculated for $C_{25}H_{19}F_3N_2O$ $[M + H]^+$: 421.1528 found: 421.1520.

7-Bromo-3-(2,2,2-trifluoro-1-(6-methoxy-1*H*-indol-3-yl)-1-phenylethyl)-1*H*-indole (3l): The compound **3l** was synthesized by the reaction of 2,2,2-trifluoro-1-(6-methoxy-1*H*-indol-3-yl)-1-phenylethan-1-ol (**1c**, 64 mg, 0.2 mmol) and 7-bromo-1*H*-indole (**2g**, 39 mg, 0.2 mmol) in acetonitrile (5 mL) in the presence of iodine (5 mg, 10 mol%). The product was isolated as brown solid (72 mg, 72%). M.p: 92-94 °C. ^{1}H NMR (600 MHz, DMSO-d_6) δ [ppm] = 11.42 (s, 1H, NH), 10.99 (s, 1H, NH), 7.37 (m, 5H), 7.27 (d, $^3J_{H,H}$ = 7.5 Hz, 1H), 6.97 – 6.85 (m, 3H), 6.78 – 6.70 (m, 3H), 6.46 (dd, $^3J_{H,H}$ = 8.9 Hz, $^4J_{H,H}$ = 2.4 Hz, 1H), 3.73 (s, 3H, CH_3). ^{13}C NMR (151 MHz, DMSO-d_6) δ [ppm] = 155.2, 138.8, 137.5, 134.9, 129.0, 128.9, 128.14 (q, $^1J_{H,H}$ =

283.4 Hz, 1C, CF_3), 128.0 (2C, CH), 127.7, 127.6, 127.6, 125.3, 123.7, 121.4, 120.6, 120.2, 120.1, 114.6, 112.8, 109.2, 104.4, 94.6, 55.23 (q, $^2J_{H,H}$ = 24.6 Hz, 1C, C_q), 55.0 (1C, CH_3). ^{19}F NMR (565 MHz, DMSO-d_6) δ [ppm] = -62.30 (s, 3F, CF_3). LC-MS: positive [*m*/*z*] = 499.3 $[M+H]^+$. Purity by HPLC-UV (254 nm) ESI-MS: 99%. HRMS (ESI-QTOF) calculated for $C_{25}H_{18}BrF_3N_2O$ $[M + H]^+$: 499.0633 found: 499.0639.

5,6-Difluoro-3-(2,2,2-trifluoro-1-(6-fluoro-1*H*-indol-3-yl)-1-phenylethyl)-1*H*-indole (3m): The compound **3m** was synthesized by the reaction of 2,2,2-trifluoro-1-(6-fluoro-1*H*-indol-3-yl)-1-phenylethan-1-ol (**1d**, 62 mg, 0.2 mmol) and 5,6-difluoro-1*H*-indole (**2h**, 99 mg, 0.62 mmol) in acetonitrile (5 mL) in the presence of iodine (5 mg, 10 mol%). The product was isolated as dark yellow solid (81 mg, 89%). M.p: 167-169 °C. ^{1}H NMR (600 MHz, DMSO-d_6) δ [ppm] = 11.41 (s, 1H, NH), 11.30 (s, 1H, NH), 7.42 (dd, $^3J_{H,H}$ = 11.0 Hz, $^3J_{H,H}$ = 7.2 Hz, 1H), 7.37 (m, 5H), 7.19 (dd, $^3J_{H,H}$ = 9.8 Hz, $^4J_{H,H}$ = 2.5 Hz, 1H), 7.08 (d, $^4J_{H,H}$ = 2.5 Hz, 1H), 6.97 (d, $^4J_{H,H}$ = 2.5 Hz, 1H), 6.77 (dd, $^3J_{H,H}$ = 9.0 Hz, $^4J_{H,H}$ = 5.4 Hz, 1H), 6.65 (td, $^3J_{H,H}$ = 9.0 Hz, $^4J_{H,H}$ = 2.5 Hz, 1H), 6.51 (dd, $^3J_{H,H}$ = 12.3 Hz, $^4J_{H,H}$ = 8.0 Hz, 1H). ^{13}C NMR (151 MHz, DMSO-d_6) δ [ppm] = 158.65 (d, $^1J_{C,F}$ = 235.5 Hz, 1C, CF_{arom}), 146.46 (dd, $^1J_{C,F}$ = 238.5 Hz, $^2J_{C,F}$ = 16.1 Hz, 1C, CF_{arom}), 144.75 (dd, $^1J_{C,F}$ = 233.8 Hz, $^2J_{C,F}$ = 15.1 Hz, 1C, CF_{arom}), 138.6, 136.89 (d, $^2J_{C,F}$ = 13.0 Hz, 1C, CH_{arom}), 132.00 (d, $^2J_{C,F}$ = 11.1 Hz, 1C, CH_{arom}), 129.1, 128.6, 128.4 (2C, CH), 128.31 (q, $^1J_{C,F}$ = 281.6 Hz, 1C, C_q), 128.1, 127.4, 122.8, 121.9, 121.8, 121.50 (d, $^3J_{C,F}$ = 9.0 Hz, 1C, C_q), 113.28 (m, 1C, C_q), 112.9, 107.7, 107.6, 107.2, 99.8, 99.7, 97.7, 55.09 (q, $^2J_{C,F}$ = 26.3 Hz, 1C, C_q). ^{19}F NMR (565 MHz, DMSO-d_6) δ [ppm] = -62.73 (s, 3F, CF_3) , -121.91 (m, 1F, F_{arom}), -145.11 – -145.34 (m, 1F, F_{arom}), -148.12 (m, 1F, F_{arom}). LC-MS: positive [*m*/*z*] = 445 $[M+H]^+$. Purity by HPLC-UV (254 nm) ESI-MS: 99%. HRMS (ESI-QTOF) calculated for $C_{24}H_{14}F_6N_2$ $[M + H]^+$: 445.1139 found: 445.1129.

5,6-Difluoro-3-(2,2,2-trifluoro-1-(1*H*-indol-3-yl)-1-phenylethyl)-1*H*-indole (3n): The compound **3n** was synthesized by the reaction of 2,2,2-trifluoro-1-(1*H*-indol-3-yl)-1-phenylethan-1-ol (**1e**, 58 mg, 0.2 mmol) and 5,6-difluoro-1*H*-indole (**2h**, 30 mg, 0.2 mmol) in acetonitrile (5 mL) in the presence of iodine (5 mg, 10 mol%). The product was isolated as brown solid (61 mg, 99%). M.p: 188-190 °C. ^{1}H NMR (600 MHz, DMSO-d_6) δ [ppm] = 11.38 (s, 1H, NH), 11.23 (s, 1H, NH), 7.44 – 7.35 (m, 7H), 7.08 (d, $^4J_{H,H}$ = 2.6 Hz, 1H), 7.03 (m, 1H), 6.94 (d, $^4J_{H,H}$ = 2.6 Hz, 1H), 6.83 (d, $^3J_{H,H}$ = 8.2 Hz, 1H), 6.76 (m, 1H), 6.52 (m, 1H). ^{13}C NMR (151 MHz, DMSO-d_6) δ [ppm] = 146.24 (dd, $^1J_{C,F}$ = 238.4 Hz, $^2J_{C,F}$ = 15.1 Hz, 1C, CF_{arom}), 144.52 (dd, $^1J_{C,F}$ = 233.7 Hz, $^2J_{C,F}$ = 15.1 Hz, 1C, CF_{arom}), 138.6, 136.7, 131.80 (q, $^3J_{C,F}$ = 11.0 Hz, 1C, C_q), 128.9, 128.4, 128.2 (q, $^1J_{C,F}$ = 283.1 Hz, 1C, CF_3), 128.1 (2C, CH), 127.8, 126.5, 125.8, 121.36 (d, $^3J_{C,F}$ = 11.0 Hz, 1C, C_q), 121.1, 120.7, 118.8, 113.3, 112.5, 111.9, 106.95 (d, $^2J_{C,F}$ = 23.4 Hz, 1C, C_q), 99.5, 99.4, 54.98 (d, $^2J_{C,F}$ = 26.4 Hz, 1C, C_q). ^{19}F NMR (565 MHz, DMSO-d_6) *δ* [ppm] = -62.60 (s, 3F, CF_3) , -145.23 – -145.38 (m, 1F, F_{arom}), -148.16 – -148.30 (m, 1F, F_{arom}). LC-MS: positive [*m*/*z*] = 427. $[M+H]^+$. Purity by HPLC-UV (254 nm) ESI-MS: 99%. HRMS (ESI-QTOF) calculated for $C_{24}H_{15}F_5N_2$ $[M + H]^+$: 427.1234 found: 427.1239.

2-Methyl-3-(2,2,2-trifluoro-1-(1*H*-indol-3-yl)-1-phenylethyl)-1*H*-indole (3o): The compound **3o** was synthesized by the reaction of 2,2,2-trifluoro-1-(1*H*-indol-3-yl)-1-phenylethan-1-ol (**1e**, 58 mg, 0.2 mmol) and 2-methyl-1*H*-indole (**1m**, 26 mg, 0.2 mmol) in acetonitrile (5 mL) in the presence of iodine (5 mg, 10 mol%). The product was isolated (0.246 g, 87%) as white solid. M.p: 206.4 °C. ^{1}H NMR (600 MHz, DMSO-d_6) δ [ppm] = 11.16 (s, 1H, NH), 11.07 (s, 1H, NH), 7.58 – 7.52 (m, 2H), 7.37 (d, $^3J_{H,H}$ = 8.2 Hz, 1H), 7.33 – 7.24 (m, 4H), 7.12 – 7.07 (m, 1H), 7.01 (t, $^3J_{H,H}$ = 7.6 Hz, 1H), 6.97 – 6.91 (m, 2H), 6.75 (t, $^3J_{H,H}$ = 8.3 Hz,1H), 6.66 – 6.62 (m, 1H), 6.53 (d, $^3J_{H,H}$ = 8.2 Hz, 1H), 1.57 (s, 3H, CH_3). ^{13}C NMR (151 MHz, DMSO-d_6) δ [ppm] = 140.5, 136.5, 135.8, 134.7, 128.6, 128.6, 127.9 (2C, CH), 127.8, 128.2 (q, $^1J_{C,F}$ = 282.2 Hz, 1C, CF_3), 127.1, 126.2, 124.7, 120.9, 120.8, 120.7, 119.8, 118.8, 118.2,

114.3, 111.7, 110.3, 107.4, 55.69 (q, $^2J_{C,F}$ = 25.5 Hz, 1C, C_q), 13.5. ^{19}F NMR (565 MHz, DMSO-d_6) δ [ppm] = -60.8 (s, 3F, CF3). LC-MS: positive [*m/z*] = 405 $[M+H]^+$. Purity by HPLC-UV (254 nm) ESI-MS: 99.0%. HRMS (ESI-QTOF) calculated for $C_{25}H_{19}F_3N_2$ [M + H]$^+$: 405.1579 found: 405.1573.

5-Fluoro-3-(2,2,2-trifluoro-1-(5-methoxy-1*H*-indol-3-yl)-1-(p-tolyl)ethyl)-1*H*-indole (3p): The compound **3p** was synthesized by the reaction of 2,2,2-trifluoro-1-(5-methoxy-1*H*-indol-3-yl)-1-(p-tolyl)ethan-1-ol (**1f**, 67 mg, 0.2 mmol) and 5-fluoro-1*H*-indole (**2d**, 27 mg, 0.20 mmol) in acetonitrile (5 mL) in the presence of iodine (5 mg, 10 mol%). The product was isolated red brown solid (90 mg, 99%). M.p: 94-96 °C. ^{1}H NMR (600 MHz, DMSO-d_6) δ [ppm] = 11.30 (s, 1H, NH), 11.06 (s, 1H, NH), 7.40 (dd, $^3J_{H,H}$ = 8.9 Hz, $^4J_{H,H}$ = 4.8 Hz, 1H), 7.29 (d, $^3J_{H,H}$ = 8.4 Hz, 3H), 7.17 (d, $^3J_{H,H}$ = 8.1 Hz, 2H), 7.05 (d, $^4J_{H,H}$ = 2.7 Hz, 1H), 6.95 (d, $^4J_{H,H}$ = 2.6 Hz, 1H), 6.88 (td, $^3J_{H,H}$ = 9.0 Hz, $^4J_{H,H}$ = 2.6 Hz, 1H), 6.68 (dd, $^3J_{H,H}$ = 8.8 Hz, $^4J_{H,H}$ = 2.4 Hz, 1H), 6.46 (dd, $^3J_{H,H}$ = 11.1 Hz, $^4J_{H,H}$ = 2.5 Hz, 1H), 6.16 (d, $^4J_{H,H}$ = 2.4 Hz, 1H), 3.38 (s, 3H, CH_3), 2.31 (s, 3H, CH_3). ^{13}C NMR (151 MHz, DMSO-d_6) δ [ppm] = 156.28 (d, $^1J_{C,F}$ = 231.0 Hz, 1C, CF_{arom}), 152.6, 136.8, 135.7, 133.4, 131.8, 129.20 (q, $^1J_{C,F}$ = 283.3 Hz, 1C, CF_3), 129.0, 128.6, 128.5, 126.7, 126.3, 126.18 (q, $^3J_{C,F}$ = 10.6 Hz, 1C, C_q), 113.28 (q, $^3J_{C,F}$ = 6.0 Hz, 1C, C_q), 112.8, 112.7, 112.3, 112.2, 110.8, 109.4, 109.2, 105.45 (d, $^2J_{C,F}$ = 27.3 Hz, 1C, CH_{arom}), 103.0, 54.8 (1C, CH_3), 54.67 (q, $^2J_{C,F}$ = 26.1 Hz, 1C, C_q), 20.5 (1C, CH_3). ^{19}F NMR (565 MHz, DMSO-d_6) δ [ppm] = -62.76 (s, 3F, CF_3), -122.68 – -126.04 (m, 1F, F_{arom}). LC-MS: positive [*m/z*] = 453.2 $[M+H]^+$. Purity by HPLC-UV (254 nm) ESI-MS: 99.0%. HRMS (ESI-QTOF) calculated for $C_{26}H_{20}F_4N_2O$ [M + H]$^+$: 453.1590 found: 453.1583.

6-Fluoro-3-(2,2,2-trifluoro-1-(5-methoxy-1*H*-indol-3-yl)-1-(p-tolyl)ethyl)-1*H*-indole (3q): The compound **3q** was synthesized by the reaction of 2,2,2-trifluoro-1-(5-methoxy-1*H*-indol-3-yl)-1-(p-tolyl)ethan-1-ol (**1f**, 67 mg, 0.2 mmol) and 5-fluoro-1*H*-indole (**2e**, 27 mg, 0.20 mmol) in acetonitrile (5 mL) in the presence of iodine (5 mg, 10 mol%). The product was

isolated as brown solid (89.mg, 98%). M.p: 92-94 °C. ^{1}H NMR (600 MHz, DMSO-d_6) δ [ppm] = 11.22 (s, 1H, NH), 11.05 (s, 1H, NH), 7.28 (m, 3H), 7.21 – 7.12 (m, 3H), 6.94 (dd, $^3J_{H,H}$ = 6.3 Hz, $^4J_{H,H}$ = 2.6 Hz, 2H), 6.84 (m, 1H), 6.71 – 6.61 (m, 2H), 6.18 (d, $^4J_{H,H}$ = 2.4 Hz, 1H), 3.39 (s, 3H), 2.30 (s, 3H). ^{13}C NMR (151 MHz, DMSO-d_6) δ [ppm] = 152.6, 158.45 (d, $^1J_{C,F}$ = 235.1 Hz, 1C, CF_{arom}), 136.8, 136.71 (d, $^2J_{C,F}$ = 12.4 Hz, 1C, CH_{arom}), 135.9, 131.8, 129.22 (d, $^1J_{C,F}$ = 283.2 Hz, 1C, CF_3), 129.0, 128.6, 127.3, 126.7, 126.4, 122.09 (d, $^2J_{C,F}$ = 13.6 Hz, 1C, CH_{arom}), 122.8, 113.5, 112.5, 112.2, 110.8, 107.4, 107.2, 103.1, 97.6, 97.4, 54.9, 54.67 (d, $^2J_{C,F}$ = 26.3 Hz, 1C, C_q), 20.5 (1C, CH_3). ^{19}F NMR (565 MHz, DMSO-d_6) δ [ppm] = -62.68 (s, 3F, CF_3), -121.52 – -122.62 (m, 1F, F_{arom}). LC-MS: positive [*m*/*z*] = 453.2 $[M+H]^+$. Purity by HPLC-UV (254 nm) ESI-MS: 99%. HRMS (ESI-QTOF) calculated for $C_{26}H_{20}F_4N_2O$ $[M + H]^+$: 453.1590 found: 453.1583.

5,6-Difluoro-3-(2,2,2-trifluoro-1-(4-fluorophenyl)-1-(5-methoxy-1*H*-indol-3-yl)ethyl)-1*H*-indole (3r): The compound **3r** was synthesized by the reaction of 2,2,2-trifluoro-1-(4-fluorophenyl)-1-(5-methoxy-1*H*-indol-3-yl)ethan-1-ol (**1g**, 68 mg, 0.2 mmol) and 5,6-difluoro-1*H*-indole (**2h**, 31 mg, 0.2 mmol) in acetonitrile (5 mL) in the presence of iodine (5 mg, 10 mol%). The product was isolated as brown solid (83 mg, 87%). M.p: 102-104 °C. ^{1}H NMR (600 MHz, DMSO-d_6) δ [ppm] = 11.41 (s, 1H, NH), 11.14 (s, 1H, NH), 7.46 – 7.39 (m, 3H), 7.31 (d, $^3J_{H,H}$ = 8.8 Hz, 1H), 7.25 – 7.18 (m, 2H), 7.09 (d, $^4J_{H,H}$ = 2.7 Hz, 1H), 6.99 (d, $^4J_{H,H}$ = 2.7 Hz, 1H), 6.70 (dd, $^3J_{H,H}$ = 8.8 Hz, $^4J_{H,H}$ = 2.4 Hz, 1H), 6.56 (m, 1H), 6.12 (d, $^4J_{H,H}$ = 2.4 Hz, 1H), 3.39 (s, 3H). ^{13}C NMR (151 MHz, DMSO-d_6) δ [ppm] = 161.45 (d, $^1J_{C,F}$ = 245.0 Hz, CF_{arom}), 152.8, 146.32 (dd, $^1J_{C,F}$ = 238.7 Hz, $^2J_{C,F}$ = 16.2 Hz, 1C, CF_{arom}), 144.62 (dd, $^1J_{C,F}$ = 233.9 Hz, $^2J_{C,F}$ = 15.0 Hz, 1C, CF_{arom}), 134.7, 132.0, 131.9, 131.2, 131.1, 128.6, 128.97 (q, $^1J_{C,F}$ = 283.8 Hz, 1C, CF_3), 126.8, 126.1, 121.30 (d, $^3J_{C,F}$ = 9.2 Hz, 1C, C_q), 115.1, 114.9, 113.2, 112.4, 111.8, 111.0, 106.96 (d, $^2J_{C,F}$ = 23.6 Hz, CH), 102.8, 99.6, 54.9, 54.41 (q, $^2J_{C,F}$ = 25.8 Hz, 1C, C_q). ^{19}F NMR (565 MHz, DMSO-d_6) δ [ppm] = -63.12 (s, 3F, CF_3), -113.65 – -115.76

(m, 1F, F_{arom}), -144.28 – -146.12 (m, 1F, F_{arom}), -147.57 – -148.49 (m, 1F, F_{arom}). LC-MS: positive [*m*/*z*] = 475.3 ([M+H]$^+$. Purity by HPLC-UV (254 nm) ESI-MS: 97.0%. HRMS (ESI-QTOF) calculated for $C_{26}H_{16}F_6N_2O$ [M + H]+: 475.1245 found: 475.1244.

6-Bromo-3-(1-(4-bromophenyl)-2,2,2-trifluoro-1-(5-methoxy-1*H*-indol-3-yl)ethyl)-1*H*-indole (3s): The compound **3s** was synthesized by the reaction of 2,2,2-trifluoro-1-(4-bromophenyl)-1-(5-methoxy-1*H*-indol-3-yl)ethan-1-ol (**1h**, 80 mg, 0.2 mmol) and 6-bromo-1*H*-indole (**2j**, 40 mg, 0.50 mmol) in acetonitrile (5 mL) in the presence of iodine (5 mg, 10 mol%). The product was isolated as brown solid (104 g, 90%). M.p: 105-107 °C. ^{1}H NMR (600 MHz, DMSO-d_6) δ [ppm] = 11.35 (s, 1H, NH), 11.12 (s, 1H, NH), 7.63 – 7.55 (m, 3H), 7.32 (m, 3H), 7.04 – 6.91 (m, 3H), 6.83 – 6.67 (m, 2H), 6.15 (d, $^4J_{H,H}$ = 2.4 Hz, 1H), 3.40 (s, 3H, CH_3). ^{13}C NMR (151 MHz, DMSO-d_6) δ [ppm] = 152.7, 138.1, 137.7, 131.8, 131.3 (2C, CH), 131.1 (2C, CH), 128.82 (q, $^1J_{C,F}$ = 284.8 Hz, 1C, CF_3), 127.8, 126.8, 126.0, 124.8, 122.5, 121.8, 121.2, 114.4, 114.0, 112.8, 112.4, 111.6, 110.9, 102.8, 54.84 (q, $^2J_{C,F}$ = 27.7 Hz, 1C, C_q), 54.8 (1C, CH_3). ^{19}F NMR (565 MHz, DMSO-d_6) δ [ppm] = -62.83 (s, 3F, CF_3). LC-MS: positive [*m*/*z*] = 579 [M+H]$^+$. Purity by HPLC-UV (254 nm) ESI-MS: 99%. HRMS (ESI-QTOF) calculated for $C_{26}H_{16}F_6N_2O$ [M + H]+: 576.9738 found: 576.9731.

7-Bromo-3-(1-(4-bromophenyl)-2,2,2-trifluoro-1-(5-methoxy-1*H*-indol-3-yl)ethyl)-1*H*-indole (3t): The compound **3t** was synthesized by the reaction of 2,2,2-trifluoro-1-(4-bromophenyl)-1-(5-methoxy-1*H*-indol-3-yl)ethan-1-ol (**1h**, 80 mg, 0.2 mmol) and 7-bromo-1*H*-indole (**2g**, 40 mg, 0.50 mmol) in acetonitrile (5 mL) in the presence of iodine (5 mg, 10 mol%). The product was isolated as brown solid (95 g, 82%). M.p: 96-98 °C. ^{1}H NMR (600 MHz, DMSO-d_6) δ [ppm] = 11.48 (s, 1H, NH), 11.14 (s, 1H, NH), 7.62 – 7.55 (m, 2H), 7.40 – 7.27 (m, 4H), 7.00 – 6.87 (m, 3H), 6.82 – 6.68 (m, 2H), 6.18 (d, $^4J_{H,H}$ = 2.4 Hz, 1H), 3.41 (s, 3H, CH_3). ^{13}C NMR (151 MHz, DMSO-d_6) δ [ppm] = 152.8, 135.1, 131.9, 131.3, 131.1, 130.7, 128.80 (q, $^1J_{C,F}$ = 283.7 Hz, 1C, CF_3), 127.9 (2C, CH), 127.4, 126.9, 126.0, 125.0, 123.9, 121.3,

120.5, 120.4, 114.0, 112.5, 111.7, 111.0, 104.5, 102.8, 54.9 (1C, CH_3), 54.88 (q, $^{2}J_{C,F}$ = 25.6 Hz, 1C, C_q). ^{19}F NMR (565 MHz, DMSO-d_6) δ [ppm] = -62.67 (s, 3F, CF_3). LC-MS: positive [*m/z*] = 579.1 $[M+H]^+$. Purity by HPLC-UV (254 nm) ESI-MS: 99%. HRMS (ESI-QTOF) calculated for $C_{25}H_{17}Br_2F_3N_2O$ $[M + H]^+$: 576.9738 found: 576.9734.

3-(1-(4-Bromophenyl)-2,2,2-trifluoro-1-(5-methoxy-1*H*-indol-3-yl)ethyl)-5,6-difluoro-1*H*-indole (3u): The compound **3u** was synthesized by the reaction of 2,2,2-trifluoro-1-(4-bromophenyl)-1-(5-methoxy-1*H*-indol-3-yl)ethan-1-ol (**1h**, 80 mg, 0.2 mmol) and 5,6-difluoro-1*H*-indole (**2h**, 31 mg, 0.2 mmol) in acetonitrile (5 mL) in the presence of iodine (5 mg, 10 mol%). The product was isolated as light brown solid (99 mg, 92%). M.p: 155-157 °C. ^{1}H NMR (600 MHz, DMSO-d_6) δ [ppm] = 11.42 (s, 1H, NH), 11.15 (s, 1H, NH), 7.61 – 7.58 (m, 2H), 7.43 (dd, $^{3}J_{H,H}$ = 11.0 Hz, $^{3}J_{H,H}$ = 7.1 Hz, 1H), 7.32 (m, 3H), 7.09 (d, $^{4}J_{H,H}$ = 2.6 Hz, 1H), 7.00 (d, $^{4}J_{H,H}$ = 2.6 Hz, 1H), 6.70 (dd, $^{3}J_{H,H}$ = 8.8 Hz, $^{4}J_{H,H}$ = 2.4 Hz, 1H), 6.59 (m, 1H), 6.11 (d, $^{4}J_{H,H}$ = 2.4 Hz, 1H), 3.39 (s, 3H, CH_3). ^{13}C NMR (151 MHz, DMSO-d_6) δ [ppm] = 152.8, 146.31 (dd, $^{1}J_{C,F}$ = 238.8 Hz, $^{2}J_{C,F}$ = 16.1 Hz, 1C, CF_{arom}), 144.61 (dd, $^{1}J_{C,F}$ = 234.0 Hz, $^{2}J_{C,F}$ = 15.1 Hz, 1C, CF_{arom}), 137.9, 132.0, 131.9, 131.8, 131.2, 131.2, 128.7, 127.76 (q, $^{1}J_{C,F}$ = 285.6 Hz, 1C, CF_3), 126.8, 121.3, 121.2, 112.8, 112.4 (2C, CH), 111.3, 111.0, 107.10 – 106.77 (m, 1C, CH), 102.6, 99.7, 99.6, 54.8, 54.77 (q, $^{2}J_{C,F}$ = 26.7 Hz, 1C, C_q). ^{19}F NMR (565 MHz, DMSO-d_6) δ [ppm] = -62.96 (s, 3F, CF_3), -142.56 – -146.59 (m, 1F, F_{arom}), -147.95 (m, 1F, F_{arom}). LC-MS: positive [*m/z*] = 537.0 $[M+H]^+$. Purity by HPLC-UV (254 nm) ESI-MS: 96.0%. HRMS (ESI-QTOF) calculated for $C_{25}H_{16}BrF_5N_2O$ [M + H]+: 535.0444 found: 535.0440.

5-Methoxy-3-(2,2,2-trifluoro-1-(1H-indol-3-yl)-1-(thiophen-2-yl)ethyl)-1H-indole (3v): The compound **3v** was synthesized by the reaction of 2,2,2-trifluoro-1-(5-methoxy-1*H*-indol-3-yl)-1-(thiophen-2-yl)ethan-1-ol (**1i**, 65 mg, 0.2 mmol) and 1*H*-indole (**2a**, 24 mg, 0.2 mmol) in acetonitrile (5 mL) in the presence of iodine (5 mg, 10 mol%). The product was isolated as brown solid (76 mg, 89%). M.p: 105-107 °C. ^{1}H NMR (600 MHz, DMSO-d_6) δ [ppm] = 11.24

(s, 1H, NH), 11.11 (s, 1H, NH), 7.49 – 7.45 (m, 1H), 7.39 (d, $^3J_{H,H}$ = 8.2 Hz, 1H), 7.26 (d, $^3J_{H,H}$ = 8.8 Hz, 1H), 7.23 – 7.16 (m, 3H), 7.06 (m, 1H), 6.99 (ddd, $^3J_{H,H}$ = 8.1 Hz, $^3J_{H,H}$ = 6.9 Hz, $^4J_{H,H}$ = 1.1 Hz, 1H), 6.82 (d, $^3J_{H,H}$ = 8.2 Hz, 1H), 6.71 (ddd, $^3J_{H,H}$ = 8.1 Hz, $^3J_{H,H}$ = 6.9 Hz, $^4J_{H,H}$ = 1.1 Hz, 1H), 6.64 (dd, $^3J_{H,H}$ = 8.8 Hz, $^4J_{H,H}$ = 2.4 Hz, 1H), 6.10 (d, $^4J_{H,H}$ = 2.4 Hz, 1H), 3.33 (s, 3H, CH_3). ^{13}C NMR (151 MHz, DMSO-d_6) δ [ppm] = 152.5, 143.6, 136.6, 131.7, 129.47 (q, $^1J_{C,F}$ = 282.3 Hz, 1C, CF_3), 127.8, 126.4, 126.4, 126.2, 126.0, 125.9, 125.8, 121.0, 120.8, 118.7, 112.8, 112.3, 112.1, 111.7, 110.7, 102.9, 54.8, 52.26 (d, $^2J_{C,F}$ = 27.7 Hz, 1C, C_q). ^{19}F NMR (565 MHz, DMSO-d_6) δ [ppm] = -66.43 (s, 3F, CF_3). LC-MS: positive [m/z] = 427 $[M+H]^+$. Purity by HPLC-UV (254 nm) ESI-MS: 98.0%. HRMS (ESI-QTOF) calculated for $C_{23}H_{17}F_3N_2OS$ [M + H]+: 427.1092 found: 427.1089.

5-Fluoro-3-(2,2,2-trifluoro-1-(5-methoxy-1*H*-indol-3-yl)-1-(thiophen-2-yl)-ethyl)-1*H*-indole (3w): The compound **3v** was synthesized by the reaction of 2,2,2-trifluoro-1-(5-methoxy-1*H*-indol-3-yl)-1-(thiophen-2-yl)ethan-1-ol (**1i**, 65 mg, 0.2 mmol) and 5-fluoro-1*H*-indole (**2d**, 27 mg, 0.2 mmol) in acetonitrile (5 mL) in the presence of iodine (5 mg, 10 mol%). The product was isolated as light brown solid (80 mg, 90%). M.p: 221-223 °C. ^{1}H NMR (600 MHz, DMSO-d_6) δ [ppm] = 11.39 (s, 1H, NH), 11.15 (s, 1H, NH), 7.49 (m, 1H), 7.40 (m, 1H), 7.32 (d, $^4J_{H,H}$ = 2.5 Hz, 1H), 7.28 (d, J = 8.8 Hz, 1H), 7.24 – 7.20 (m, 2H), 7.08 (m, 1H), 6.85 (td, $^3J_{H,H}$ = 8.8 Hz, $^4J_{H,H}$ = 2.5 Hz, 1H), 6.66 (dd, $^3J_{H,H}$ = 8.8 Hz, $^4J_{H,H}$ = 2.5 Hz, 1H), 6.35 (dd, $^3J_{H,H}$ = 11.0 Hz, 2.5 Hz, 1H), 6.07 (d, $^4J_{H,H}$ = 2.5 Hz, 1H), 3.32 (s, 3H, CH_3). ^{13}C NMR (151 MHz, DMSO-d_6) δ [ppm] = 156.25 (d, $^1J_{C,F}$ = 230.7 Hz, 1C, CF_{arom}), 152.6, 143.3, 133.3, 131.6, 129.36 (q, $^1J_{C,F}$ = 279.4 Hz, 1C, CF_3), 127.8, 127.7, 126.6, 126.3, 126.2 (2C, CH), 126.2, 126.0, 112.2, 110.8, 109.5, 109.3, 105.2, 105.0, 102.7, 54.7 (1C, CH_3), 52.03 (q, $^2J_{C,F}$ = 28.4 Hz, 1C, C_q). ^{19}F NMR (565 MHz, DMSO-d_6) δ [ppm] = -66.66 (s, 3F, CF_3), -124.60 – -124.71 (m, 1F, F_{arom}). LC-MS: positive [m/z] = 445.0 $[M+H]^+$. Purity by HPLC-UV (254 nm) ESI-MS: 97.0%. HRMS (ESI-QTOF) calculated for $C_{23}H_{16}F_4N_2OS$ $[M + H]^+$: 445.0998 found: 445.0995.

5,6-Difluoro-3-(2,2,2-trifluoro-1-(5-methoxy-1*H*-indol-3-yl)-1-(thiophen-2-yl)ethyl)-1*H*-indole (3x): The compound **3x** was synthesized by the reaction of 2,2,2-trifluoro-1-(5-methoxy-1*H*-indol-3-yl)-1-(thiophen-2-yl)ethan-1-ol (**1i**, 65 mg, 0.2 mmol) and 5,6-difluoro-1*H*-indole (**2h**, 31 mg, 0.2 mmol) in acetonitrile (5 mL) in the presence of iodine (5 mg, 10 mol%). The product was isolated as white solid (75.8 mg, 82%). M.p: 217-219 °C. ^{1}H NMR (600 MHz, DMSO-d_6) δ [ppm] = 11.46 (s, 1H, NH), 11.18 (s, 1H, NH), 7.49 (m, 1H), 7.41 (m, 1H), 7.37 – 7.32 (m, 1H), 7.29 (d, $^{3}J_{H,H}$ = 8.8 Hz, 1H), 7.26 – 7.21 (m, 2H), 7.09 (m, 1H), 6.67 (dd, $^{3}J_{H,H}$ = 8.9 Hz, $^{3}J_{H,H}$ = 2.4 Hz, 1H), 6.45 (m, 1H), 6.07 – 6.03 (m, 1H), 3.34 (s, 3H, CH_3). ^{13}C NMR (151 MHz, DMSO-d_6) δ [ppm] = 152.6, 146.25 (dd, $^{1}J_{C,F}$ = 238.6 Hz, $^{2}J_{C,F}$ = 16.2 Hz, 1C, CF_{arom}), 144.50 (dd, J = 233.7 Hz, $^{2}J_{C,F}$ = 15.2 Hz, 1C, CF_{arom}), 143.1, 131.7, 131.6, 128.34 (q, J = 279.9 Hz, 1C, CF_3), 127.8, 127.8, 126.6, 126.3, 126.2, 126.0, 121.43 (q, $^{3}J_{C,F}$ = 9.1 Hz, 1C, C_q), 113.05 (d, $^{4}J_{C,F}$ = 5.7 Hz, 1C, C_q), 112.3, 111.7, 110.9, 106.75 (d, $^{2}J_{C,F}$ = 22.4 Hz, 1C, CH_{arom}), 102.6, 99.48 (d, $^{2}J_{C,F}$ = 21.5 Hz, 1C, CH_{arom}), 54.8 (1C, CH_3), 51.94 (q, $^{2}J_{C,F}$ = 29.0 Hz, 1C, C_q). ^{19}F NMR (565 MHz, DMSO-d_6) *δ* [ppm] = -66.82 (s, 3F, CF_3), -145.02 – -145.35 (m, 1F, F_{arom}), -148.02 – -148.44 (m, 1F, F_{arom}). LC-MS: positive [*m/z*] = 463 $[M+H]^+$. Purity by HPLC-UV (254 nm) ESI-MS: 99.0%. HRMS (ESI-QTOF) calculated for $C_{23}H_{15}F_5N_2OS$ $[M + H]^+$: 463.0903 found: 463.0901.

5-Fluoro-3-(2,2,3,3,3-pentafluoro-1-(5-methoxy-1*H*-indol-3-yl)-1-phenyl-propyl)-1*H*-indole (3y): The compound **3y** was synthesized by the reaction of 2,2,2-trifluoro-1-(5-methoxy-1*H*-indol-3-yl)-1-(thiophen-2-yl)ethan-1-ol (**1j**, 75 mg, 0.20 mmol) and 5-fluoro-1*H*-indole (**2d**, 37 mg, 0.20 mmol) in acetonitrile (5 mL) in the presence of iodine (5 mg, 10 mol%). The product was isolated as white solid (0.96g, 96%). M.p: 104.9 °C. ^{1}H NMR (600 MHz, DMSO-d_6) *δ* [ppm] = 11.32 (s, 1H, NH), 11.08 (s, 1H, NH), 7.64 – 7.56 (m, 2H), 7.40 – 7.21 (m, 7H), 6.85 (td, $^{3}J_{H,H}$ = 9.0 Hz, $^{4}J_{H,H}$ = 2.5 Hz, 1H), 6.66 (dd, $^{3}J_{H,H}$ = 8.8 Hz, $^{4}J_{H,H}$ = 2.4 Hz, 1H), 6.54 – 6.46 (m, 1H), 6.28 – 6.24 (m, 1H), 3.39 (s, 3H, CH_3). ^{13}C NMR (151 MHz, DMSO-d_6) *δ*

[ppm] = 156.08 (q, $^{1}J_{C,F}$ = 230.8 Hz, 1C, CF_{arom}), 152.4, 138.0, 133.1, 133.11 – 131.10 (m, 1C, CF_3), 131.6, 129.7, 128.4, 127.7, 127.4, 126.8, 126.6, 126.6, 120.44 (m, 1C, CF_2), 112.7, 112.6, 112.1, 111.7, 110.6, 110.5, 109.3, 109.1, 105.94 (d, $^{2}J_{C,F}$ = 27.8 Hz, 1C, CH_{arom}), 103.7, 54.9 (1C, CH_3), 54.62 (q, $^{2}J_{C,F}$ = 19.8 Hz, 1C, C_q). ^{19}F NMR (565 MHz, DMSO-d_6) δ [ppm] = -75.05 (s, 3F, CF_3), -101.15 – -107.13 (m, 2F, CF_2), -122.08 – -127.98 (m, 1F, F_{arom}). LC-MS: positive [*m/z*] = 489.3 $[M+H]^+$. Purity by HPLC-UV (254 nm) ESI-MS: 99%. HRMS (ESI-QTOF) calculated for $C_{26}H_{18}F_6N_2O$ $[M + H]^+$: 489.1402 found: 489.1407.

6-Bromo-3-(2,2,3,3,3-pentafluoro-1-(5-methoxy-1*H*-indol-3-yl)-1-phenylpropyl)-1*H*-indole (3z): The compound **3z** was synthesized by the reaction of 2,2,3,3,3-pentafluoro-1-(5-methoxy-1*H*-indol-3-yl)-1-phenylpropan-1-ol (**1j**, 75 mg, 0.20 mmol) and 6-bromo-1*H*-indole (**2j**, 40 mg, 0.20 mmol) in acetonitrile (5 mL) in the presence of iodine (5 mg, 10 mol%). The product was isolated as light brown solid (98 mg, 89%). M.p: 94.9 °C. ^{1}H NMR (600 MHz, DMSO-d_6) δ [ppm] = 11.31 (d, $^{4}J_{H,H}$ = 2.8 Hz, 1H, NH), 11.07 (d, $^{4}J_{H,H}$ = 2.9 Hz, 1H, NH), 7.66 – 7.50 (m, 3H), 7.33 – 7.23 (m, 5H), 7.20 (d, $^{4}J_{H,H}$ = 2.9 Hz, 1H), 6.90 – 6.78 (m, 2H), 6.65 (dd, $^{3}J_{H,H}$ = 8.8 Hz, $^{4}J_{H,H}$ = 2.4 Hz, 1H), 6.26 (d, $^{4}J_{H,H}$ = 2.6 Hz, 1H), 3.40 (s, 3H, CH_3).^{13}C NMR (151 MHz, DMSO-d_6) δ [ppm] = 152.4, 138.82 – 137.70 (m, 2C, CF_2), 137.4, 131.5, 131.4, 129.6, 127.7 (2C, CH), 127.4, 127.4, 126.9, 126.6, 125.4, 123.1, 121.4, 120.71 – 119.87 (m, 1C, CF_3), 114.2, 113.7, 112.1, 111.9, 110.8, 110.6, 103.7, 54.9, 54.60 (q, $^{2}J_{C,F}$ = 21.2 Hz, 1C, C_q). ^{19}F NMR (565 MHz, DMSO-d_6) δ [ppm] = -75.11 (s, 3F, CF_3), -100.18 – -107.51 (m, 2F, CF_2). LC-MS: positive [*m/z*] = 551.1 ($[M+H]^+$). Purity by HPLC-UV (254 nm) ESI-MS: 99%. HRMS (ESI-QTOF) calculated for $C_{26}H_{18}BrF_5N_2O$ $[M + H]^+$: 549.0601 found: 549.0600

6-Fluoro-3-(2,2,3,3,3-pentafluoro-1-(5-methoxy-1*H*-indol-3-yl)-1-phenyl-propyl)-1*H*-indole (3aa): The compound **3aa** was synthesized by the reaction of 2,2,3,3,3-pentafluoro-1-(5-methoxy-1*H*-indol-3-yl)-1-phenylpropan-1-ol (**1j**, 75 mg, 0.20 mmol) and 6-fluoro-1*H*-indole (**2e**, 27 mg, 0.20 mmol) in acetonitrile (5 mL) in the presence of iodine (5 mg, 10 mol%). The product was isolated as brown solid (88 mg, 90%). M.p: 203.5 °C. ^{1}H NMR (600 MHz, DMSO-d_6) δ [ppm] = 11.24 (s, 1H, NH), 11.07 (s,1H, NH), 7.60 (d, $^{3}J_{H,H}$ = 7.8 Hz, 2H), 7.34 – 7.12 (m, 7H), 6.86 (dd, $^{3}J_{H,H}$ = 9.0 Hz, $^{4}J_{H,H}$ = 5.5 Hz, 1H), 6.69 – 6.56 (m, 2H), 6.28 (d, $^{4}J_{H,H}$ = 2.5 Hz, 1H), 3.40 (s, 3H). ^{13}C NMR (151 MHz, DMSO-d_6) δ [ppm] = 158.31 (d, $^{1}J_{C,F}$ = 235.3 Hz, 1C, CF_{arom}), 152.4, 136.4, 136.3, 131.5, 129.7, 127.6 (2C, CH), 127.4, 127.1, 126.9, 126.7, 123.1, 122.41 (d, $^{3}J_{C,F}$ = 13.1 Hz, 1C_q, C_{indole}),.120.74 – 119.63 (m, 1C, CF_3), 118.82 – 117.84 (m, 1C, CF_2), 112.1, 111.8, 110.8, 110.6, 107.2, 107.0, 103.8, 97.5, 97.3, 54.9 (1C, CH_3), 54.70 (d, $^{2}J_{C,F}$ = 20.8 Hz, 1C, C_q). ^{19}F NMR (565 MHz, DMSO-d_6) δ [ppm] = -75.10 (s, 3F, CF_3), -99.88 – -107.13 (m, 2F, CF_2), -119.76 – -124.70 (m, 1F, F_{arom}). LC-MS: positive [*m*/*z*] = 489.3 $[M+H]^+$. Purity by HPLC-UV (254 nm) ESI-MS: 96%. HRMS (ESI-QTOF) calculated for $C_{26}H_{18}F_6N_2O$ $[M + H]^+$: 489.1402 found: 489.1403.

4,5-Difluoro-3-(2,2,3,3,3-pentafluoro-1-(5-methoxy-1*H*-indol-3-yl)-1-phen-ylpropyl)-1*H*-indole (3ab): The compound **3ab** was synthesized by the reaction of 2,2,3,3,3-pentafluoro-1-(5-methoxy-1*H*-indol-3-yl)-1-phenylpropan-1-ol (**1j**, 75 mg, 0.40 mmol) and 4,5-difluoro-1*H*-indole (**2k**, 30 mg, 0.20 mmol) in acetonitrile (5 mL) in the presence of iodine (5 mg, 10 mol%). The product was isolated as colorless solid (87 mg, 86%). M.p: 94.1 °C. ^{1}H NMR (600 MHz, DMSO-d_6) δ [ppm] = 11.38 (d, $^{4}J_{H,H}$ = 3.0 Hz, 1H, NH), 11.11 (d, $^{4}J_{H,H}$ = 2.9 Hz, 1H, NH), 7.59 (d, $^{3}J_{H,H}$ = 7.8 Hz, 2H), 7.43 – 7.21 (m, 8H), 6.69 – 6.60 (m, 2H), 6.28 – 6.21 (m, 1H, OH), 3.40 (s, 3H, CH_3). ^{13}C NMR (151 MHz, DMSO-d_6) δ [ppm] = 152.4, 146.16 (dd, $^{1}J_{C,F}$ = 238.5, 16.4 Hz, 1C, CF_{arom}), 144.29 (dd, $^{1}J_{C,F}$ = 233.6, 15.2 Hz, 1C, CF_{arom}), 137.8, 131.6, 131.5, 129.7, 128.4, 127.8, 127.5, 126.9, 126.6, 121.8, 121.8, 120.70 – 117.66 (m, 2C, CF_2CF_3), 112.2, 112.0,

110.7, 110.6, 107.47 (d, $^2J_{C,F}$ = 22.1 Hz, 1C, CH_{arom}), 103.6, 99.5, 99.4, 54.9 (1C, CH_3), 54.63 (q, $^2J_{C,F}$ = 20.5 Hz, 1C, C_q). ^{19}F NMR (565 MHz, DMSO-d_6) δ [ppm] = -75.06 (s, 3F, CF_3), -104.31 (m, 2F, CF_2), -142.56 – -150.18 (m, 2F, F_{arom}). LC-MS: positive [*m*/*z*] = 507.4 ([M+H]$^+$), 354.1 ([M-4,5-difluoro-1*H*-indole+H]$^+$) 360.1 ([M-5-methoxy-1*H*-indole+H]$^+$). Purity by HPLC-UV (254 nm) ESI-MS:97%. HRMS (ESI-QTOF) calculated for $C_{26}H_{17}F_7N_2O$ [M + H]$^+$: 507.1307 found: 507.1301.

5,6-Difluoro-3-(2,2,3,3,3-pentafluoro-1-(5-methoxy-1*H*-indol-3-yl)-1-phen-ylpropyl)-1*H*-indole (3ac): The compound **3ac** was synthesized by the reaction of 2,2,3,3,3-pentafluoro-1-(5-methoxy-1*H*-indol-3-yl)-1-phenylpropan-1-ol (**1j**, 75 mg, 0.20 mmol) and 5,6-difluoro-1*H*-indole (**1n**, 32 mg, 0.20 mmol) in acetonitrile (5 mL) in the presence of iodine (5 mg, 10 mol%). The product was isolated as colorless solid (93 mg, 92%). M.p: 91.7 °C. ^{1}H NMR (600 MHz, DMSO-d_6) δ [ppm] = 11.38 (d, $^4J_{H,H}$ = 2.7 Hz, 1H, NH), 11.10 (d, $^4J_{H,H}$ = 2.9 Hz, 1H, NH), 7.60 – 7.55 (m, 2H), 7.43 – 7.20 (m, 7H), 6.68 – 6.58 (m, 2H), 6.24 (d, $^4J_{H,H}$ = 2.5 Hz, 1H), 3.39 (s, 3H, CH_3). ^{13}C NMR (151 MHz, DMSO-d_6) δ [ppm] = 152.4, 146.14 (dd, $^1J_{C,F}$ = 238.6 Hz, $^2J_{C,F}$ = 16.1 Hz, 1C, CF_{arom}), 144.27 (dd, $^1J_{C,F}$ = 233.9 Hz, $^1J_{C,F}$ = 14.9 Hz, 1C, CF_{arom}), 138.00 – 137.65 (m, 1C, CF_3), 131.6, 131.6, 131.5, 129.6, 128.4, 127.8 (2C, CH_{arom}), 127.6, 126.9, 126.6, 121.8, 121.8, 120.48 – 119.43 (m, 1C, CF_2), 112.0, 110.5, 107.5, 107.3, 103.6, 99.5, 99.4, 54.9, 54.68 (q, $^2J_{C,F}$ = 21.6 Hz, 1C, C_q). ^{19}F NMR (565 MHz, DMSO-d_6) δ [ppm] = -75.05 (s, 3F, CF_3), -100.18 – -110.42 (m, 2F, CF_2), -141.96 – -153.54 (m, 2F, F_{arom}). LC-MS: positive [*m*/*z*] = 507.4 ([M+H]$^+$), 354.1 ([M-5,6-difluoro-1*H*-indole+H]$^+$) 360.0 ([M-5-methoxy-1*H*-indole+H]$^+$). Purity by HPLC-UV (254 nm) ESI-MS: 99%. HRMS (ESI-QTOF) calculated for $C_{26}H_{17}F_7N_2O$ [M + H]$^+$: 507.1307 found: 507.1303.

5-Methoxy-3-(2,2,3,3,3-pentafluoro-1-(1*H*-indol-3-yl)-1-phenylpropyl)-1*H*-indole (3ad): The compound **3ad** was synthesized by the reaction of 2,2,3,3,3-pentafluoro-1-(5-methoxy-1*H*-indol-3-yl)-1-phenylpropan-1-ol (**1j**, 75 g, 0.20 mmol) and 1*H*-indole (**2a**, 23 mg, 0.20 mmol) in acetonitrile (5 mL) in the presence of iodine (5 mg, 10 mol%). The product was isolated as brown solid (88 mg, 94%). M.p: 118.1 °C. ^{1}H NMR (600 MHz, DMSO-d_6) δ [ppm] = 11.19 (s, 1H, NH), 11.04 (s, 1H, NH), 7.64 – 7.59 (m, 2H, H_{arom}), 7.36 (dd, $^3J_{H,H}$ = 8.1 Hz, $^4J_{H,H}$ = 1.1 Hz, 1H, H_{arom}), 7.32 – 7.23 (m, 4H, H_{arom}), 7.20 (dd, $^3J_{H,H}$ = 10.8 Hz, $^2J_{H,H}$ = 2.7 Hz, 2H), 7.02 – 6.91 (m, 2H), 6.73 (ddd, $^3J_{H,H}$ = 8.1 Hz, $^2J_{H,H}$ = 6.9 Hz, $^4J_{H,H}$ = 1.1 Hz, 1H), 6.65 (dd, $^3J_{H,H}$ = 8.8 Hz, $^3J_{H,H}$ = 2.4 Hz, 1H), 6.32 – 6.27 (m, 1H), 3.39 (s, 3H, CH_3). ^{13}C NMR (151 MHz, DMSO-d_6) δ [ppm] = 152.3, 139.2 (m, 1C, CF_3), 136.4, 131.5, 129.7, 128,2 (m, 1C, CF_2), 127.5 (2C, CH), 127.2 (3C, CH), 126.9, 126.7, 126.4, 126.3, 121.5, 120.8, 118.5, 112.0, 111.6, 111.5, 111.1, 110.5, 103.9, 54.9 (1C, CH_3), 54.83 (m, C_q). ^{19}F NMR (565 MHz, DMSO-d_6) *δ* [ppm] = -75.08 (s, 3F, CF_3), -103.08 – -104.97 (m, 2F, CF_2). LC-MS: positive [*m/z*] = 471.2 ([M+H]$^+$), 354.0([M-1*H*-indole+H]$^+$), 324.1 ([M-5-methoxy-1*H*-indole+H]$^+$). Purity by HPLC-UV (254 nm) ESI-MS: 98%. HRMS (ESI-QTOF) calculated for $C_{26}H_{19}F_5N_2O$ [M + H]$^+$: 471.1496 found: 471.1491.

3-(2,2,3,3,4,4,4-Heptafluoro-1-(1*H*-indol-3-yl)-1-phenylbutyl)-5-methoxy-*1H*-indole (3ae): The compound **3ae** was synthesized by the reaction of 2,2,3,3,4,4,4-heptafluoro-1-(4-fluorophenyl)-1-(6-methoxy-1*H*-indol-3-yl)-butan-1-ol (**1l**, 84 mg, 0.20 mmol) and 1*H*-indole (**2a**, 23 mg, 0.20 mmol) in acetonitrile (5 mL) in the presence of iodine (5 mg, 10 mol%). The product was isolated as light yellow solid (68 mg, 65%). M.p: 107.6 °C. ^{1}H NMR (600 MHz, DMSO-d_6) *δ* [ppm] = 11.19 (d, $^4J_{H,H}$ = 2.7 Hz, 1H), 11.05 (d, $^4J_{H,H}$ = 2.8 Hz, 1H), 7.60 (d, $^3J_{H,H}$ = 7.7 Hz, 2H), 7.37 (d, $^3J_{H,H}$ = 8.1 Hz, 1H), 7.34 – 7.22 (m, 4H), 7.21 – 7.13 (m, 2H), 6.99 (t, $^3J_{H,H}$ = 7.5 Hz, 1H), 6.91 (d, $^3J_{H,H}$ = 8.2 Hz, 1H), 6.73 (t, $^3J_{H,H}$ = 7.6 Hz, 1H), 6.65 (dd, $^4J_{H,H}$ = 8.8 Hz, $^4J_{H,H}$ = 2.4 Hz, 1H), 6.25 (d, $^4J_{H,H}$ = 2.7 Hz, 1H), 3.38 (s, 3H, CH_3). ^{13}C NMR (151

MHz, DMSO-d_6) δ [ppm] = 152.3, 136.4, 131.5, 129.9 (2C, CH), 127.5, 127.3 (2C, CH), 126.8 (2C, CH), 126.4, 126.4, 121.4, 121.16 (m, 1C, CF_3), 120.8, 119.30 (m, 1C, CF_2), 118.69 (m, 1C, CF_2), 118.5, 112.1, 111.6, 111.5, 111.0, 110.5, 103.8, 55.80 (q, $^2J_{C,F}$ = 22.1 Hz, 1C, C_q), 54.9. ^{19}F NMR (565 MHz, DMSO-d_6) δ [ppm] = -75.37 – -87.25 (m, 3F, CF_3), -97.19 – -105.79 (m, 2F, CF_2), -118.50 (m, 2F, CF_2). LC-MS: positive [m/z] = 521.2 ([M+H]$^+$). Purity by HPLC-UV (254 nm) ESI-MS: 97%. HRMS (ESI-QTOF) calculated for $C_{27}H_{19}F_7N_2O$ [M + H]$^+$: 521.1464 found: 521.1460.

5-Fluoro-3-(2,2,3,3,4,4,4-Heptafluoro-1-(5-methoxy-1*H*-indol-3-yl)-1-phenylbutyl)-1*H*-indole (3af): The compound **3af** was synthesized by the reaction of 2,2,3,3,4,4,4-heptafluoro-1-(4-fluorophenyl)-1-(6-methoxy-1*H*-indol-3-yl)-butan-1-ol (**1l**, 84 mg, 0.20 mmol) and 5-fluoro-1*H*-indole (**1d**, 27 mg, 0.20 mmol) in acetonitrile (5 mL) in the presence of iodine (5 mg, 10 mol%). The product was isolated as colorless solid (77 mg, 72%). M.p: 90.9 °C. ^{1}H NMR (600 MHz, DMSO-d_6) δ [ppm] = 11.33 (s, 1H, NH), 11.09 (s, 1H, NH), 7.57 (d, $^3J_{H,H}$ = 7.7 Hz, 2H), 7.38 (dd, $^3J_{H,H}$ = 8.9 Hz, $^4J_{H,H}$ = 4.8 Hz, 1H), 7.35 – 7.25 (m, 5H), 7.19 (s, 1H), 6.84 (td, $^3J_{H,H}$ = 9.0 Hz, $^3J_{H,H}$ = 2.5 Hz, 1H), 6.65 (dd, $^3J_{H,H}$ = 8.8 Hz, $^3J_{H,H}$ = 2.4 Hz, 1H), 6.53 – 6.42 (m, 1H), 6.22 (d, $^3J_{H,H}$ = 15.2 Hz, 1H), 3.38 (s, 3H, CH_3). ^{13}C NMR (151 MHz, DMSO-d_6) δ [ppm] = 156.29 (d, $^1J_{C,F}$ = 230.8 Hz, 1C, CF_{arom}), 152.6, 133.3, 131.7, 130.0, 128.6, 127.8 (2C, CH), 127.7, 126.9, 126.8, 121.25 (m, 2C, CF_2), 119.49 (1C, CF_3), 112.9, 112.8, 112.3, 111.8, 110.9, 110.8, 110.7, 109.5, 109.3, 106.0, 105.9, 103.8, 55.83 (q, $^2J_{C,F}$ = 21.6 Hz, 1C, C_q), 55.0 (1C, CH_3). ^{19}F NMR (565 MHz, DMSO-d_6) δ [ppm] = -80.19 – -80.49 (m, 3F, CF_3), -100.11 – -101.48 (m, 4F, CF_2), -124.56 – -124.76 (m, 1F, F_{arom}). LC-MS: positive [m/z] = 539.4 ([M+H]$^+$). M.p: 90.9 °C. Purity by HPLC-UV (254 nm) ESI-MS: 97%. HRMS (ESI-QTOF) calculated for $C_{27}H_{18}F_8N_2O$ [M + H]$^+$: 539.1370 found: 539.1365.

6-Fluoro-3-(2,2,3,3,4,4,4-heptafluoro-1-(5-methoxy-1*H*-indol-3-yl)-1-phen-ylbutyl)-1*H*-indole (3ag): The compound **3ag** was synthetized by the reaction of 2,2,3,3,4,4,4-heptafluoro-1-(4-fluorophenyl)-1-(6-methoxy-1*H*-indol-3-yl)-butan-1-ol (**1l**, 84 mg, 0.20 mmol)) and 6-fluoro-1*H*-indole (**1e**, 27 mg, 0.20 mmol) in acetonitrile (5 mL) in the presence of iodine (5 mg, 10 mol%). The product was isolated as colorless solid (77 mg, 70%). M.p: 124.1 °C. ^{1}H NMR (600 MHz, DMSO-d_6) δ [ppm] = 11.24 (s, 1H, NH), 11.07 (s, 1H, NH), 7.57 (d, $^{3}J_{H,H}$ = 7.7 Hz, 2H), 7.36 – 7.10 (m, 7H), 6.89 – 6.79 (m, 1H), 6.69 – 6.56 (m, 2H), 6.22 (m, 1H), 3.38 (s, 3H, CH_3). ^{13}C NMR (151 MHz, DMSO-d_6) δ [ppm] = 158.31 (q, $^{1}J_{C,F}$ = 235.3 Hz, 1C, CF_{arom}), 152.4, 136.4, 136.3, 131.5, 129.8, 127.6 (2C, CH), 127.4, 127.1, 126.8, 126.8, 123.2, 122.33 (q, $^{3}J_{C,F}$ = 13.7 Hz, 1C, C_q), 119.29 (m, 1C, CF_3), 114.49 (m, 2C, CF_2), 112.1, 111.8, 110.8, 110.5, 107.2, 107.1, 103.7, 97.5, 97.3, 55.71 (q, $^{2}J_{C,F}$ = 21.9 Hz, 1C, C_q), 54.9 (1C, CH_3). ^{19}F NMR (565 MHz, DMSO-d_6) δ [ppm] = -80.28 – -80.42 (m, 3F, CF_3), -100.15 – -101.40 (m, 4F, CF_2), -122.36 (m, 1F, F_{arom}). LC-MS: positive [*m*/*z*] = 539.5 ($[M+H]^+$). Purity by HPLC-UV (254 nm) ESI-MS: 99%. HRMS (ESI-QTOF) calculated for $C_{27}H_{18}F_8N_2O$ $[M + H]^+$: 539.1370 found: 539.1367.

Reaction of 1l with 2c or 2d

To the solution of 1-(1H-indol-3-yl)-1-phenylethan-1-ol (**1l**, 0.2 mmol) and appropriate indole derivative (**2c or 2d**, 0.2 mmol) in acetonitrile (5 mL), I_2 (0.1 eq., 10%) was added at rt. The mixture was heated to 40 °C for 12 h. The reaction was monitored by TLC with UV detection. The reaction was not initiated even after 12 h.

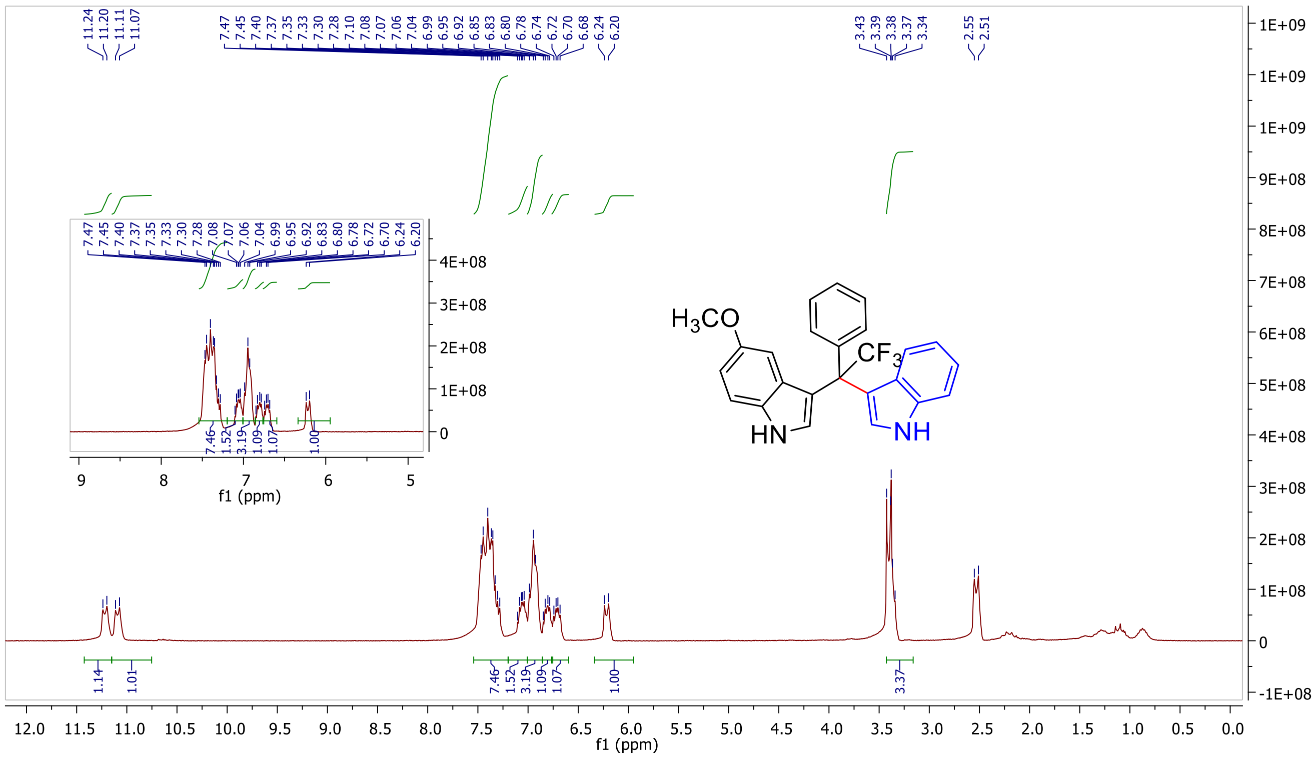

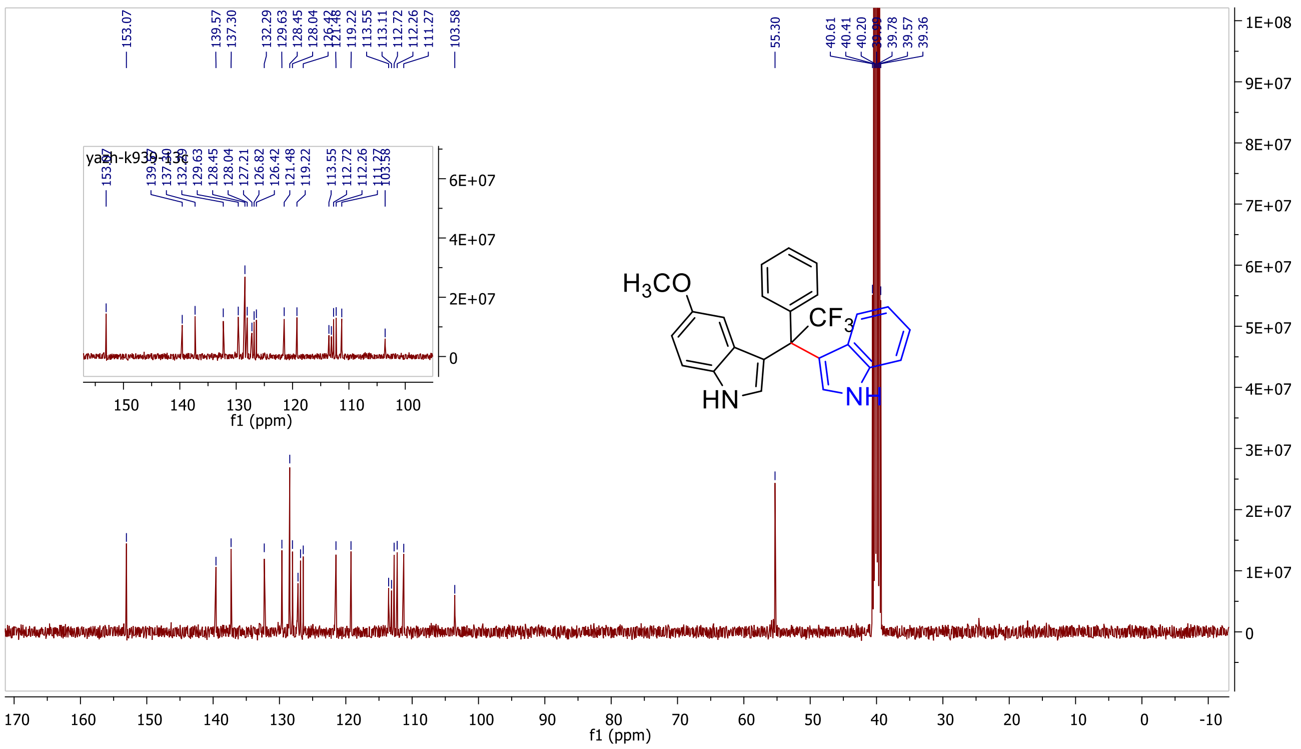

Figure S4. ^{1}H (400 MHz) and ^{13}C (151 MHz) Spectra of 5-methoxy-3-(2,2,2-trifluoro-1-(1*H*-indol-3-yl)-1-phenylethyl)-1*H*-indole (**3a**)

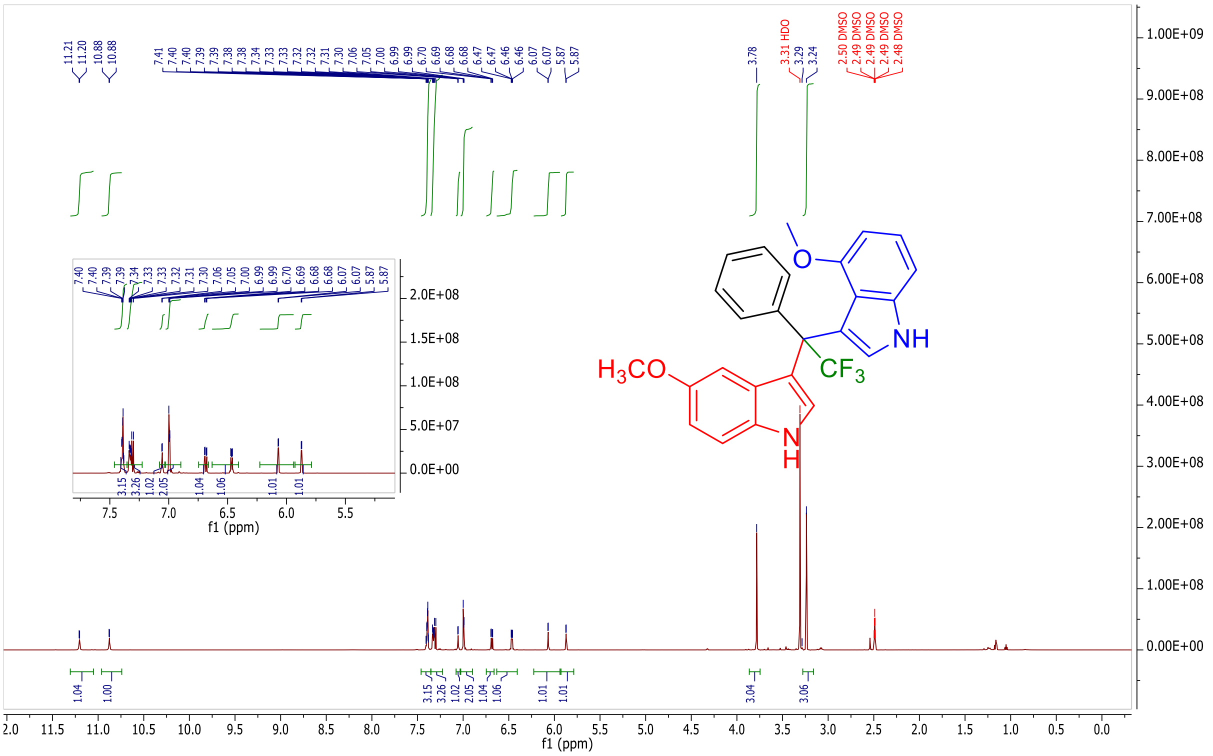

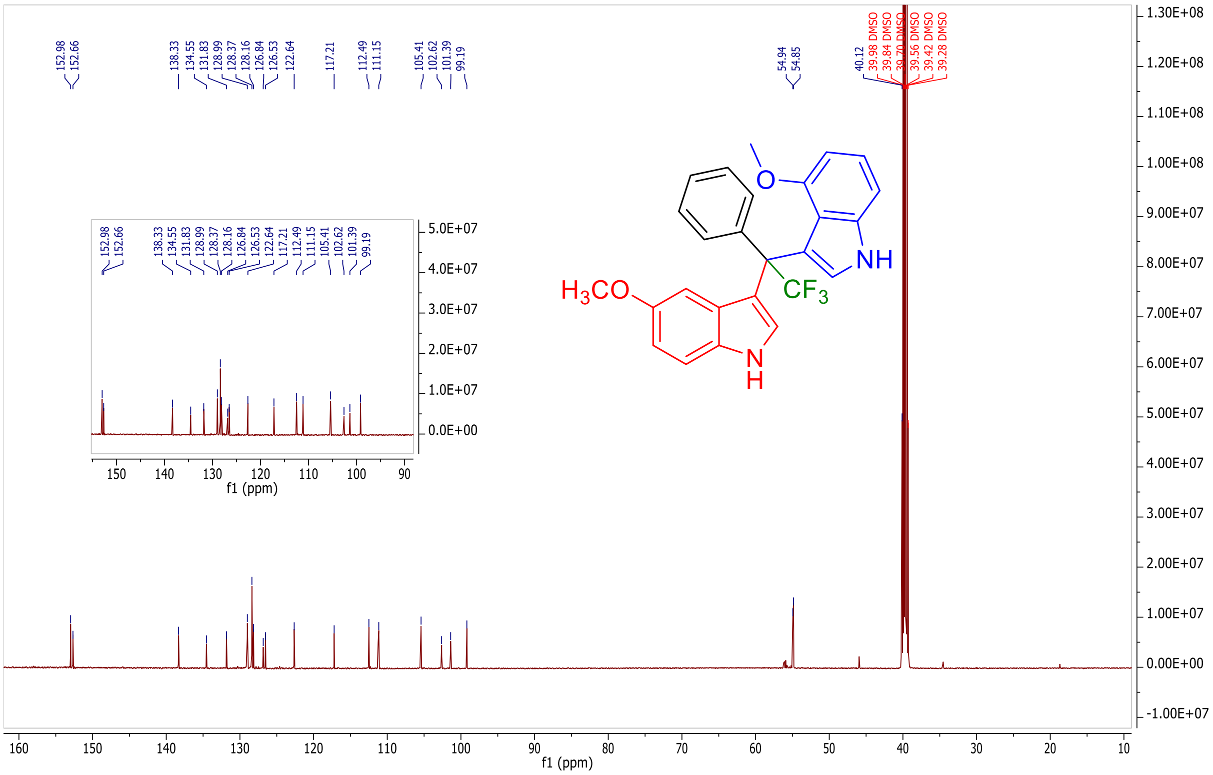

Figure S5. ^{1}H (600 MHz) and ^{13}C (151 MHz) Spectra of 5-methoxy-3-(2,2,2-trifluoro-1-(4-methoxy-1*H*-indol-3-yl)-1-phenylethyl)-1*H*-indole (**3b**)

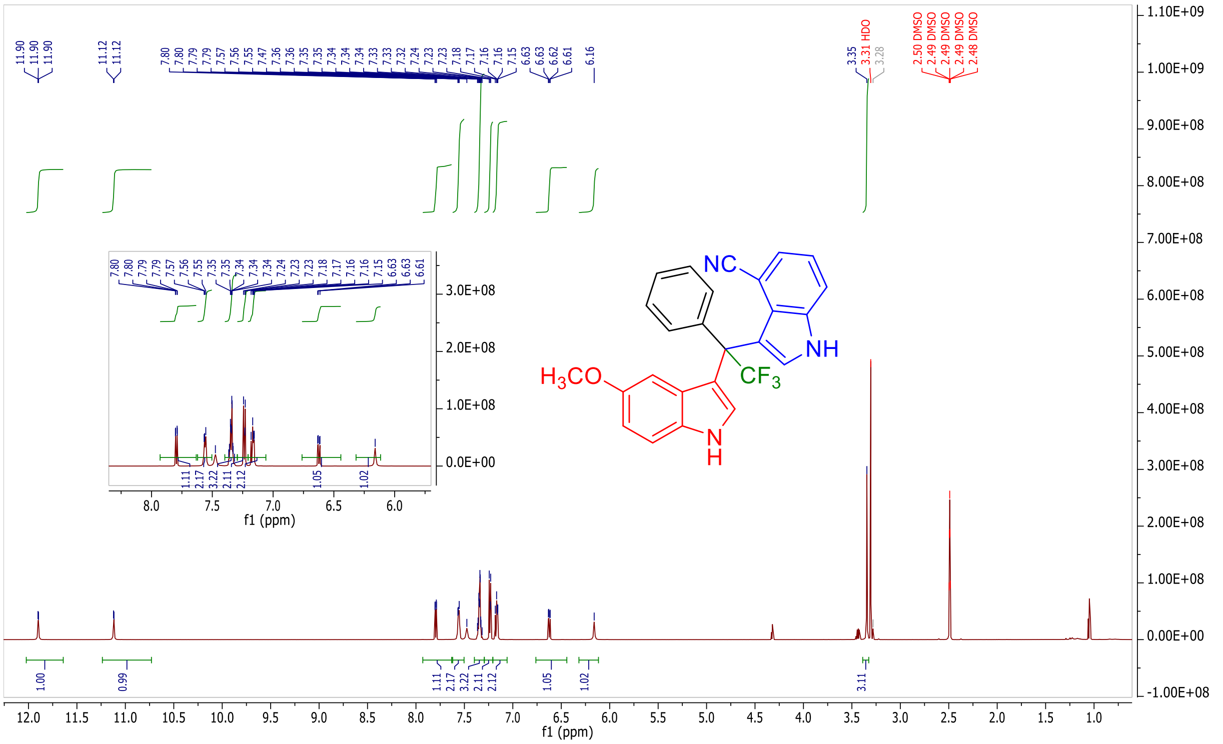

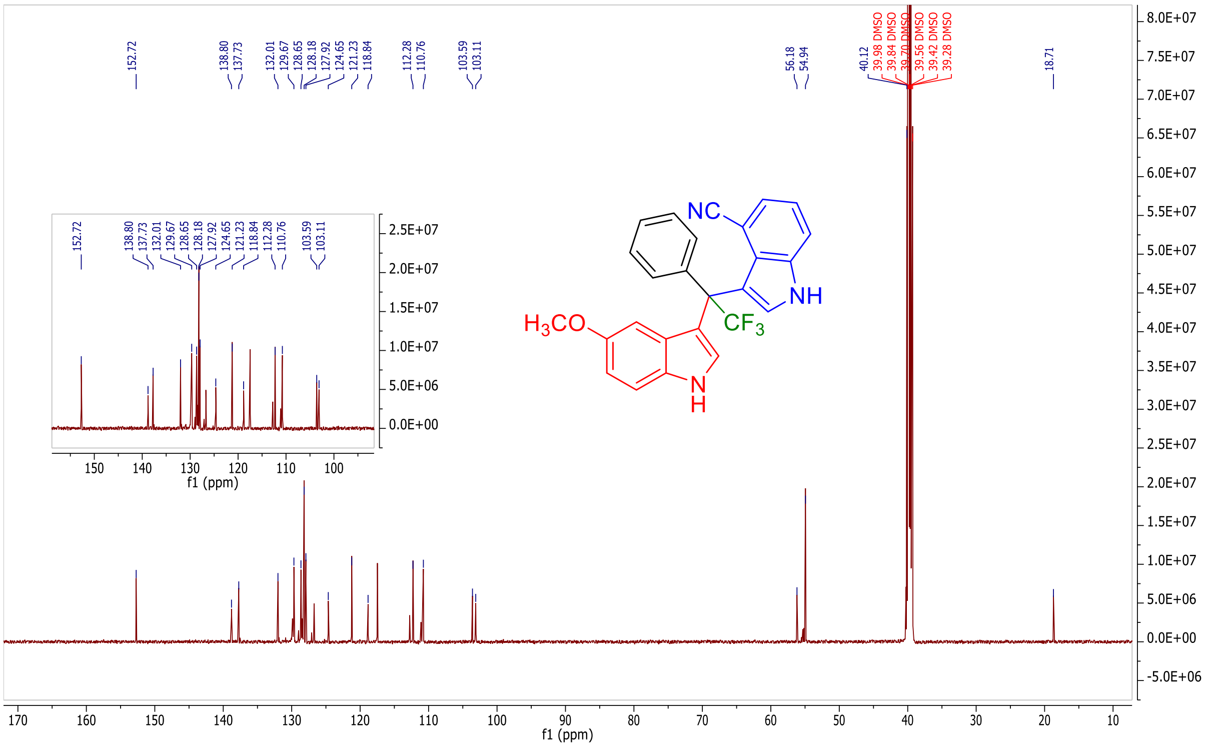

Figure S6. ^{1}H (600 MHz) and ^{13}C (151 MHz) Spectra of 3-(2,2,2-trifluoro-1-(5-methoxy-1*H*-indol-3-yl)-1-phenylethyl)-1*H*-indole-4-carbonitrile (**3c**)

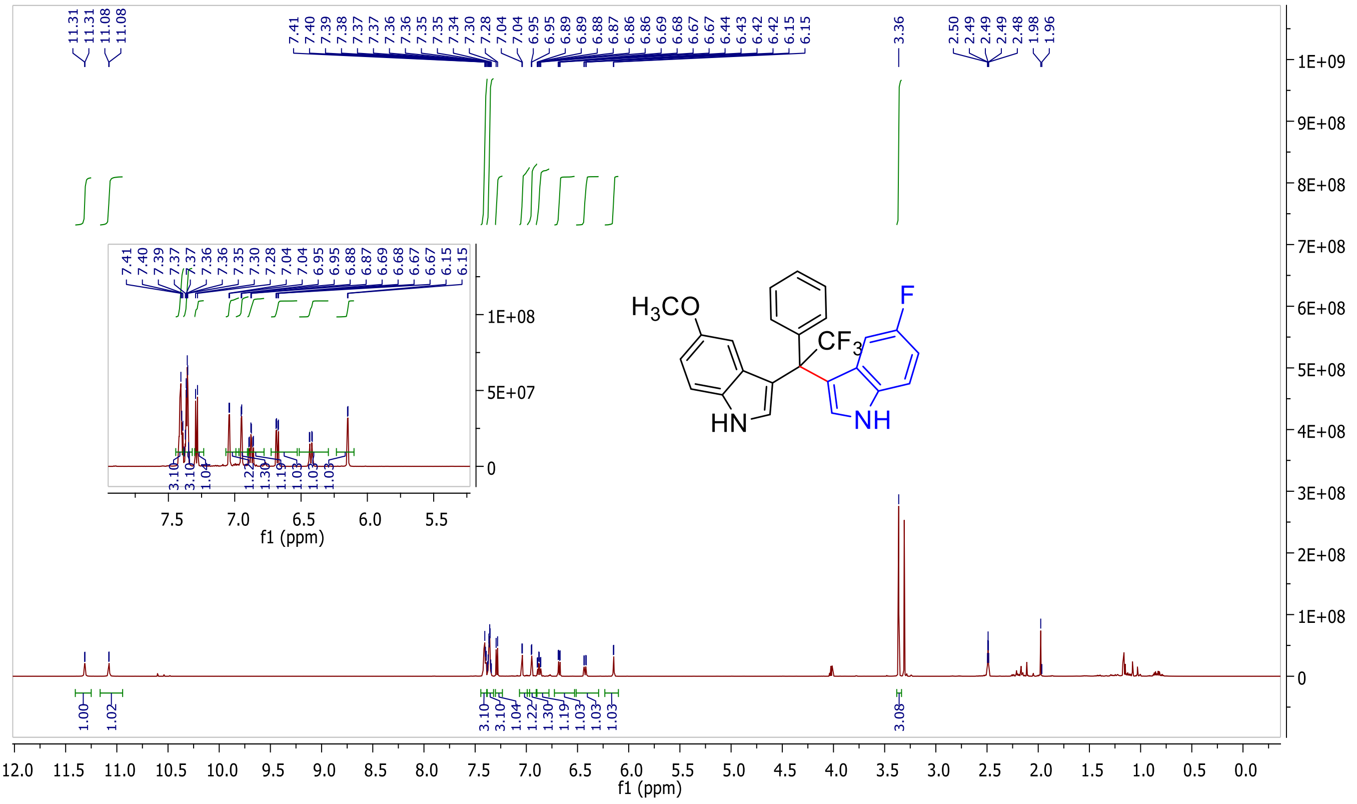

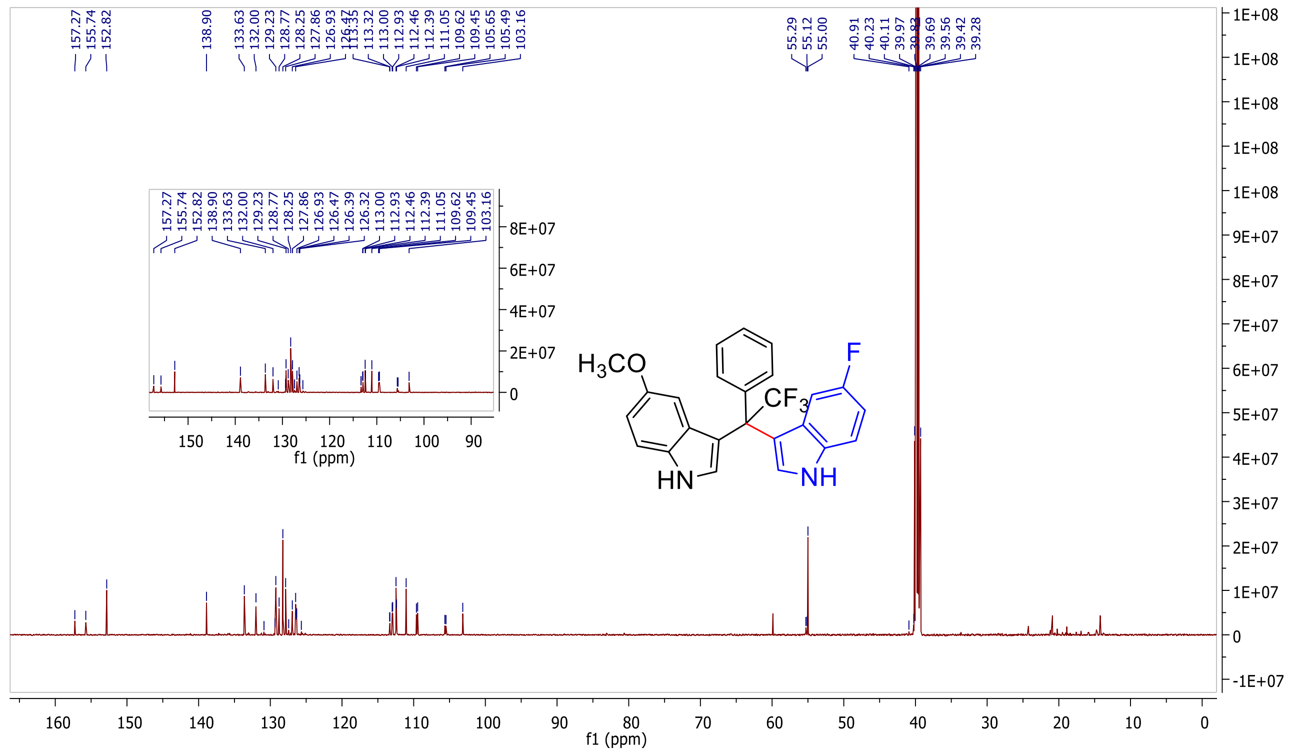

Figure S7. ^{1}H (600 MHz) and ^{13}C (151 MHz) Spectra of 5-fluoro-3-(2,2,2-trifluoro-1-(5-methoxy-1*H*-indol-3-yl)-1-phenylethyl)-1*H*-indole (**3d**)

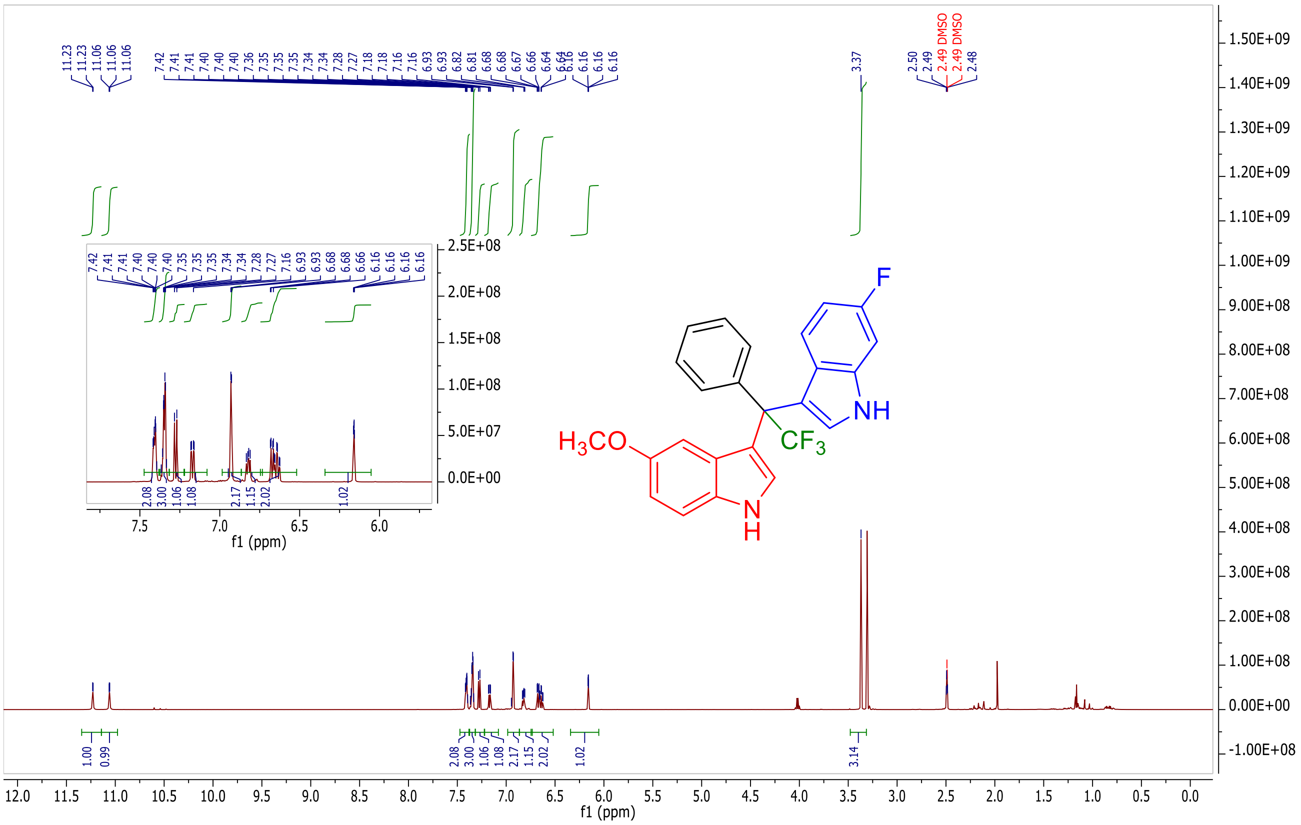

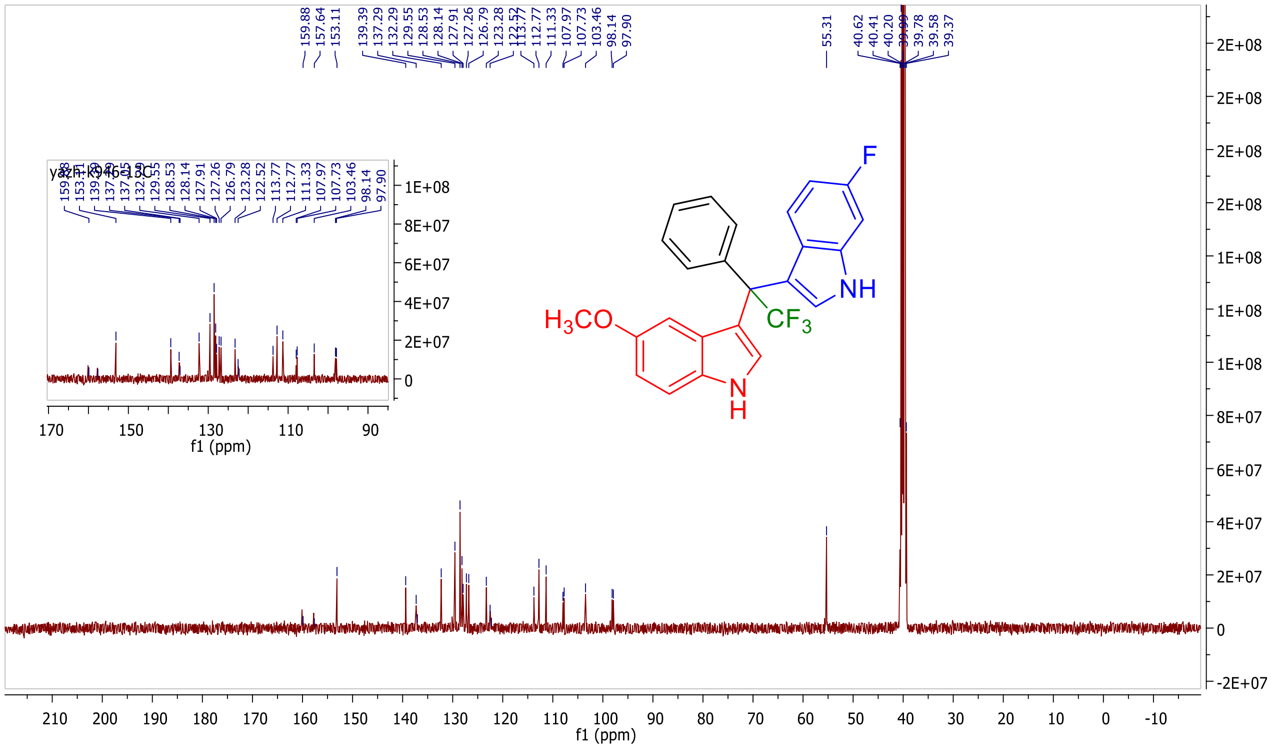

Figure S8. ^{1}H (600 MHz) and ^{13}C (151 MHz) Spectra of 6-fluoro-3-(2,2,2-trifluoro-1-(5-methoxy-1H-indol-3-yl)-1-phenylethyl)-1H-indole (**3e**)

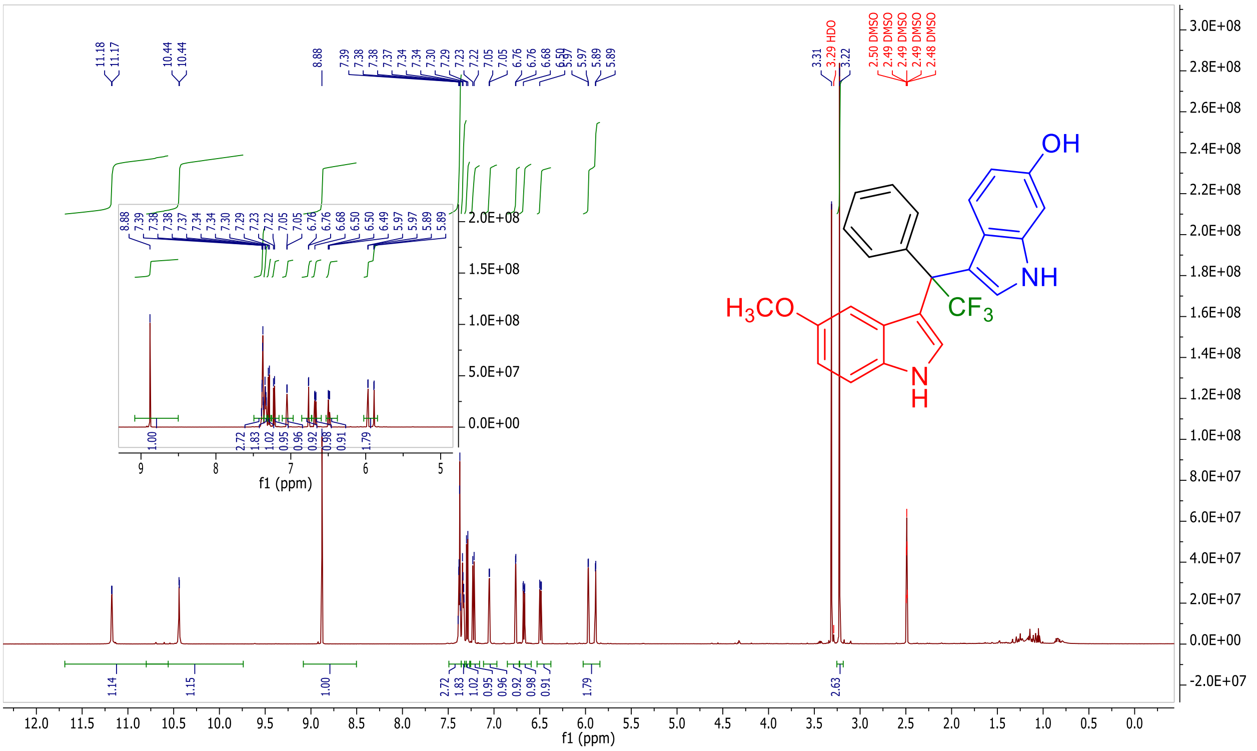

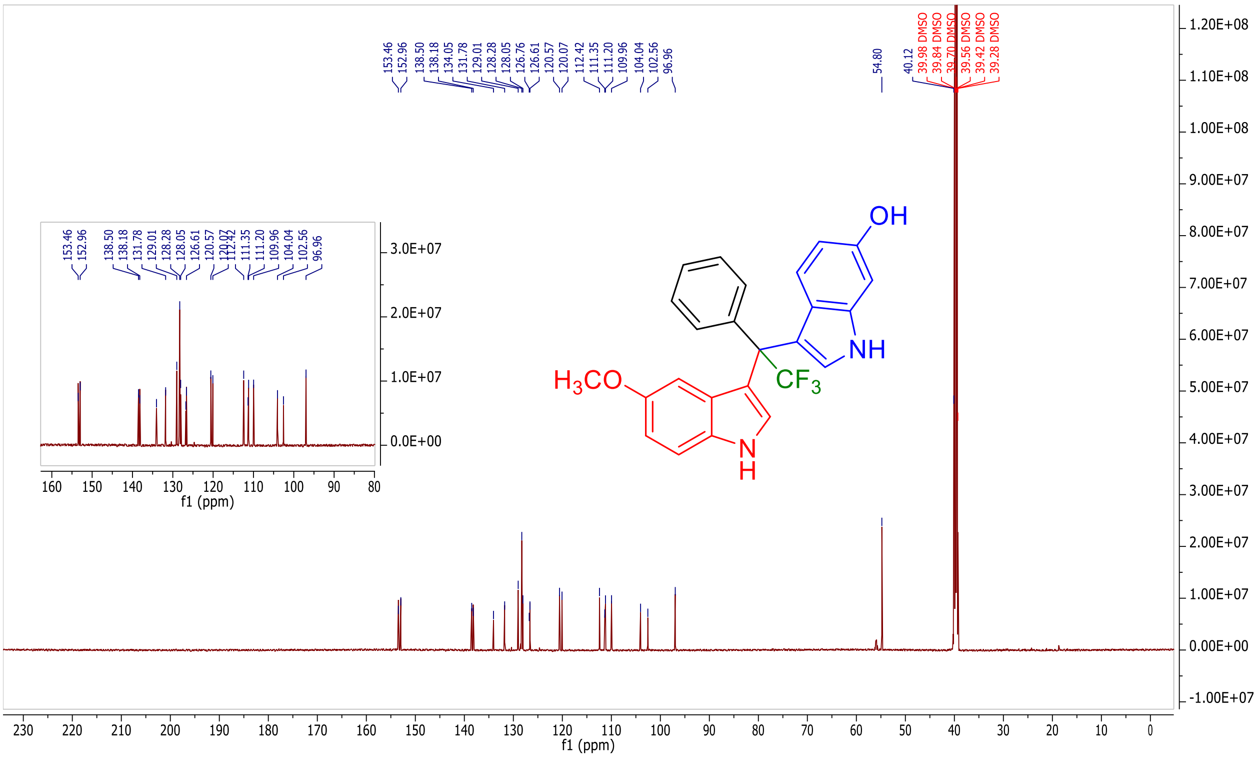

Figure S9. ^{1}H (600 MHz) and ^{13}C (151 MHz) Spectra of 3-(2,2,2-trifluoro-1-(5-methoxy-1*H*-indol-3-yl)-1-phenylethyl)-1*H*-indol-6-ol (**3f**)

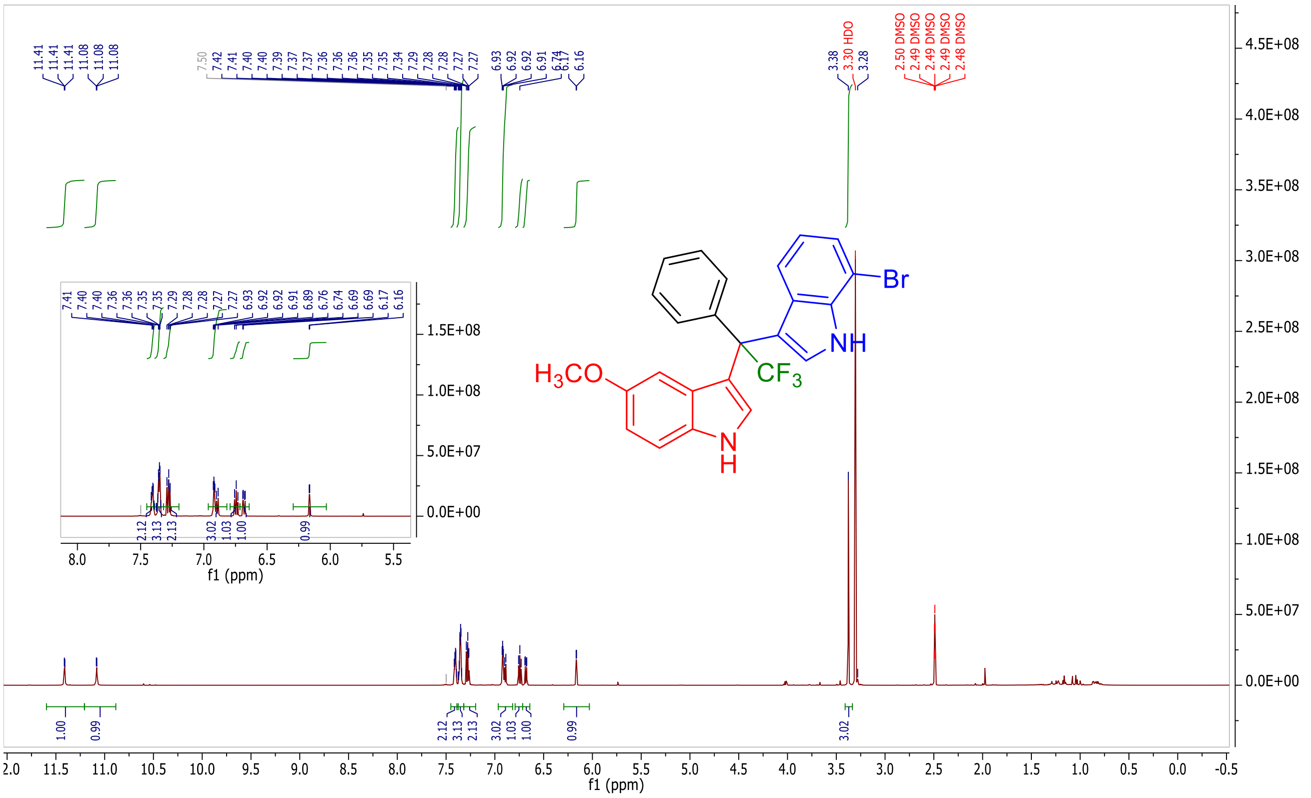

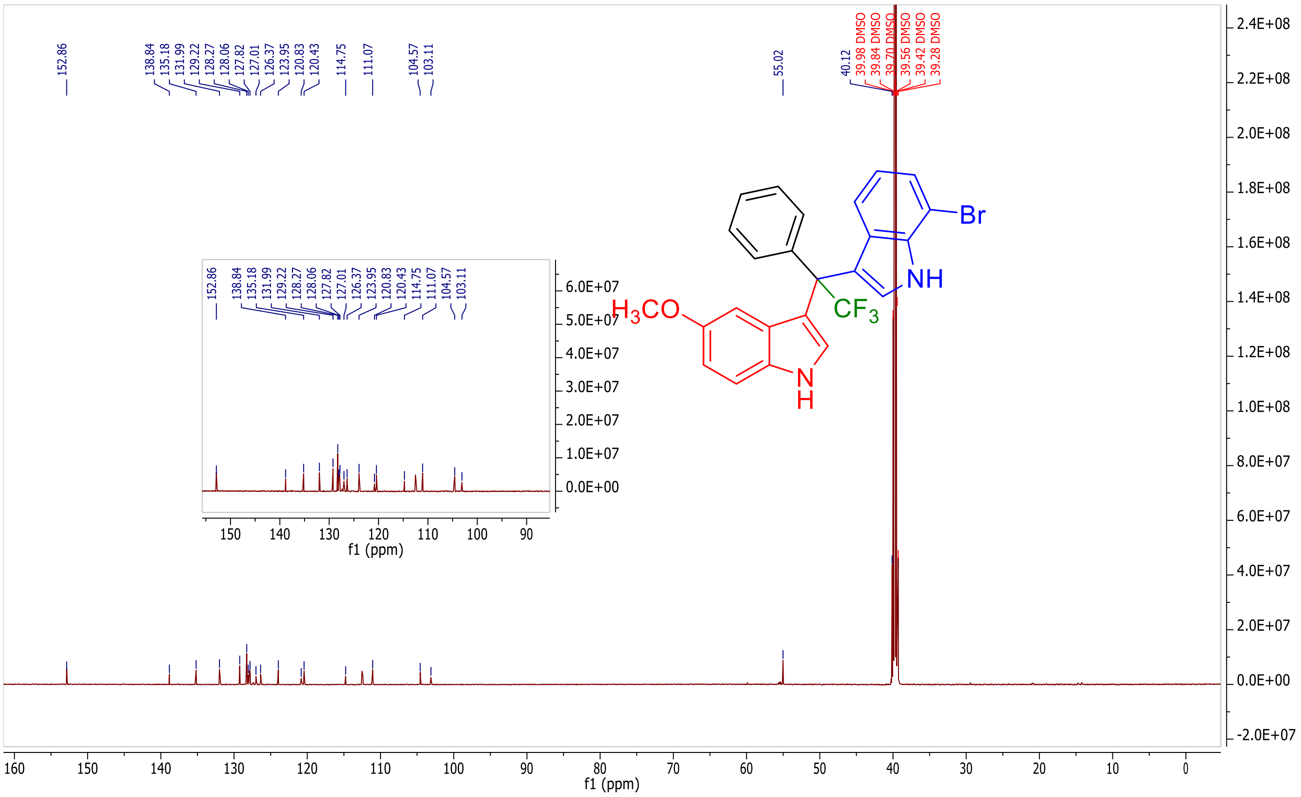

Figure S10. ^{1}H (600 MHz) and ^{13}C (151 MHz) Spectra of 7-bromo-3-(2,2,2-trifluoro-1-(5-methoxy-1H-indol-3-yl)-1-phenylethyl)-1H-indole (**3g**)

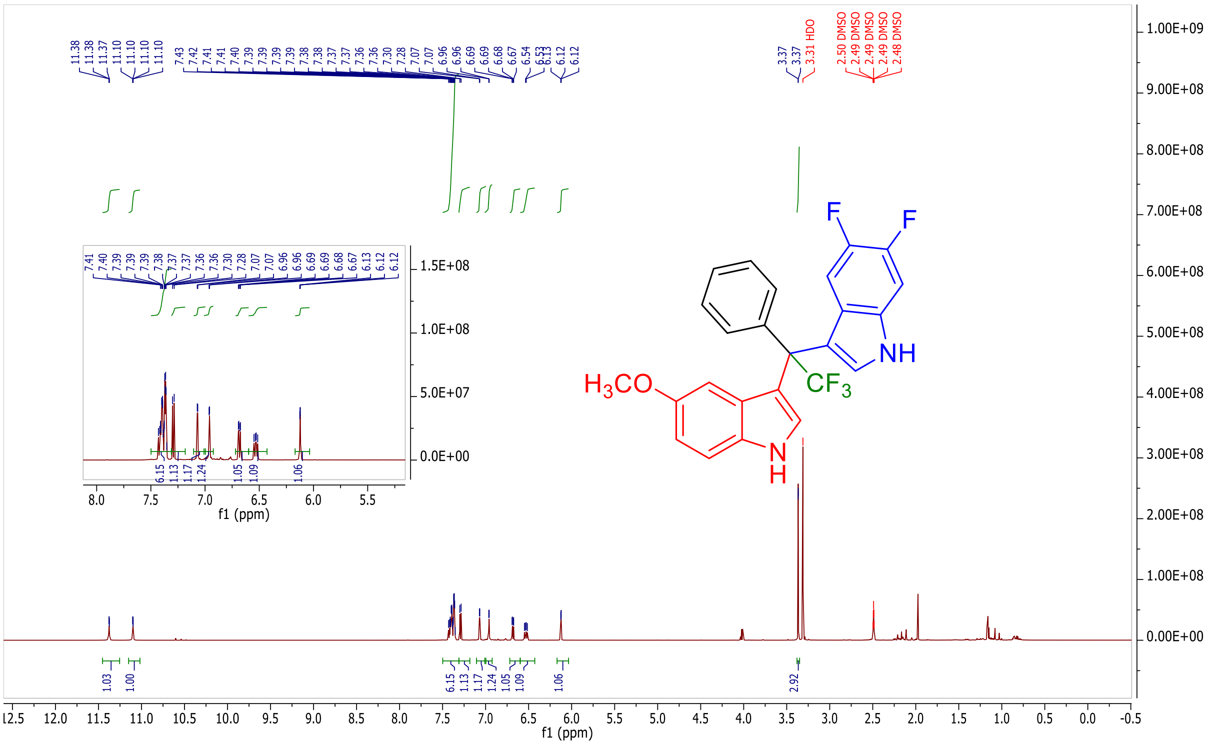

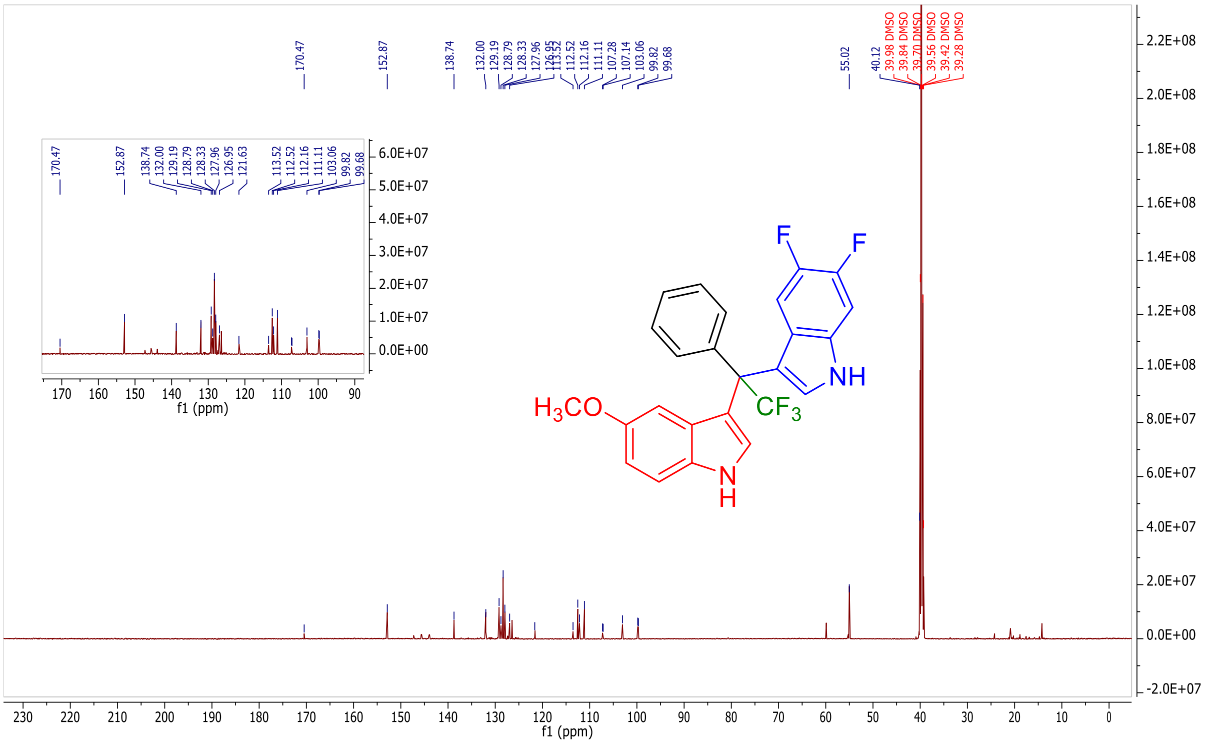

Figure S11. ^{1}H (600 MHz) and ^{13}C (151 MHz) Spectra of 5,6-difluoro-3-(2,2,2-trifluoro-1-(5-methoxy-1*H*-indol-3-yl)-1-phenylethyl)-1*H*-indole (**3h**)

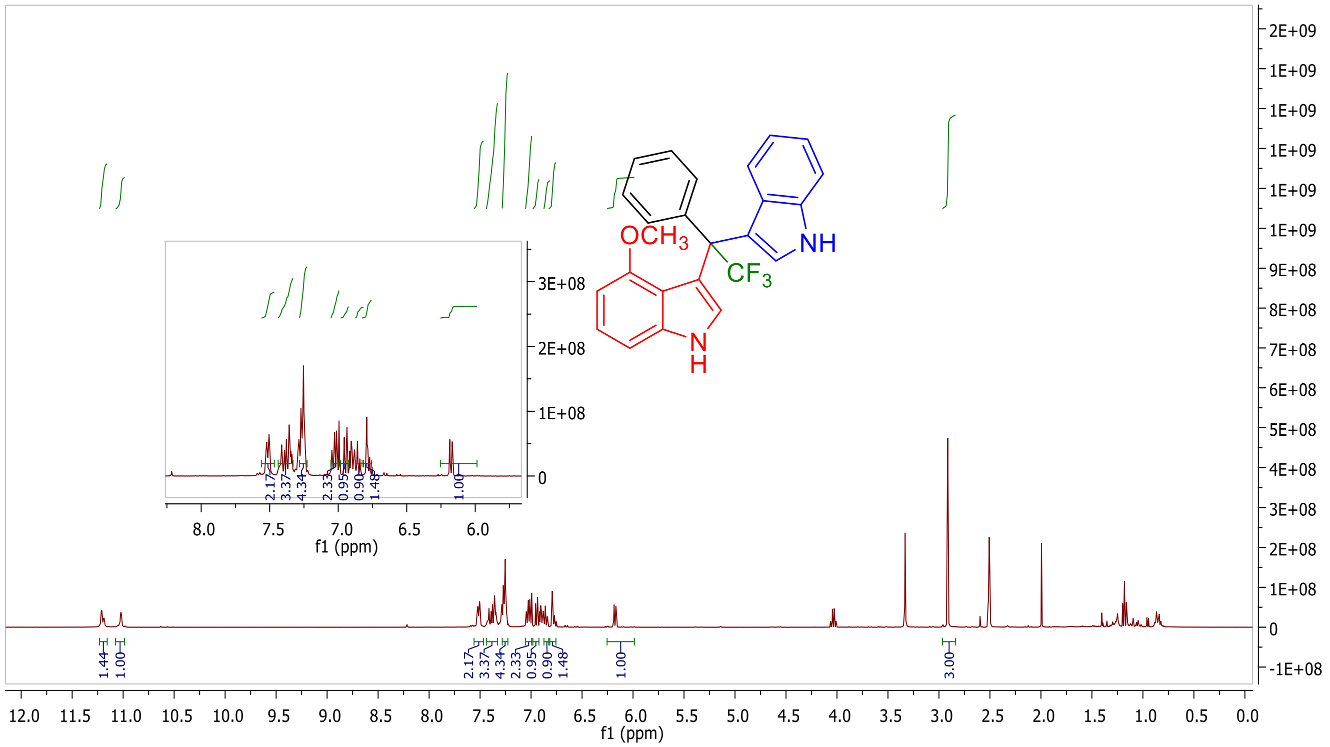

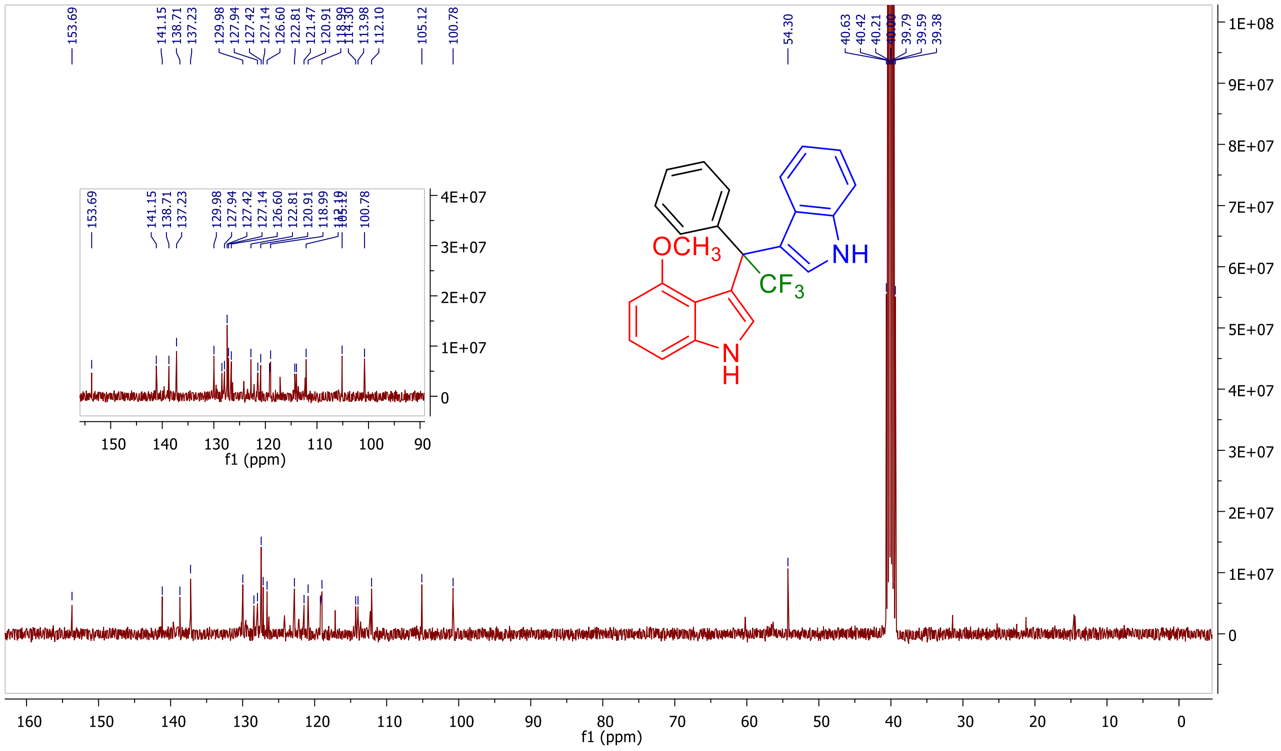

Figure S12. ^{1}H (600 MHz) and ^{13}C (151 MHz) Spectra of 4-methoxy-3-(2,2,2-trifluoro-1-(1*H*-indol-3-yl)-1-phenylethyl)-1*H*-indole (**3i**)

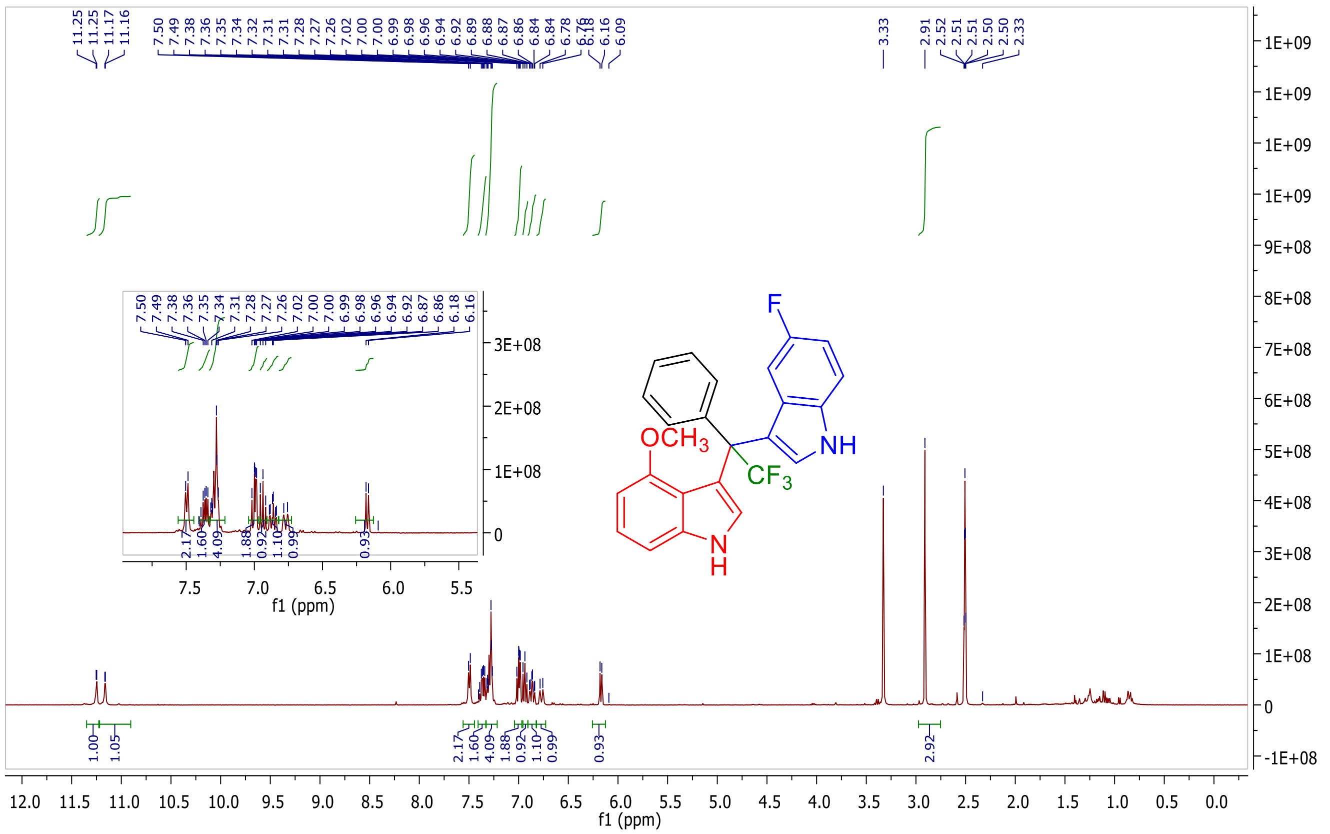

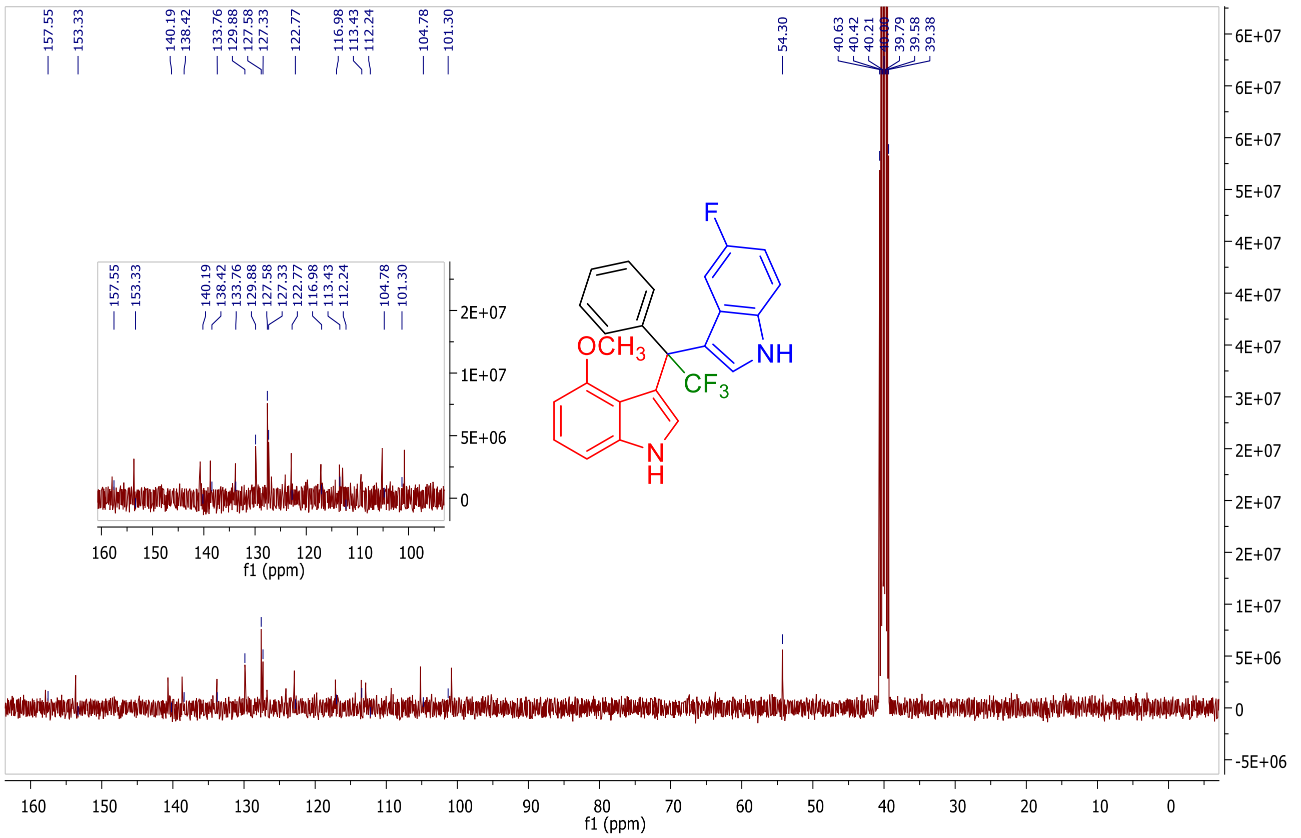

Figure S13. ^{1}H (600 MHz) and ^{13}C (151 MHz) Spectra of 5-fluoro-3-(2,2,2-trifluoro-1-(4-methoxy-1*H*-indol-3-yl)-1-phenylethyl)-1*H*-indole (**3j**)

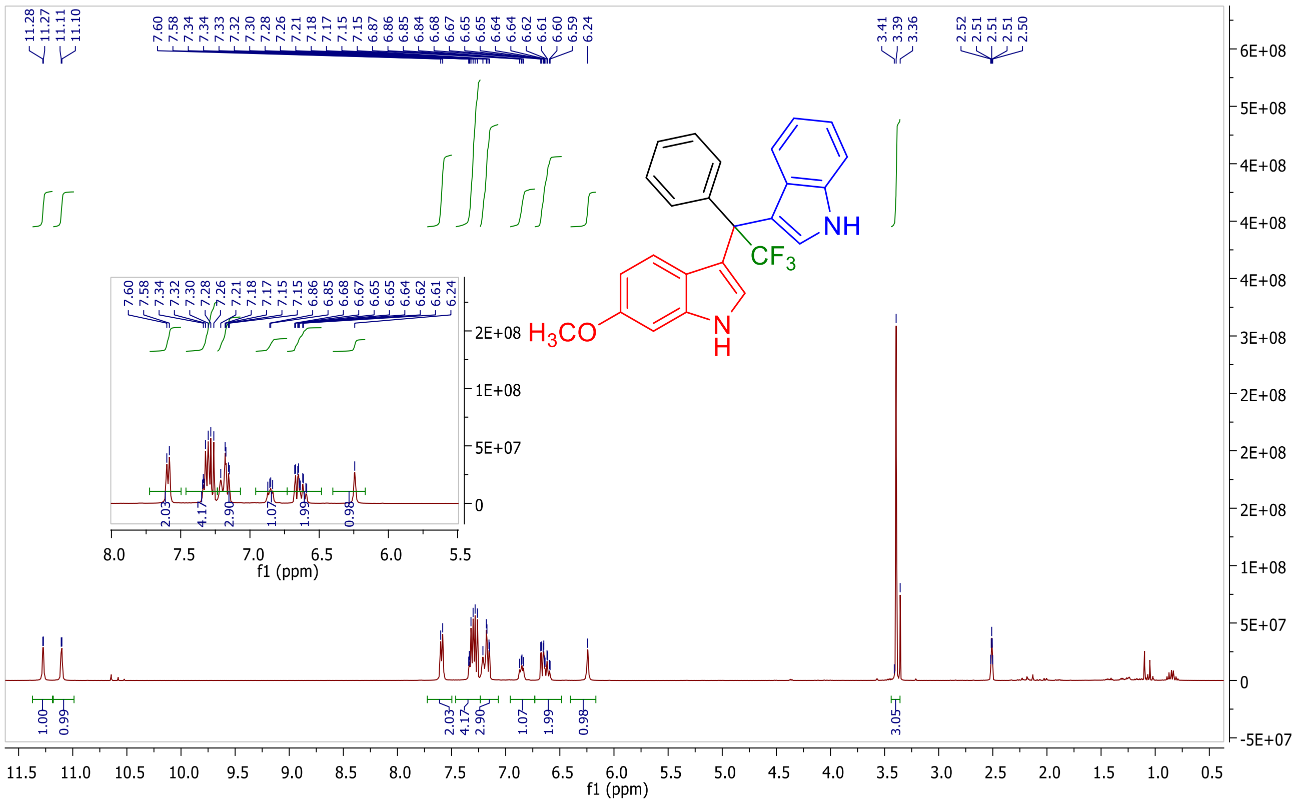

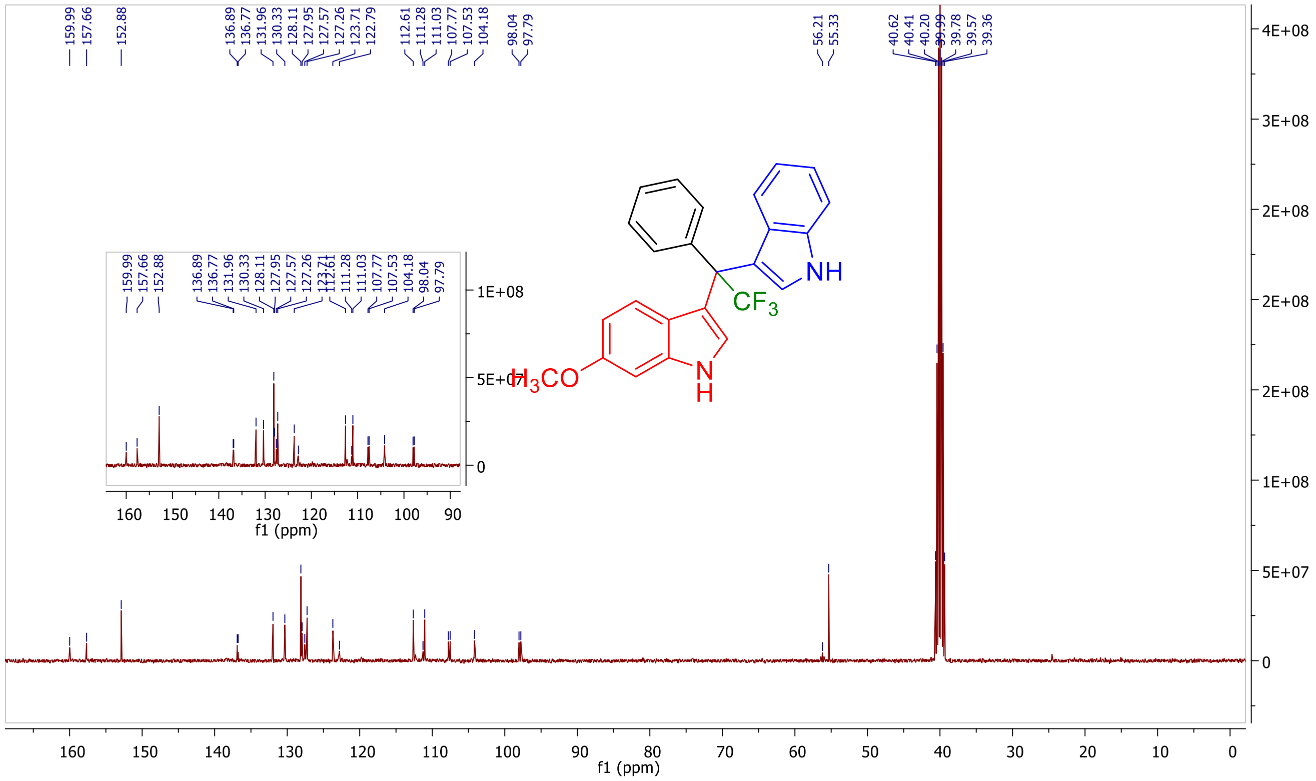

Figure S14. ^{1}H (600 MHz) and ^{13}C (151 MHz) Spectra of 6-methoxy-3-(2,2,2-trifluoro-1-(1*H*-indol-3-yl)-1-phenylethyl)-1*H*-indole (**3k**)

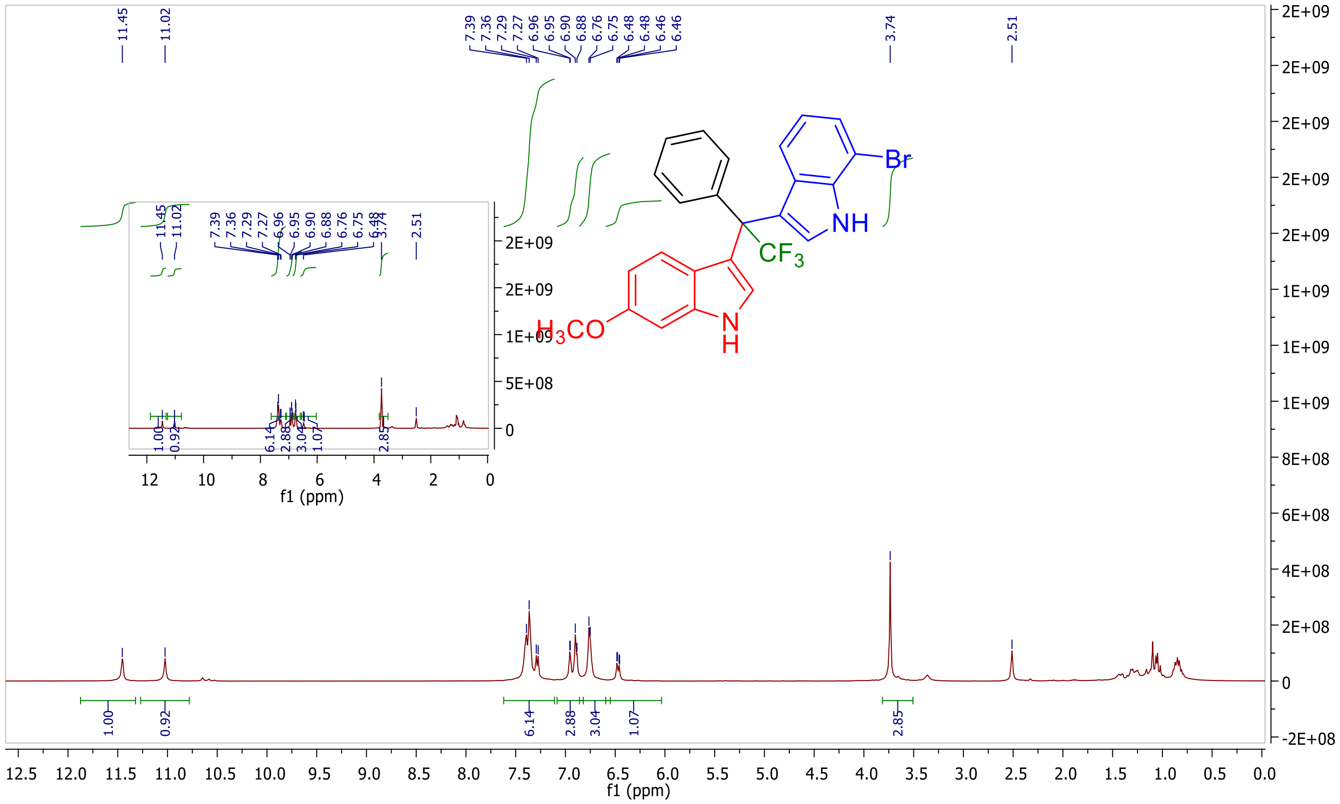

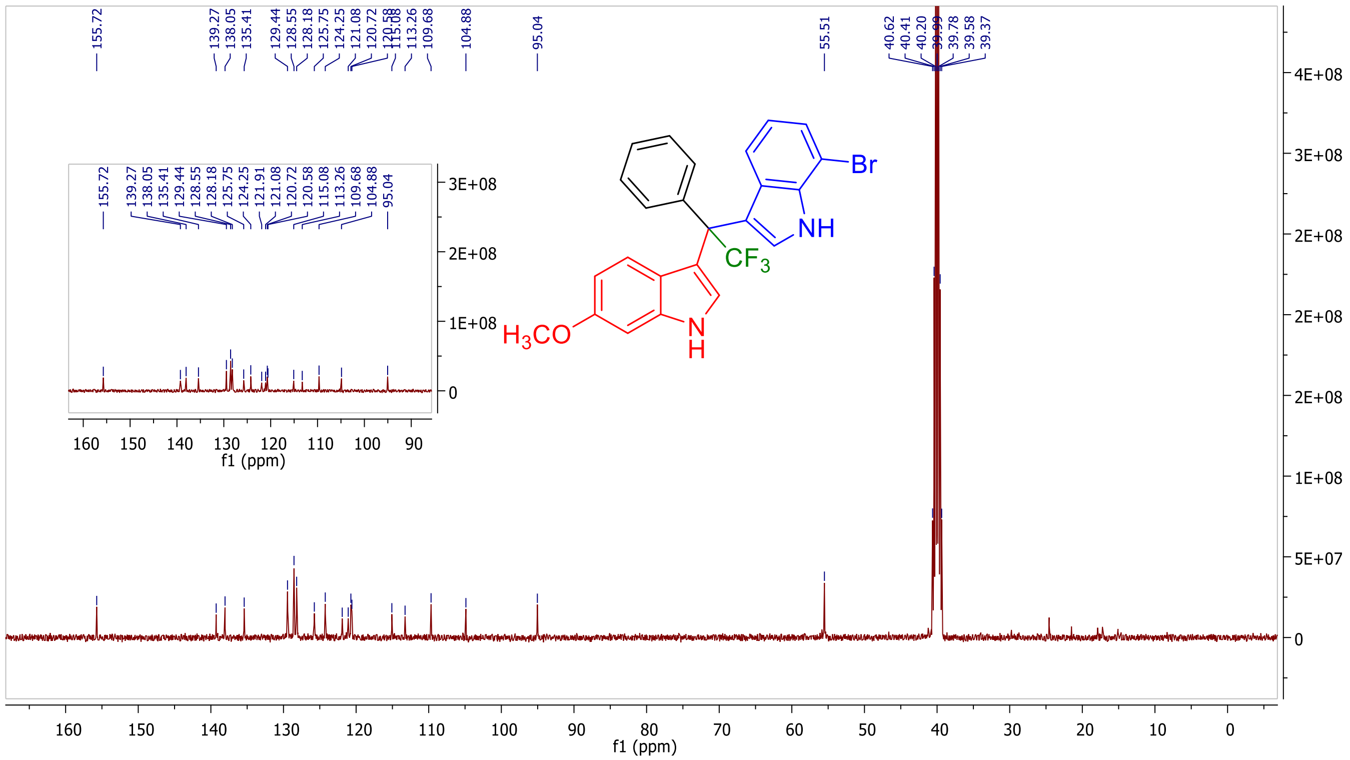

Figure S15. ^{1}H (600 MHz) and ^{13}C (151 MHz) Spectra of 7-bromo-3-(2,2,2-trifluoro-1-(6-methoxy-1H-indol-3-yl)-1-phenylethyl)-1H-indole (**3l**)

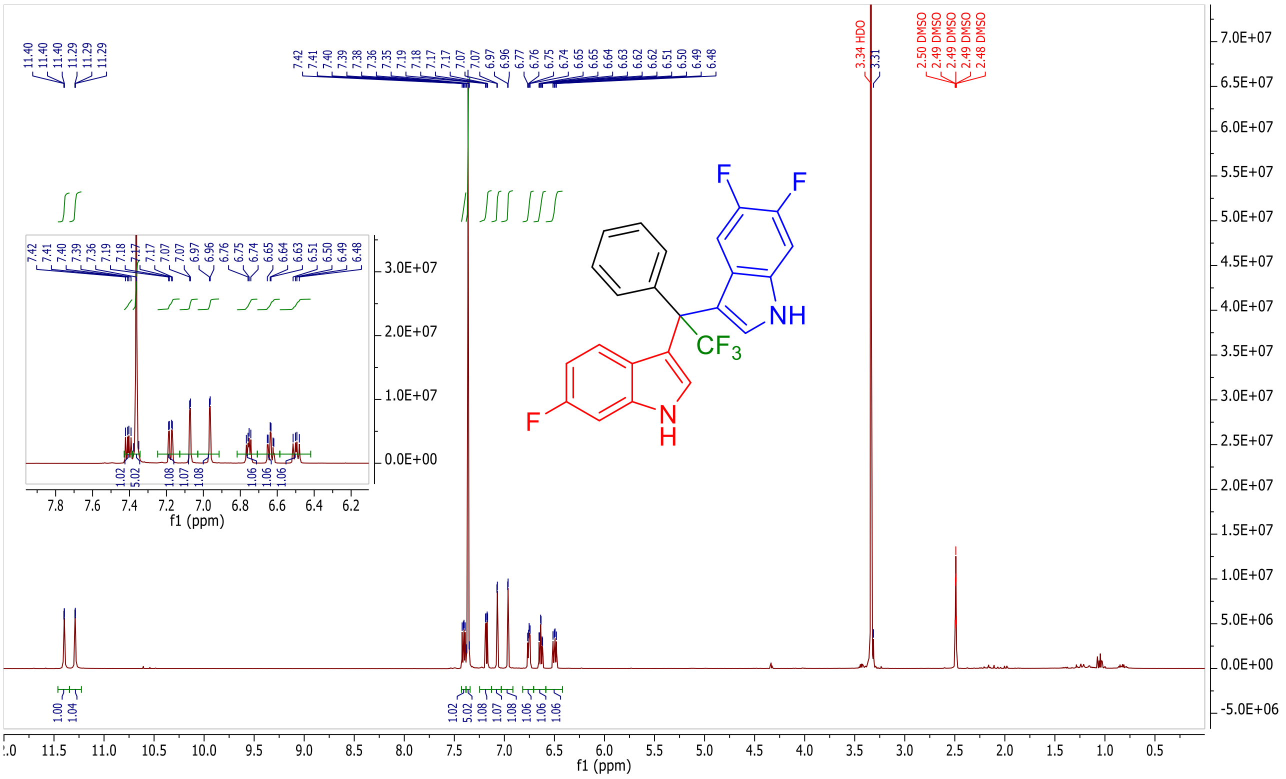

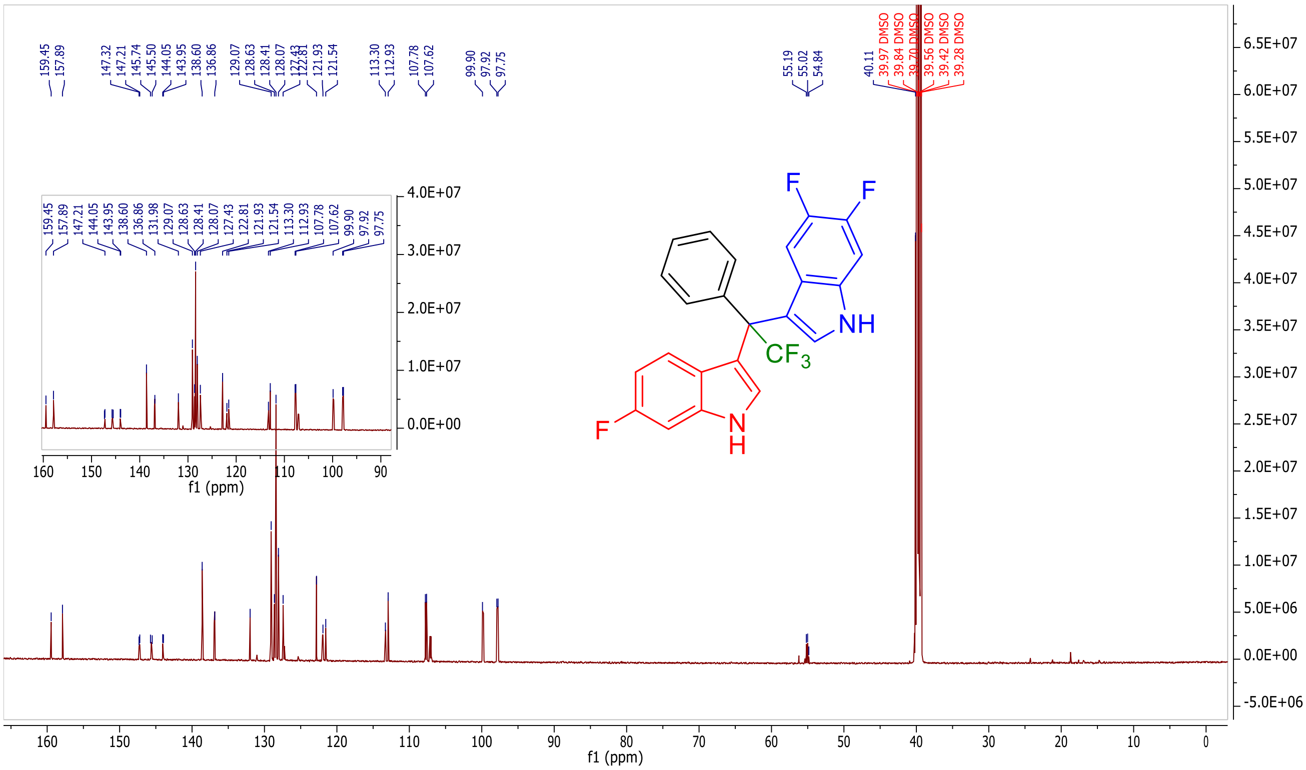

Figure S16. ^{1}H (600 MHz) and ^{13}C (151 MHz) Spectra of 5,6-difluoro-3-(2,2,2-trifluoro-1-(6-fluoro-1*H*-indol-3-yl)-1-phenylethyl)-1*H*-indole (**3m**)

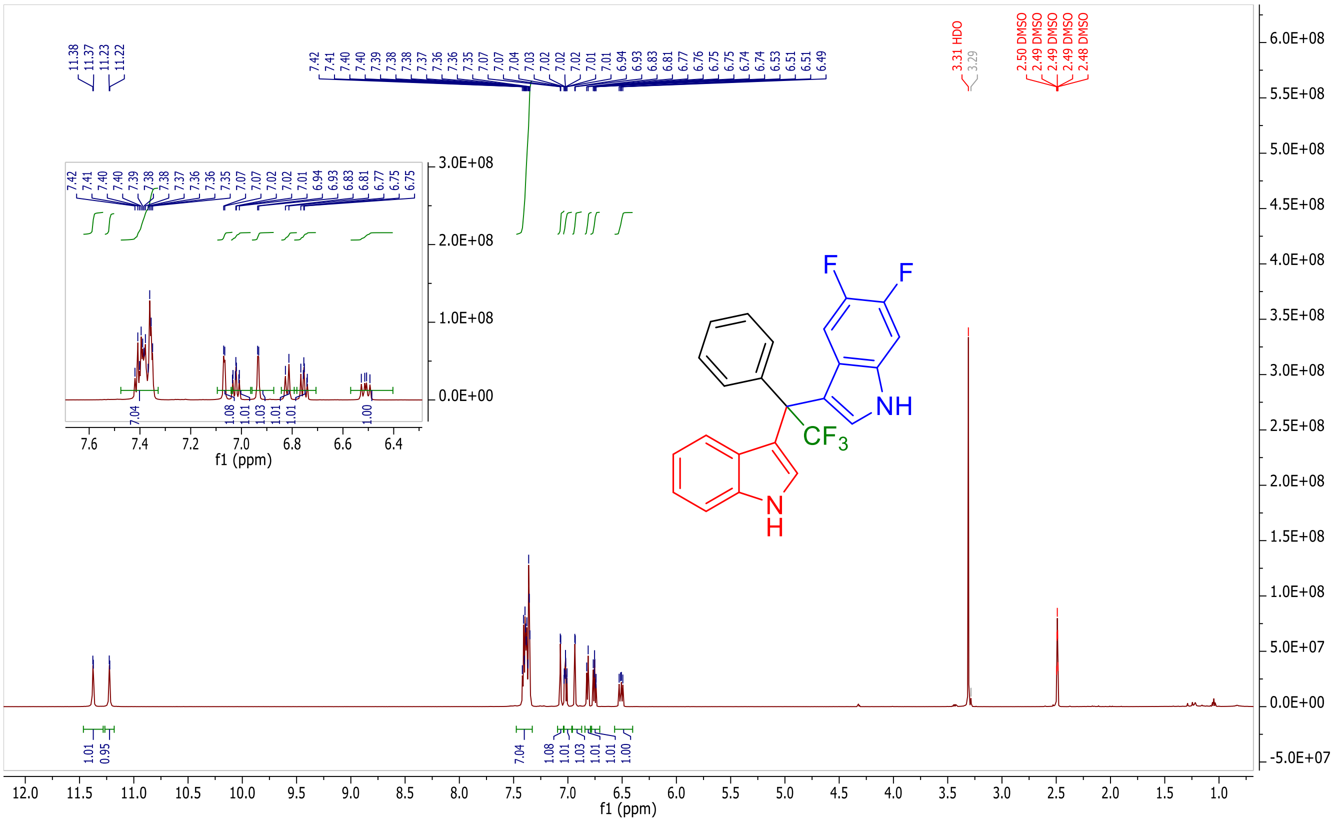

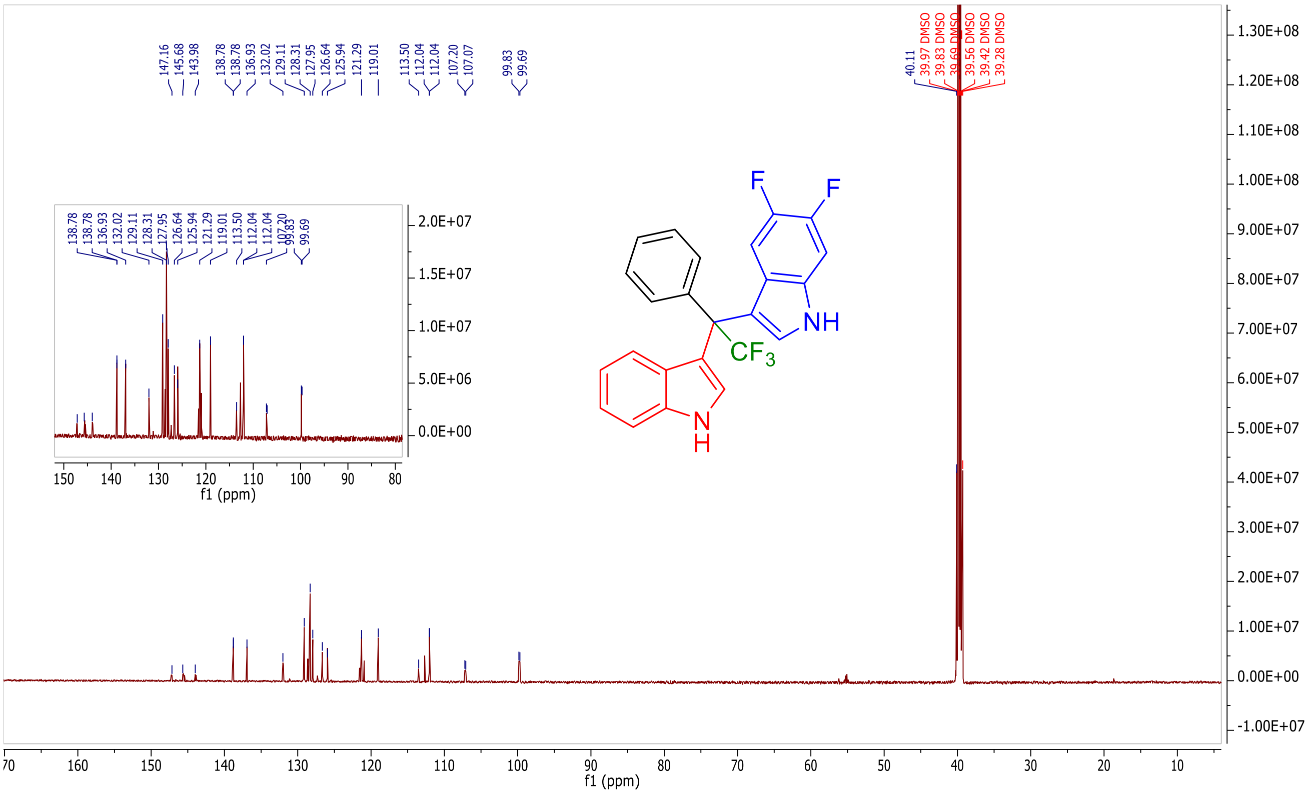

Figure S17. ^{1}H (600 MHz) and ^{13}C (151 MHz) Spectra of 5,6-difluoro-3-(2,2,2-trifluoro-1-(1*H*-indol-3-yl)-1-phenylethyl)-1H-indole (**3n**)

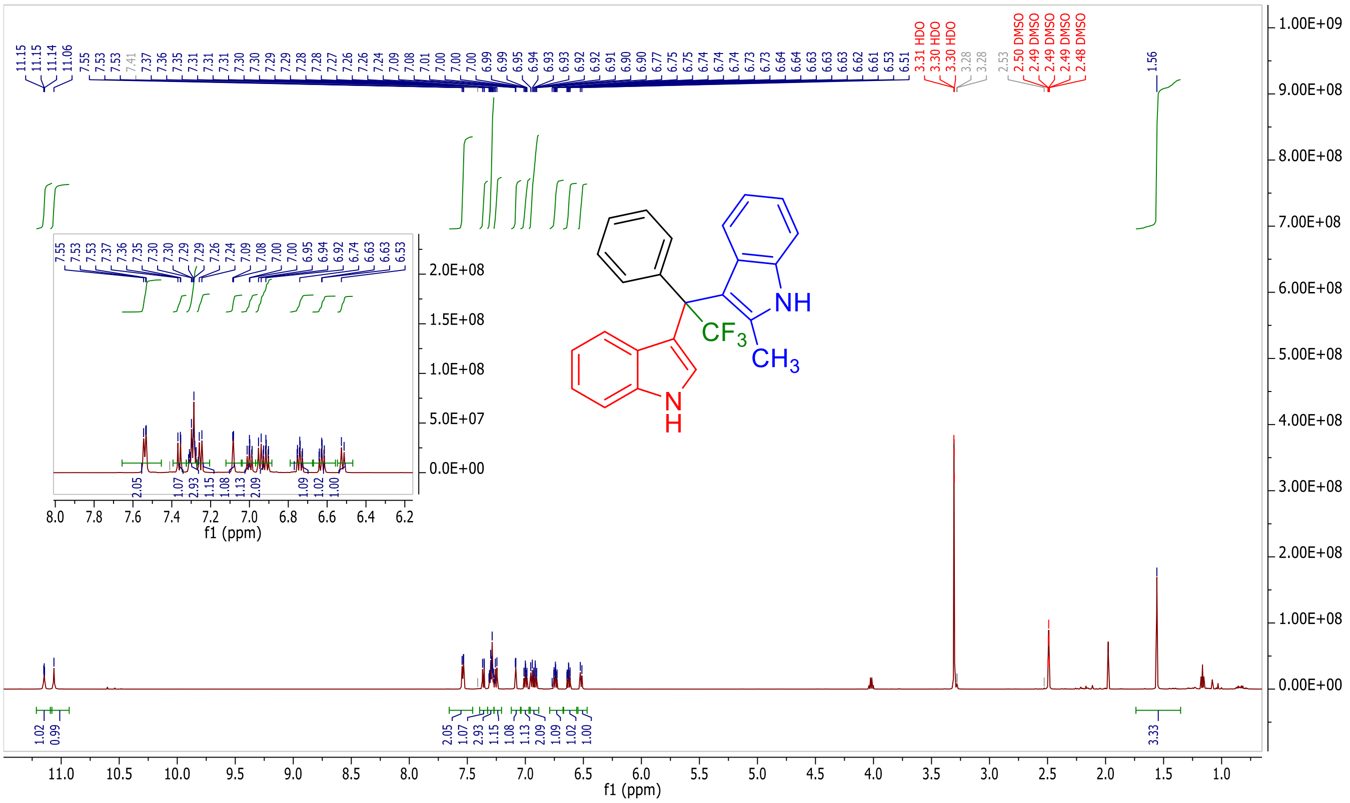

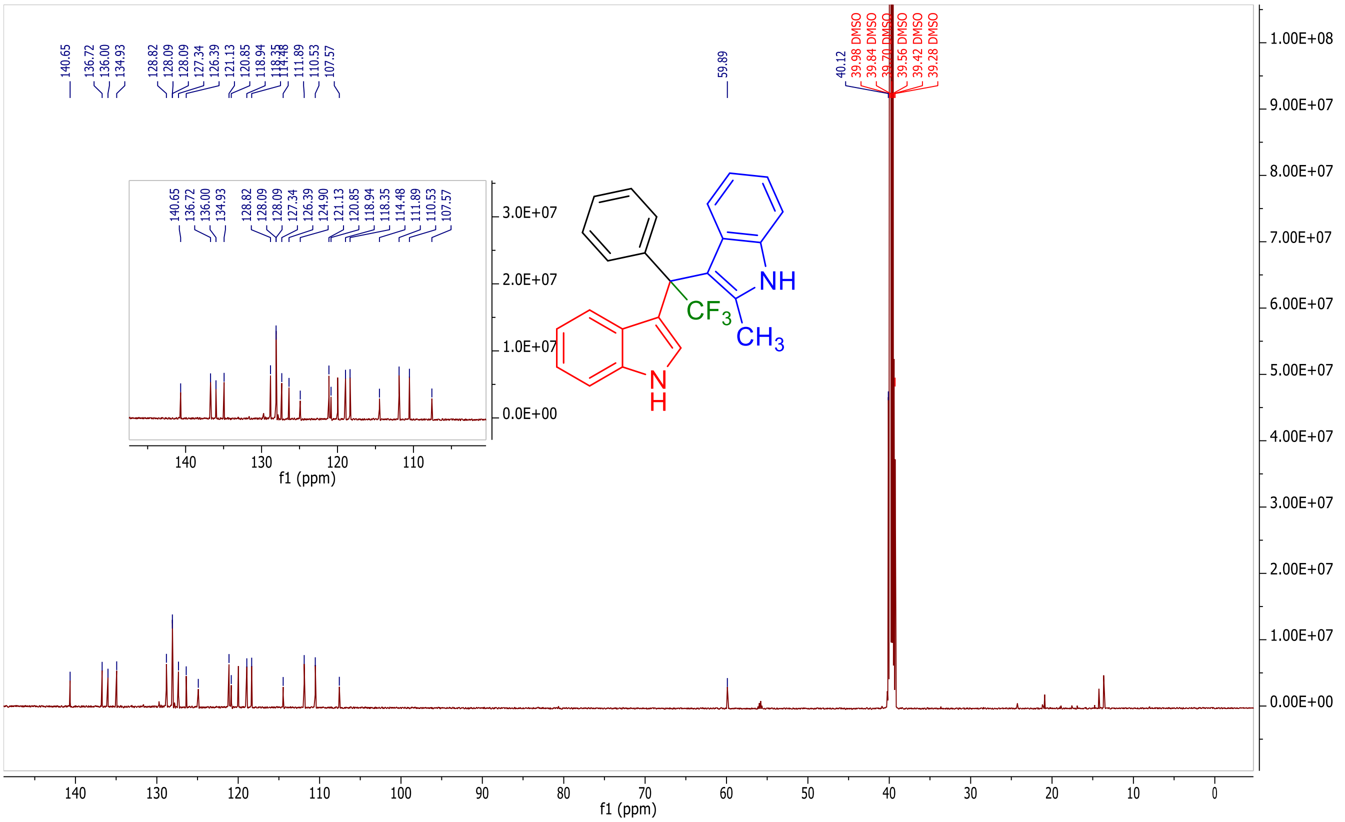

Figure S18. ^{1}H (600 MHz) and ^{13}C (151 MHz) Spectra of 2-methyl-3-(2,2,2-trifluoro-1-(*1H*-indol-3-yl)-1-phenylethyl)-1*H*-indole (**3o**)

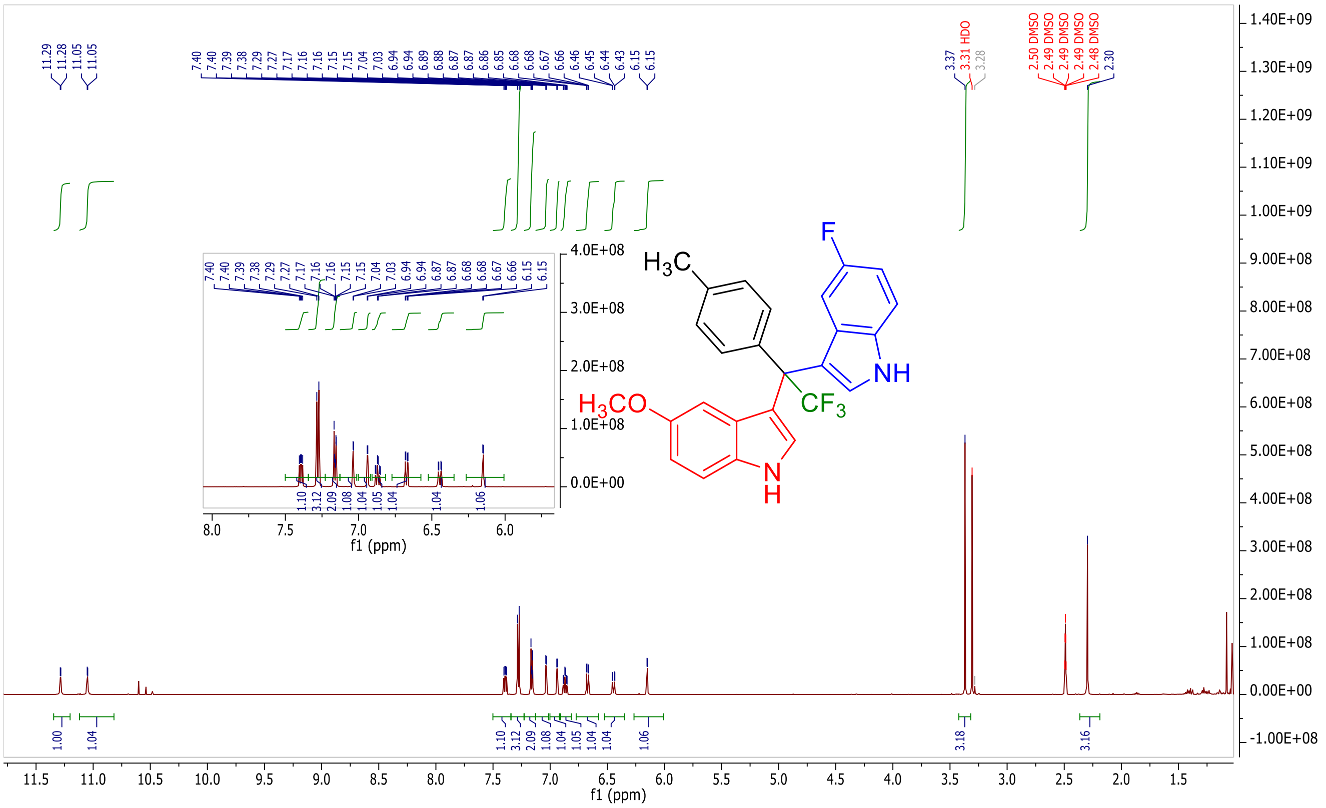

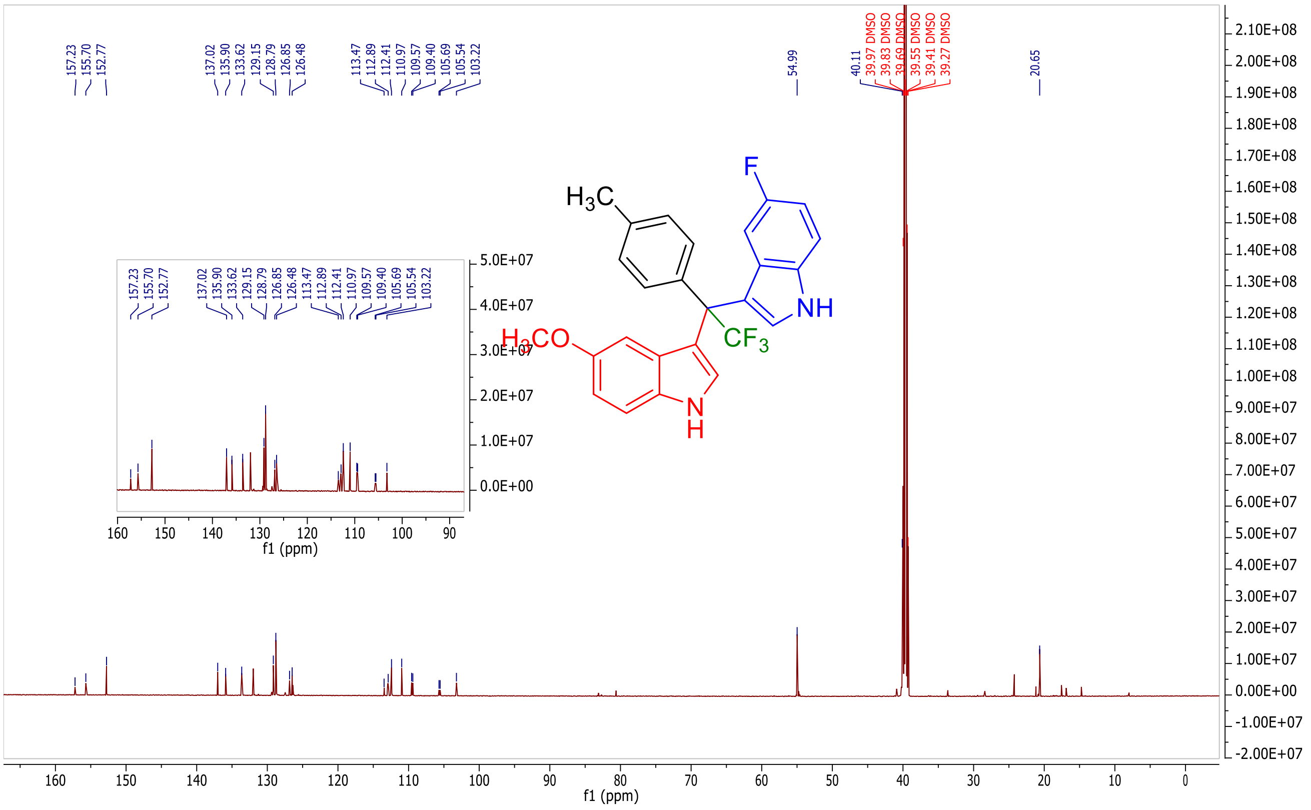

Figure S19. ^{1}H (600 MHz) and ^{13}C (151 MHz) Spectra of 5-fluoro-3-(2,2,2-trifluoro-1-(5-methoxy-1*H*-indol-3-yl)-1-(*p*-tolyl)ethyl)-1*H*-indole (**3p**)

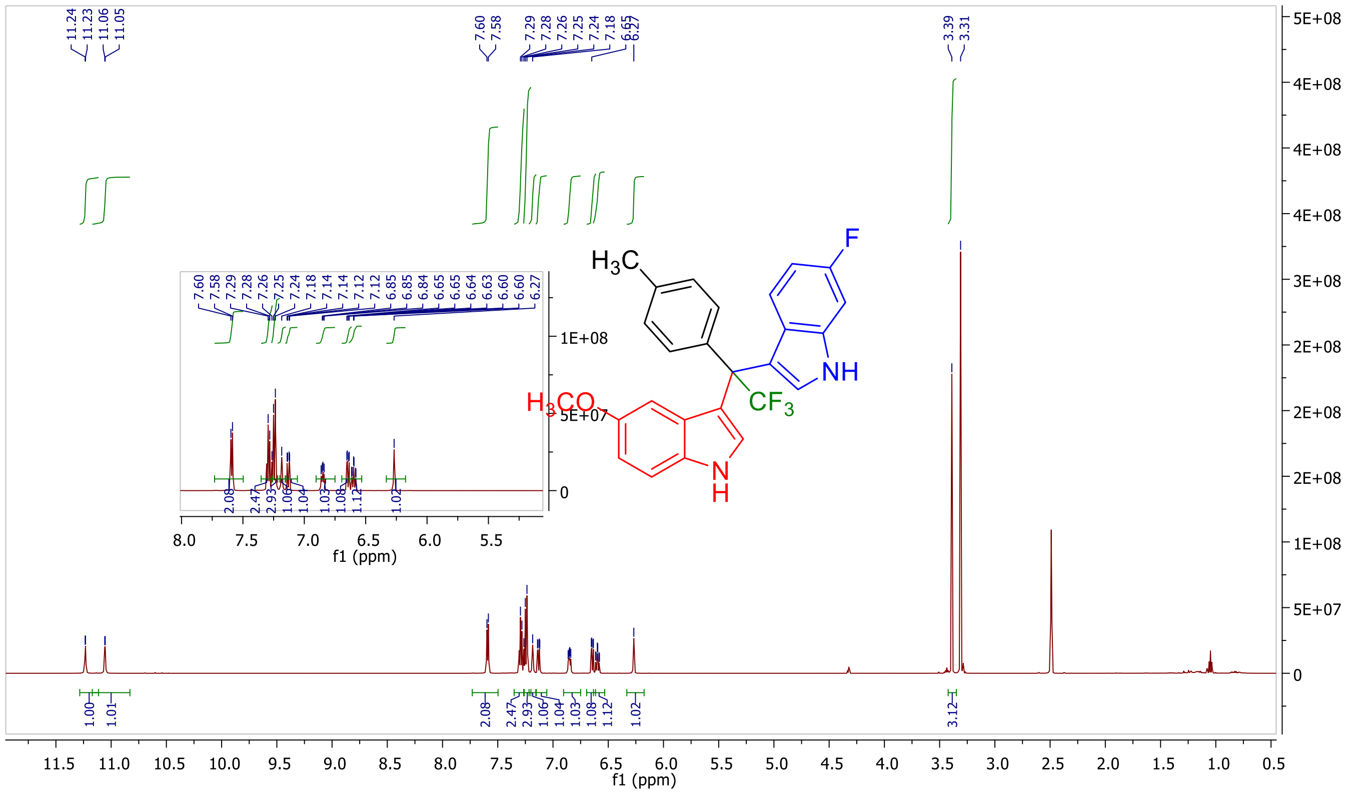

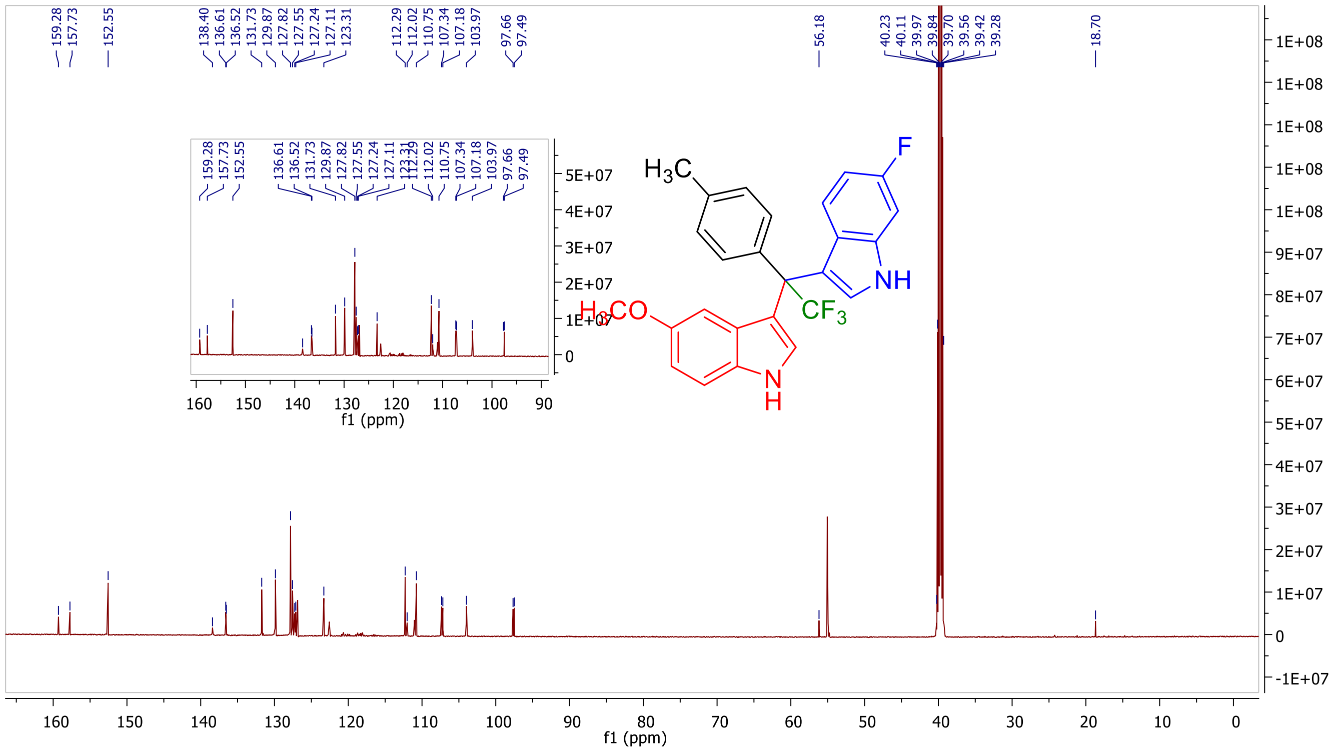

Figure S20. ^{1}H (600 MHz) and ^{13}C (151 MHz) Spectra of 6-fluoro-3-(2,2,2-trifluoro-1-(5-methoxy-1*H*-indol-3-yl)-1-(*p*-tolyl)ethyl)-1*H*-indole (**3q**)

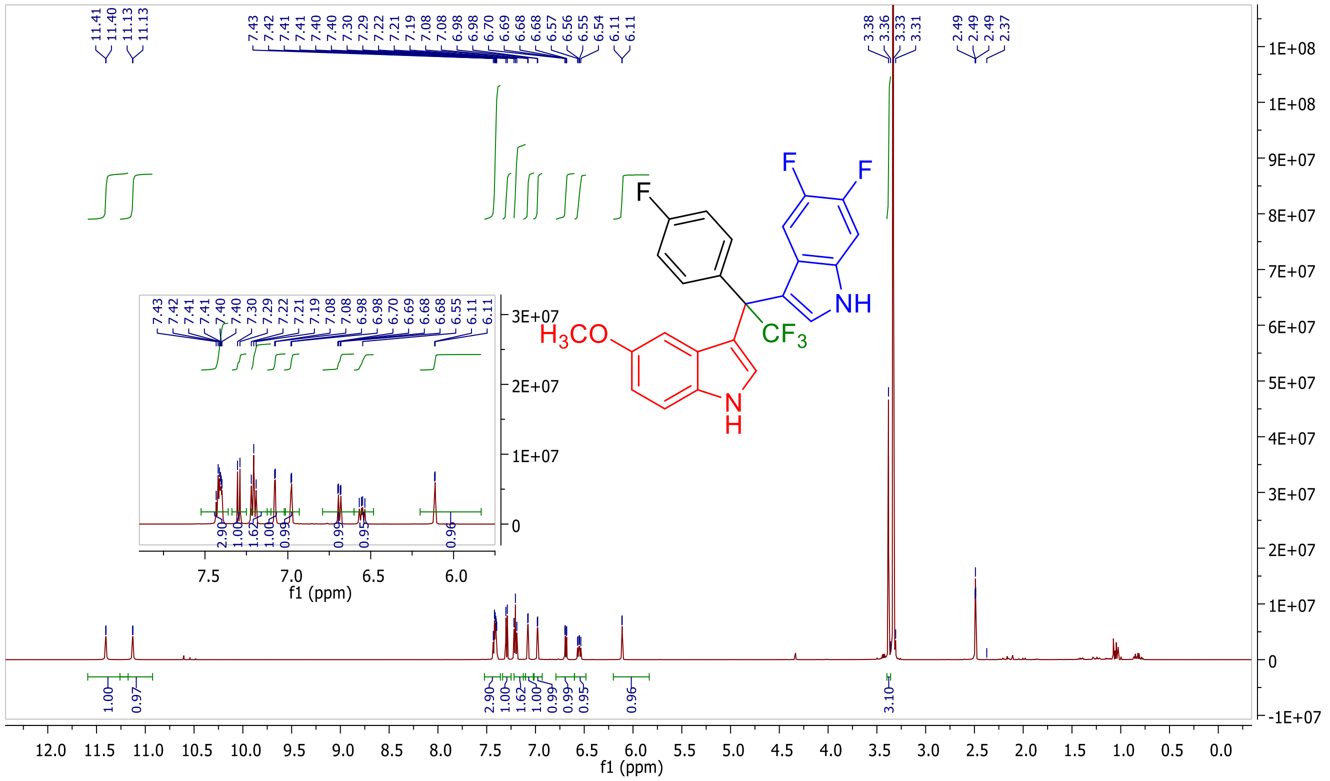

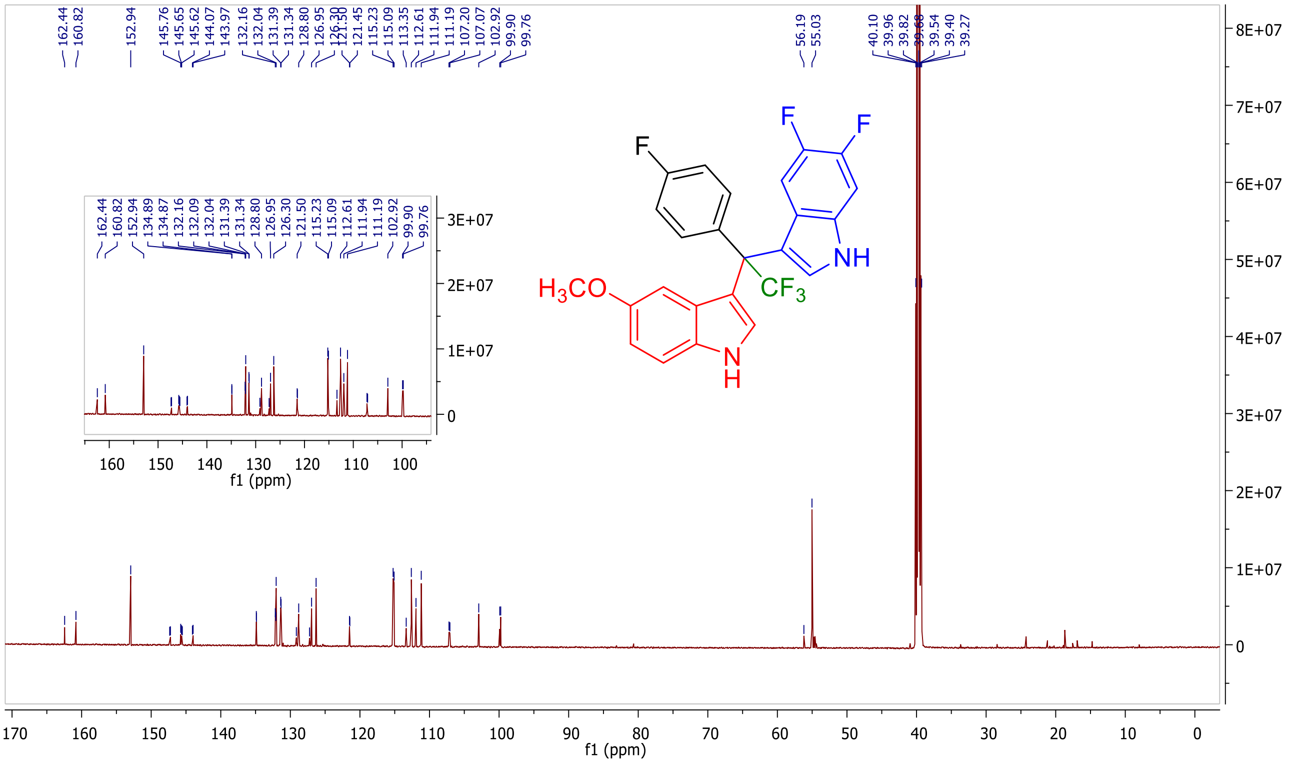

Figure S21. ^{1}H (600 MHz) and ^{13}C (151 MHz) Spectra of 5,6-difluoro-3-(2,2,2-trifluoro-1-(4-fluorophenyl)-1-(5-methoxy-1*H*-indol-3-yl)ethyl)-1*H*-indole (**3r**)

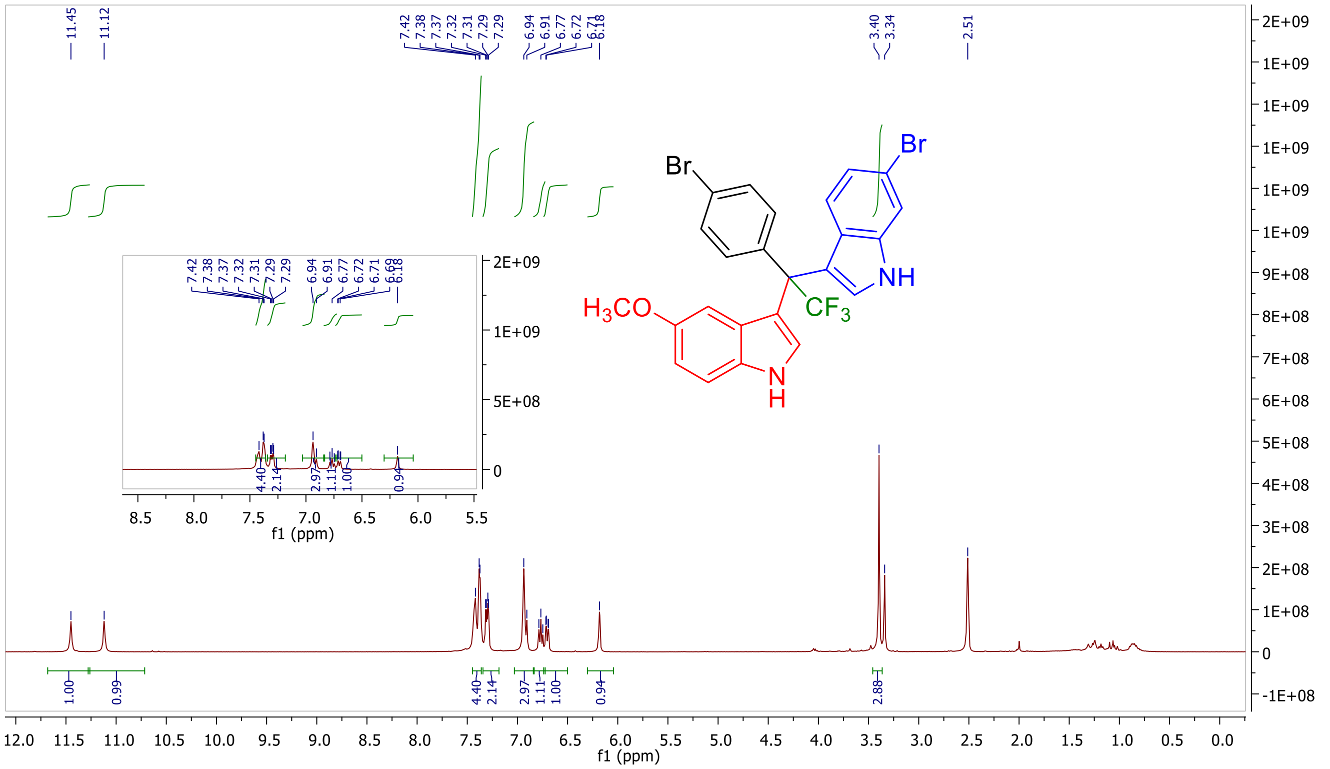

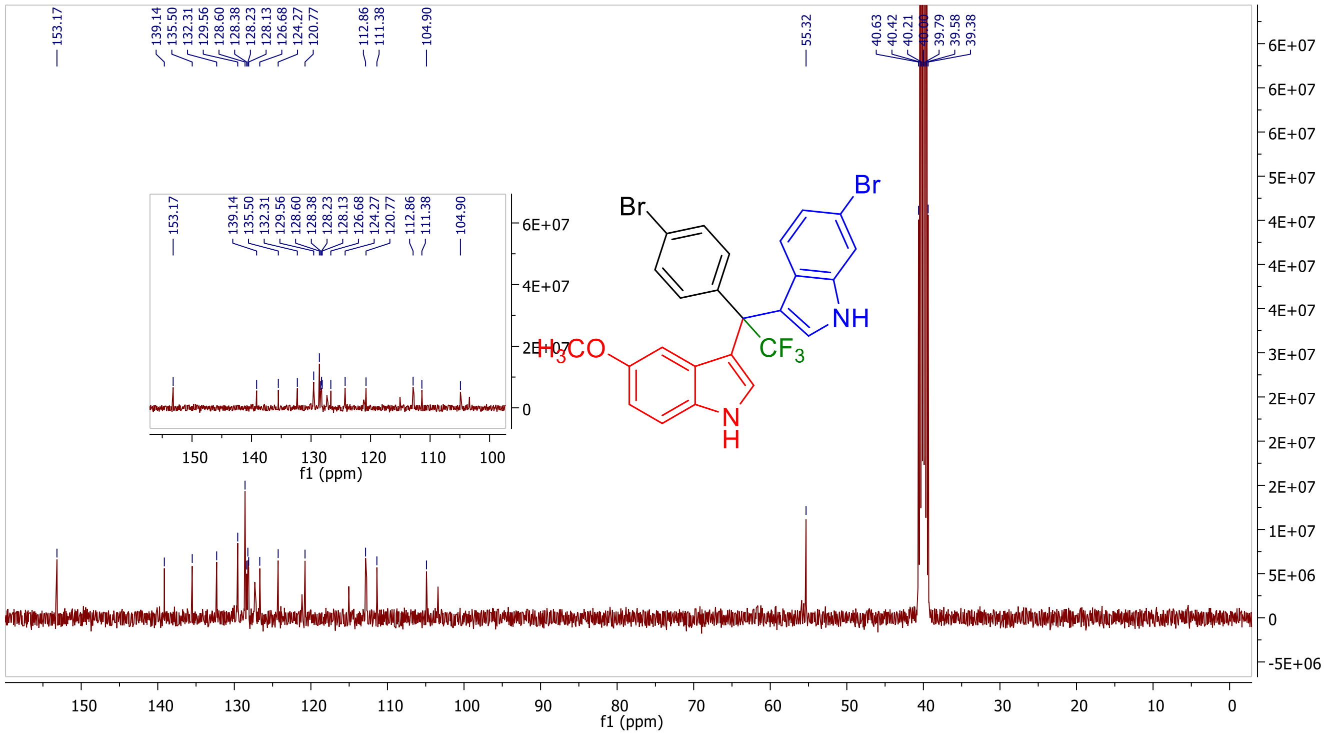

Figure S22. ^{1}H (600 MHz) and ^{13}C (151 MHz) Spectra of 6-bromo-3-(1-(4-bromophenyl)-2,2,2-trifluoro-1-(5-methoxy-1*H*-indol-3-yl)ethyl)-1*H*-indole (**3s**)

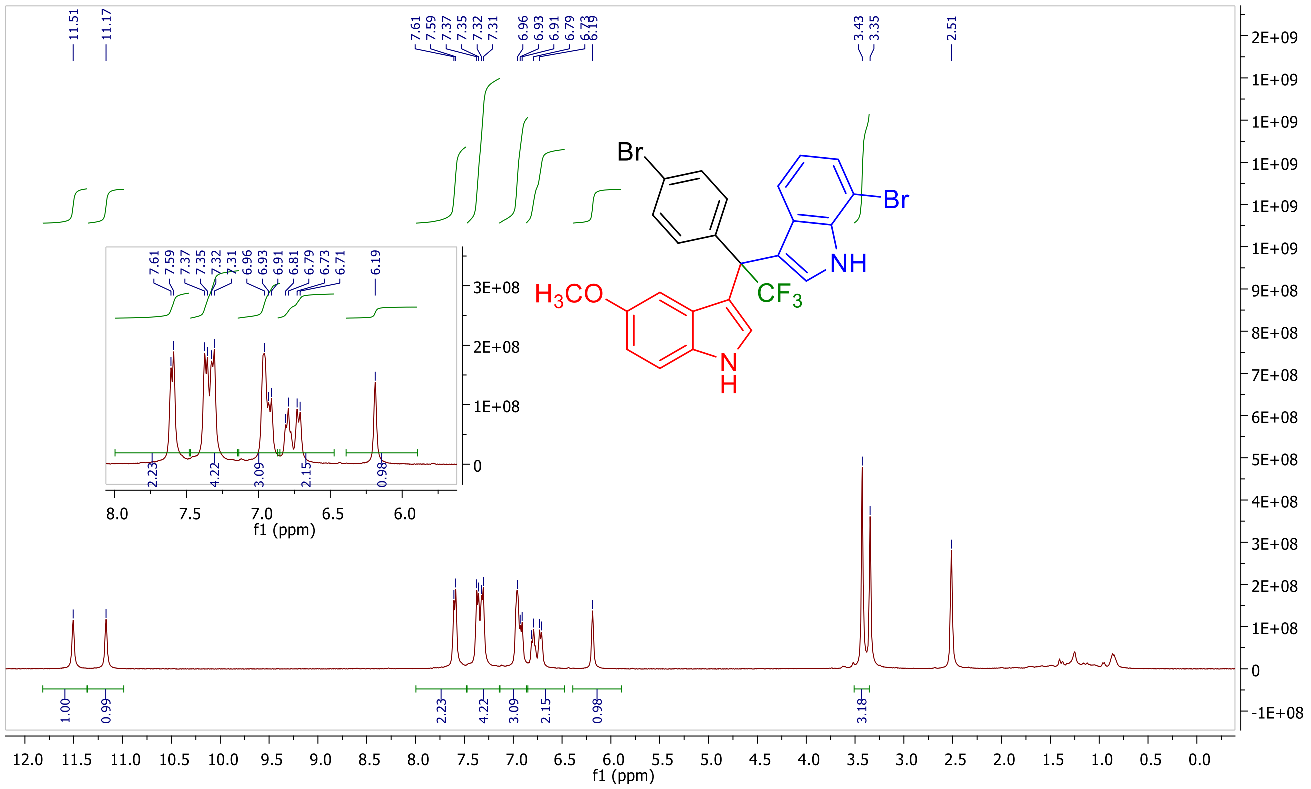

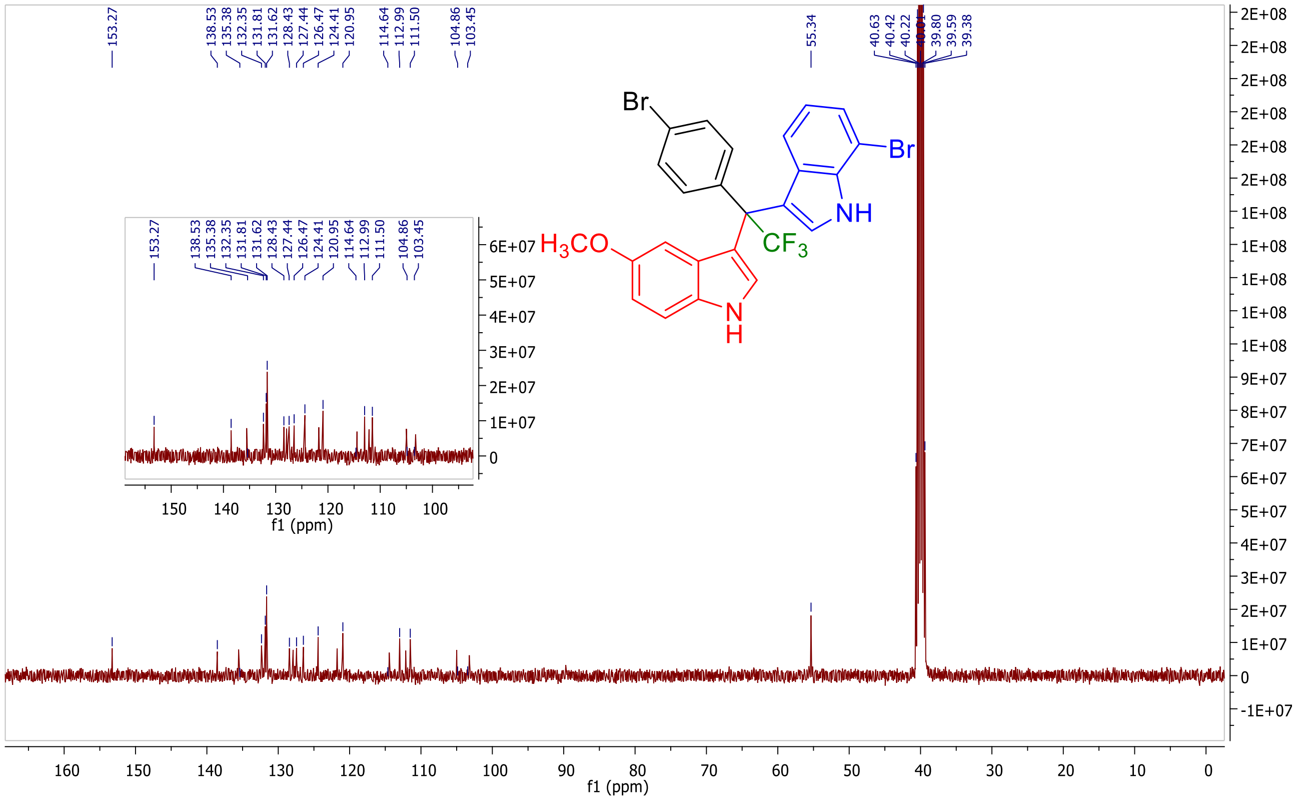

Figure S23. ^{1}H (600 MHz) and ^{13}C (151 MHz) Spectra of 7-bromo-3-(1-(4-bromophenyl)-2,2,2-trifluoro-1-(5-methoxy-1*H*-indol-3-yl)ethyl)-1*H*-indole (**3t**)

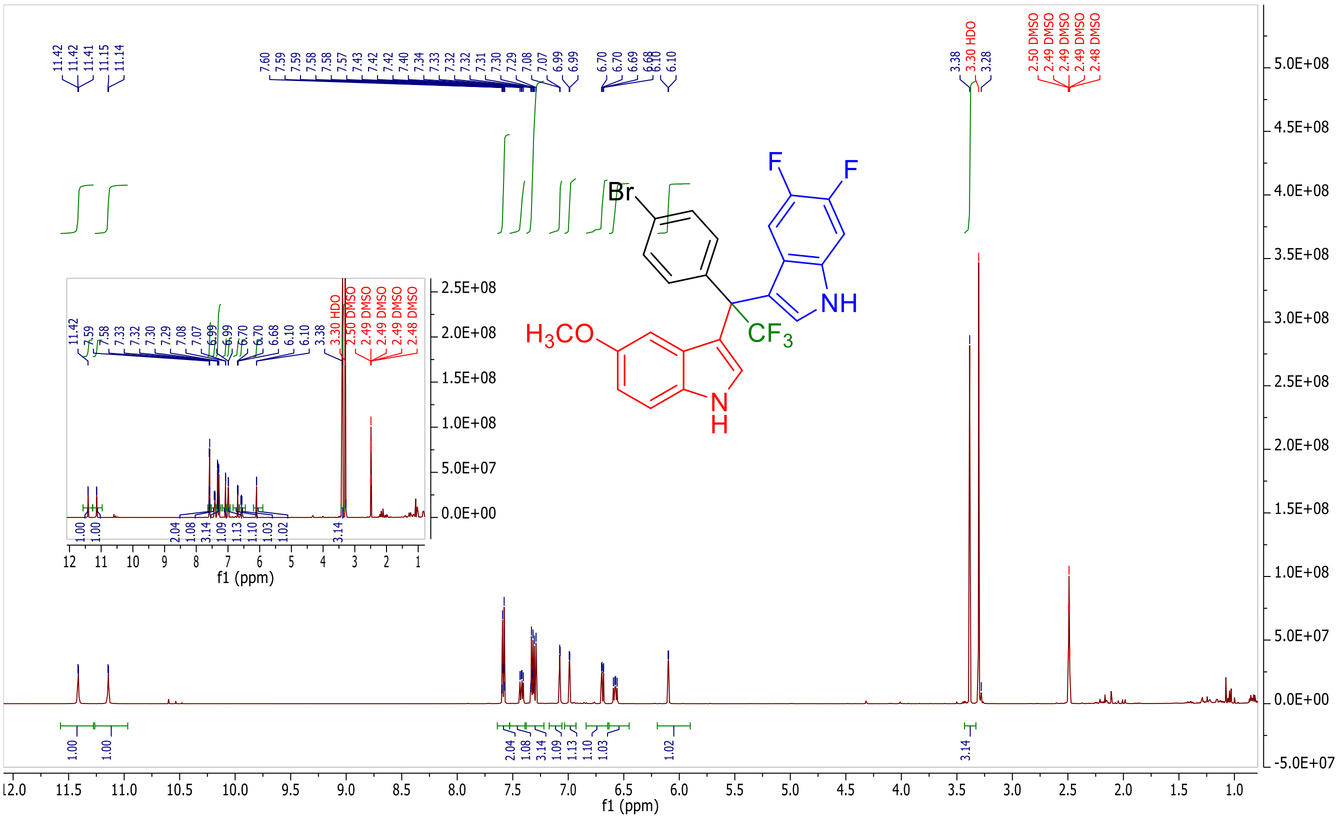

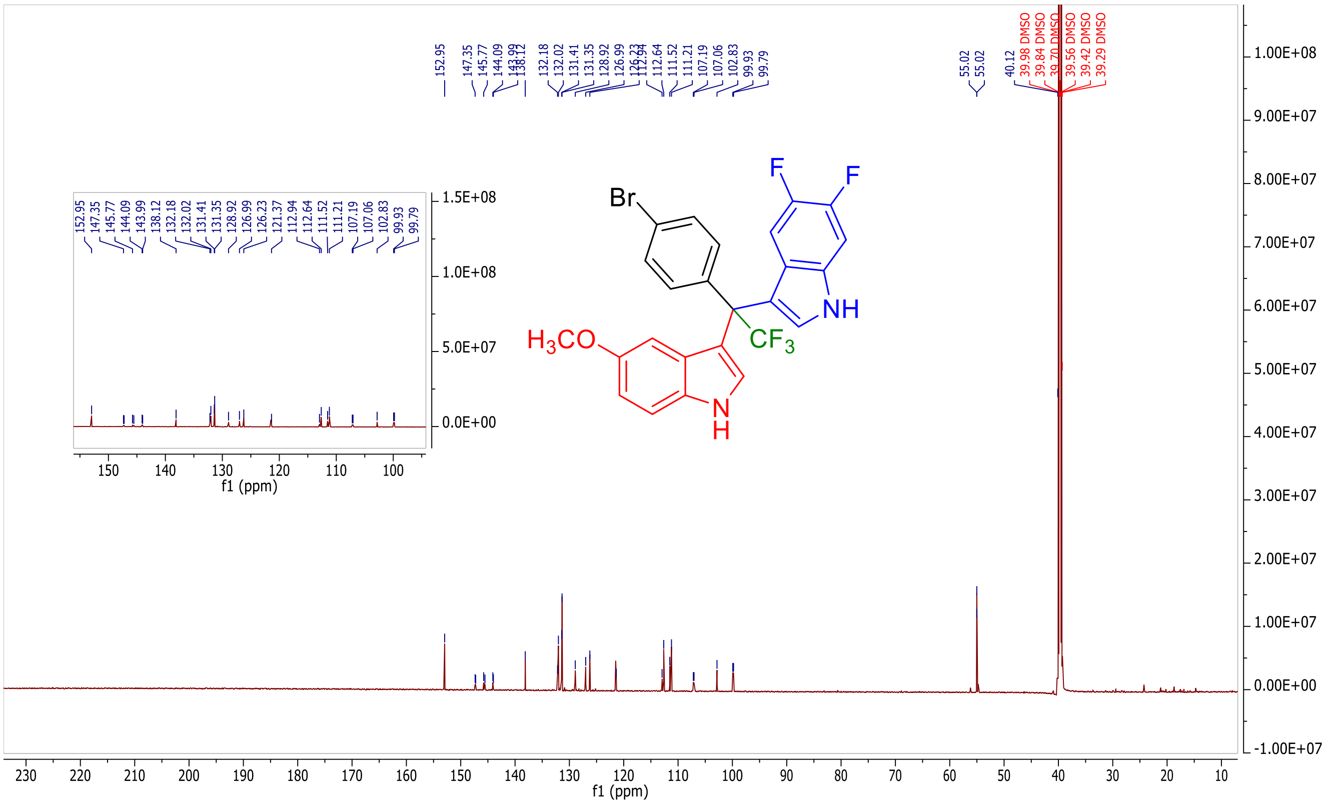

Figure S24. ^{1}H (600 MHz) and ^{13}C (151 MHz) Spectra of 3-(1-(4-bromophenyl)-2,2,2-trifluoro-1-(5-methoxy-1*H*-indol-3-yl)ethyl)-5,6-difluoro-1*H*-indole (**3u**)

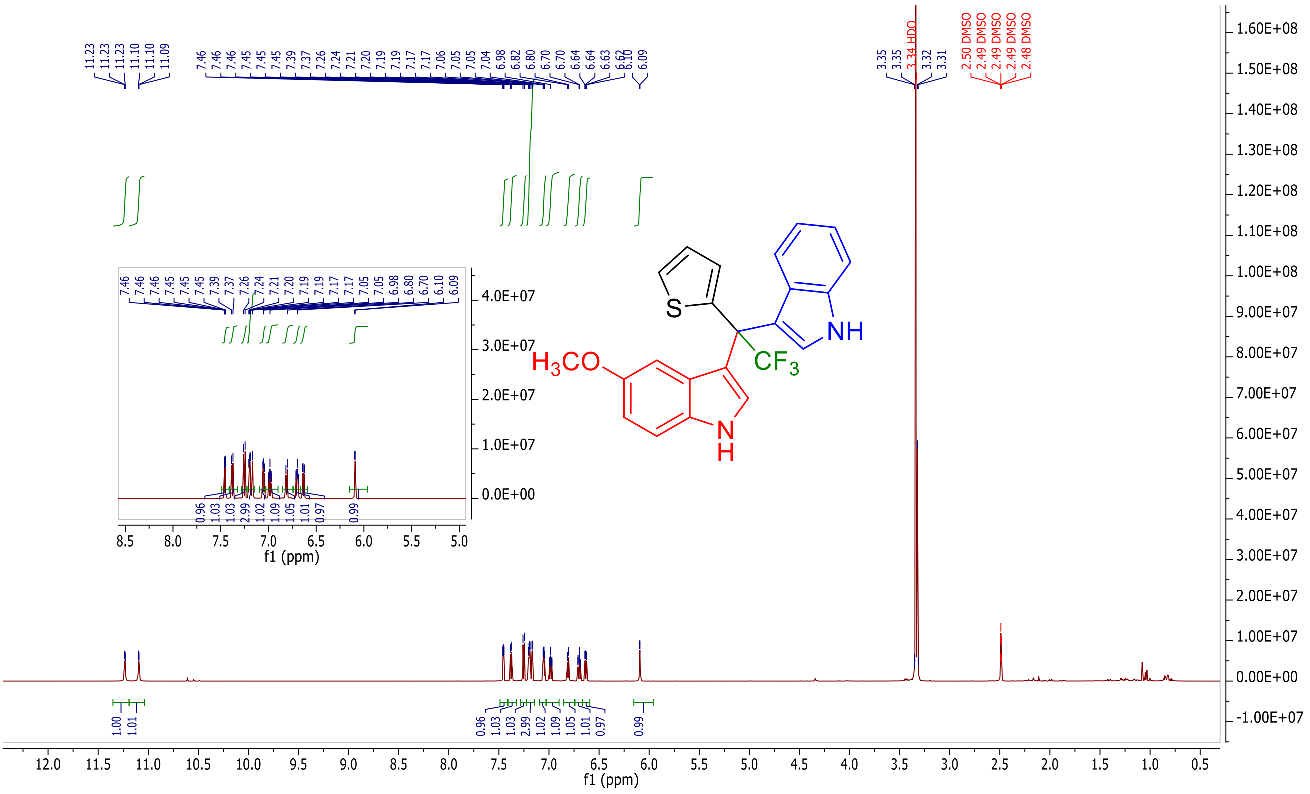

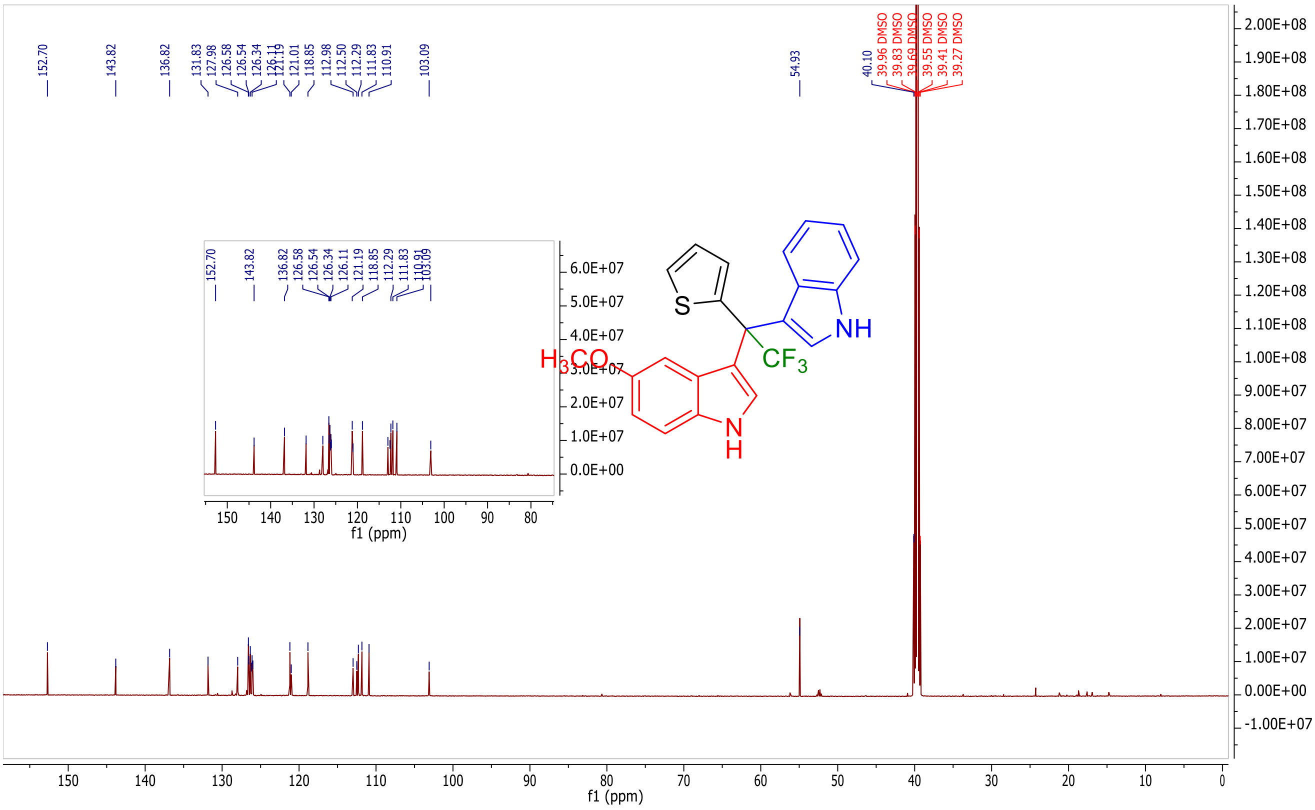

Figure S25. ^{1}H (600 MHz) and ^{13}C (151 MHz) Spectra of 5-methoxy-3-(2,2,2-trifluoro-1-(1*H*-indol-3-yl)-1-(thiophen-2-yl)ethyl)-1*H*-indole (**3v**)

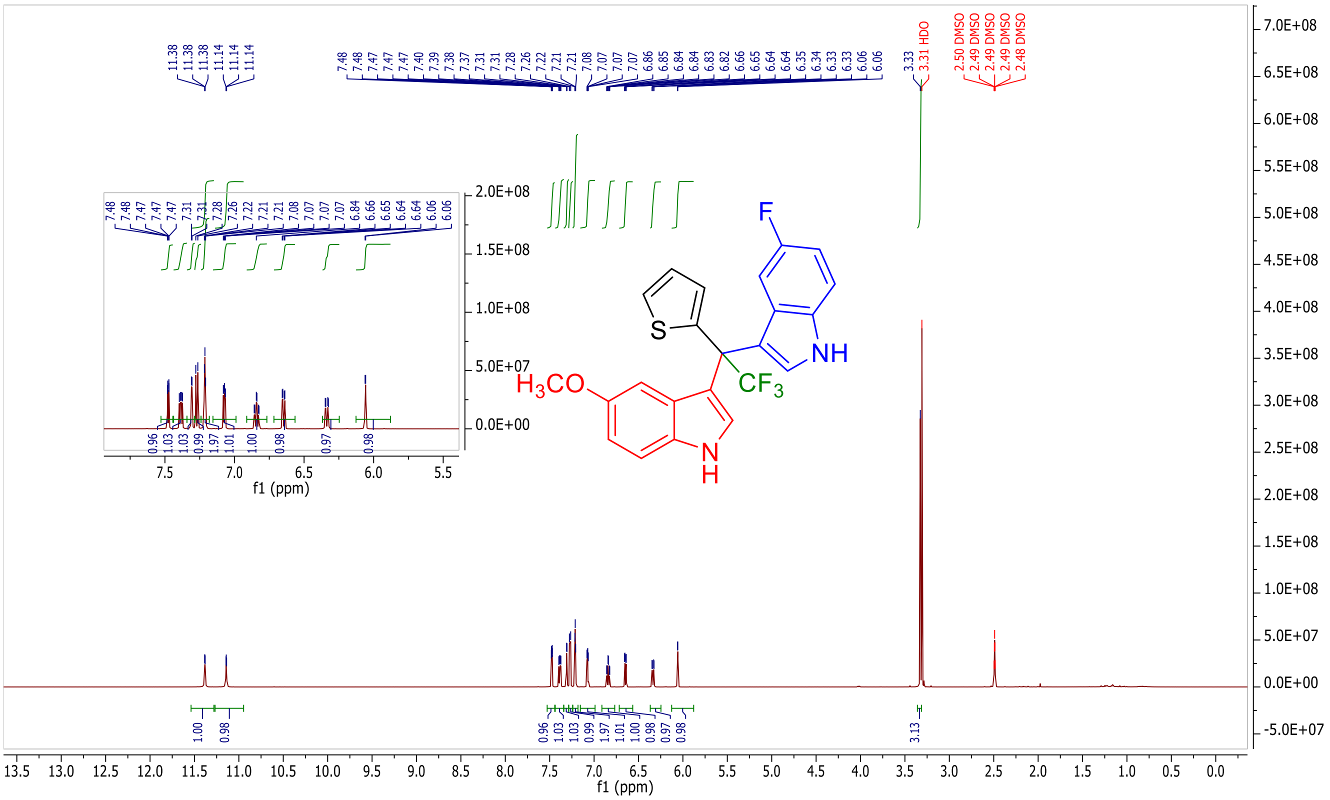

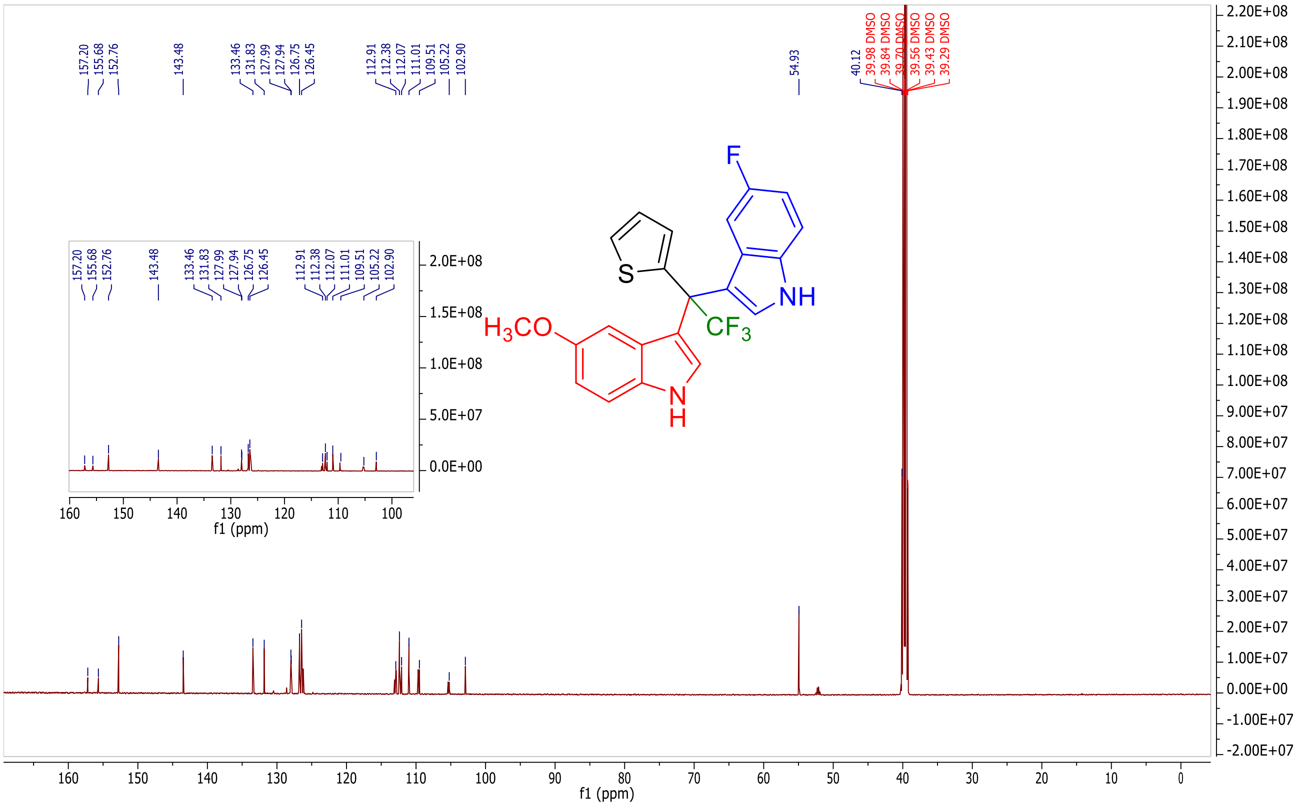

Figure S26. ^{1}H (600 MHz) and ^{13}C (151 MHz) Spectra of 5-fluoro-3-(2,2,2-trifluoro-1-(5-methoxy-1H-indol-3-yl)-1-(thiophen-2-yl)ethyl)-1H-indole (**3w**)

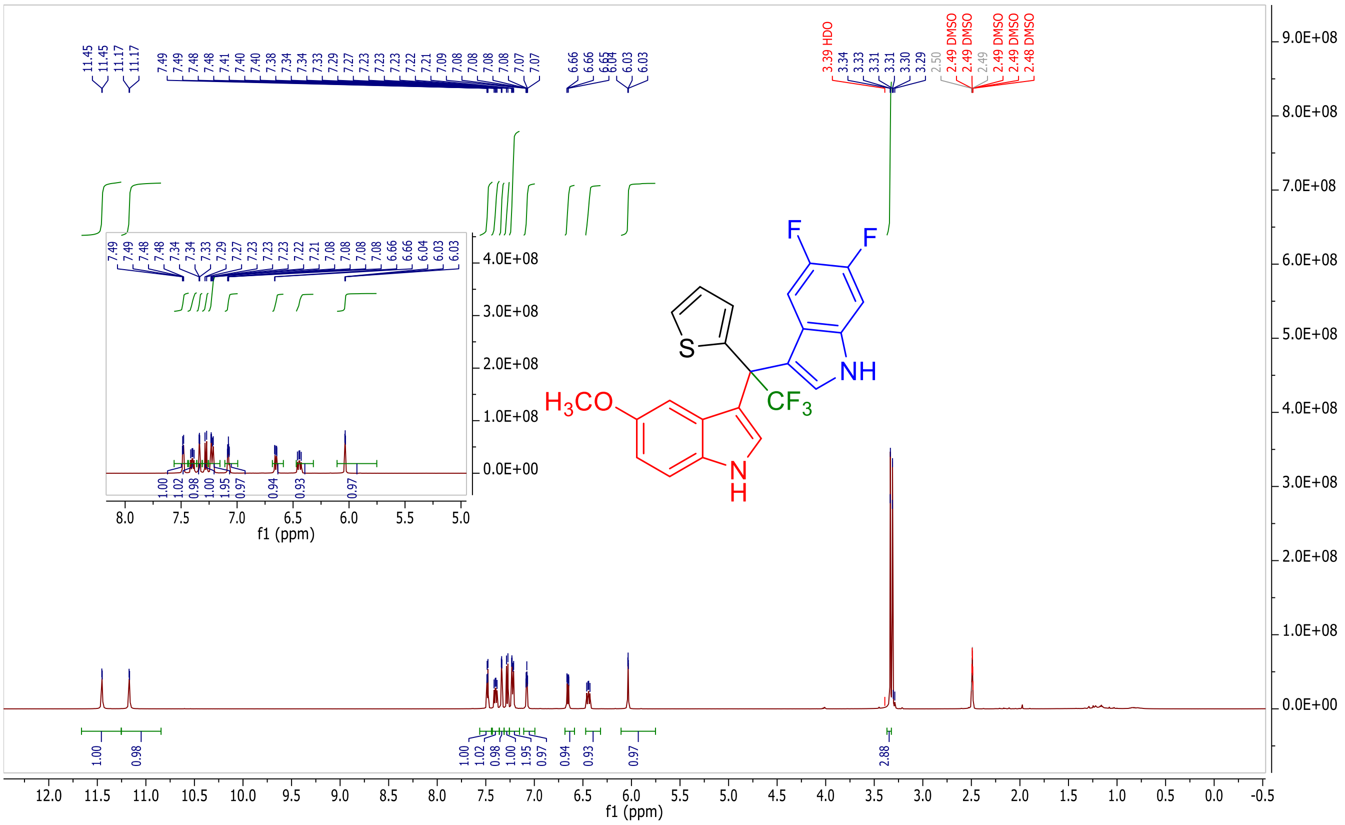

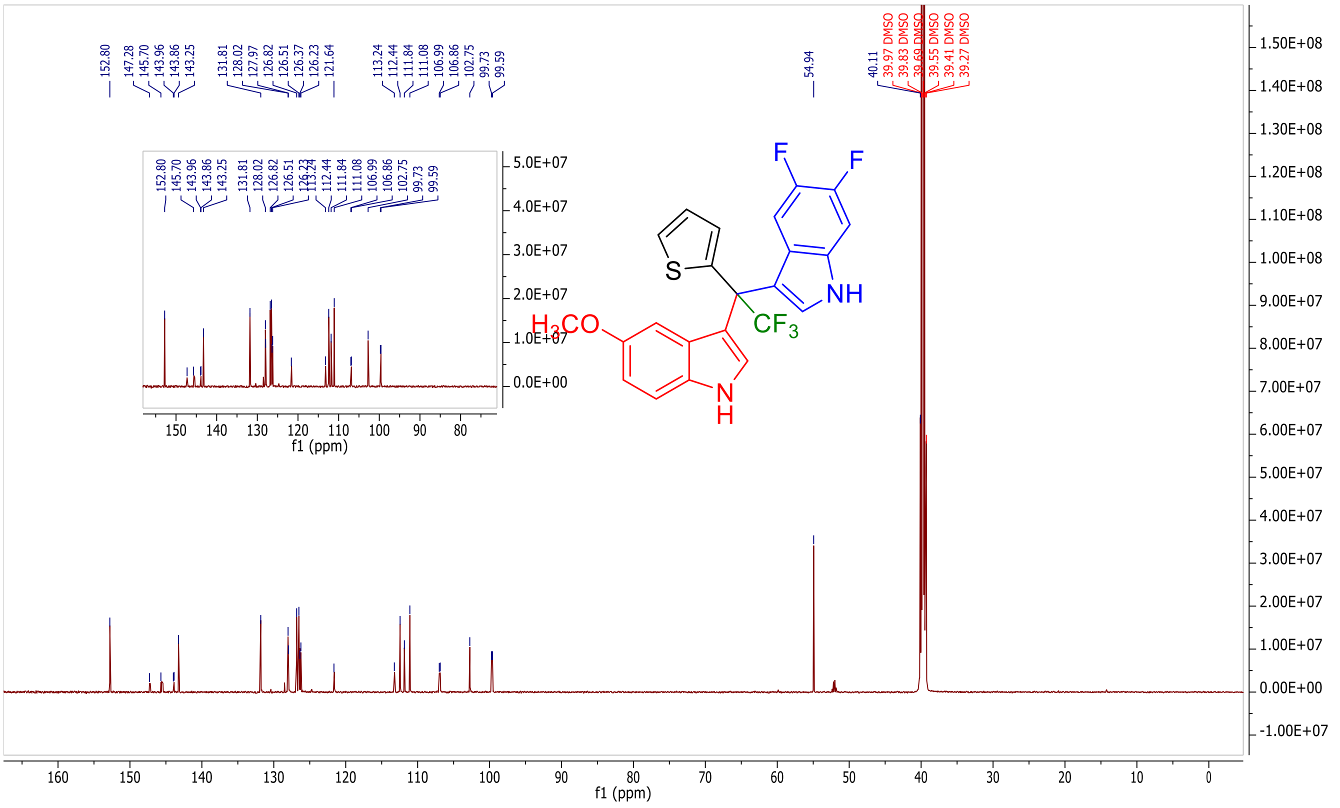

Figure S27. ^{1}H (600 MHz) and ^{13}C (151 MHz) Spectra of 5,6-difluoro-3-(2,2,2-trifluoro-1-(5-methoxy-1*H*-indol-3-yl)-1-(thiophen-2-yl)ethyl)-1*H*-indole (**3x**)

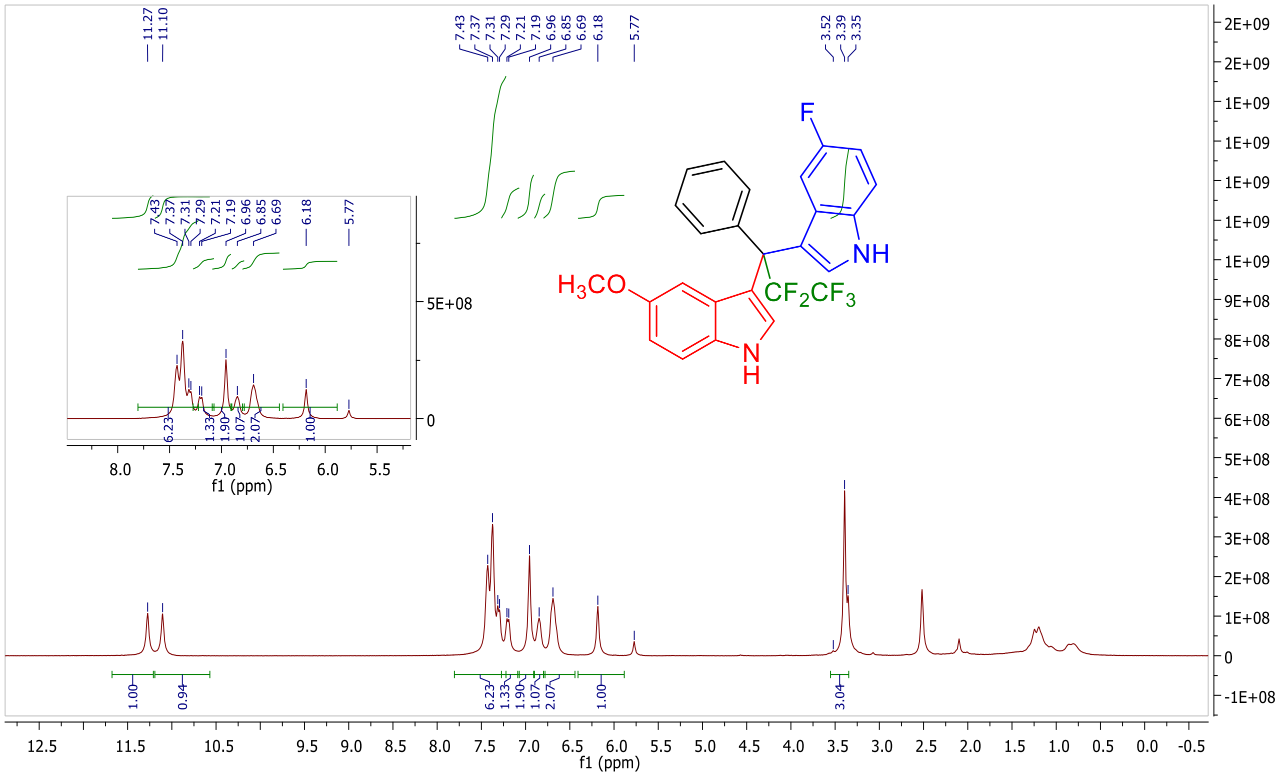

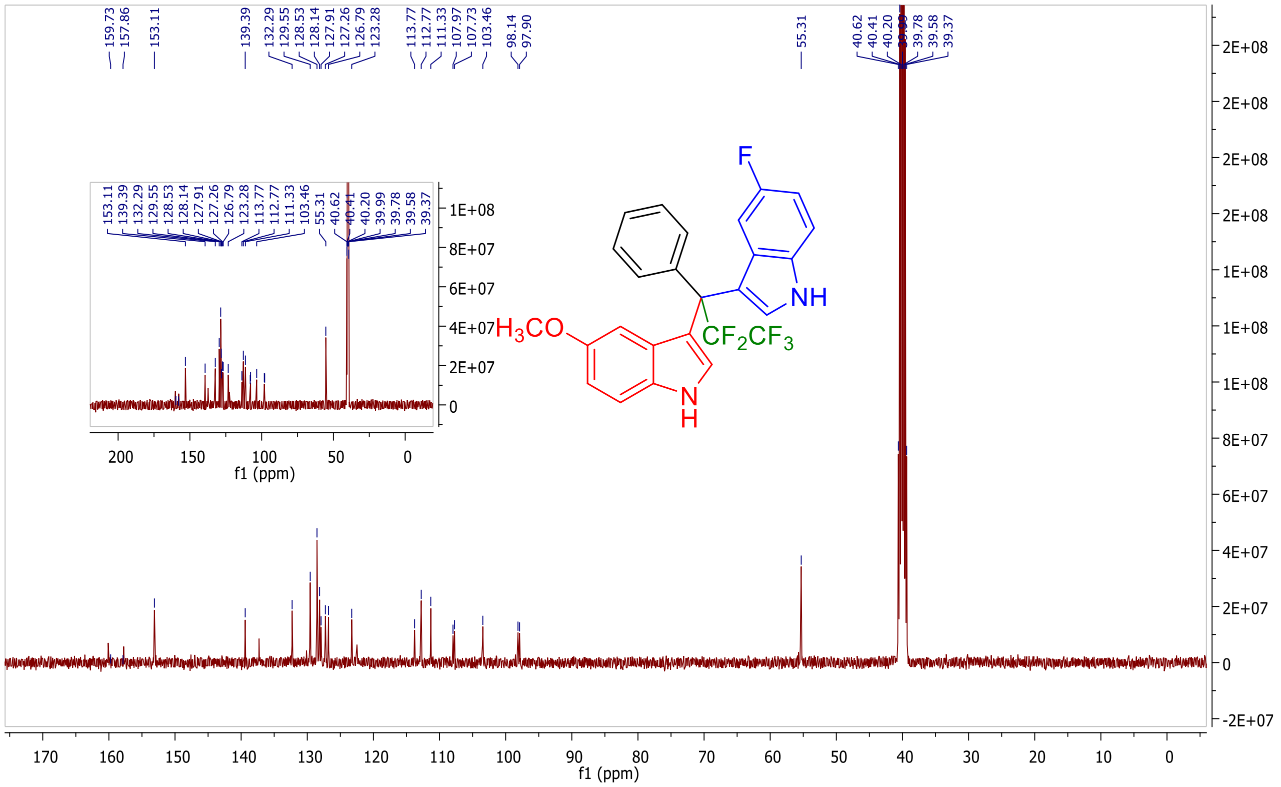

Figure S28. ^{1}H (600 MHz) and ^{13}C (151 MHz) Spectra of 5-fluoro-3-(2,2,3,3,3-pentafluoro-1-(5-methoxy-1*H*-indol-3-yl)-1-phenylpropyl)-1*H*-indole (**3y**)

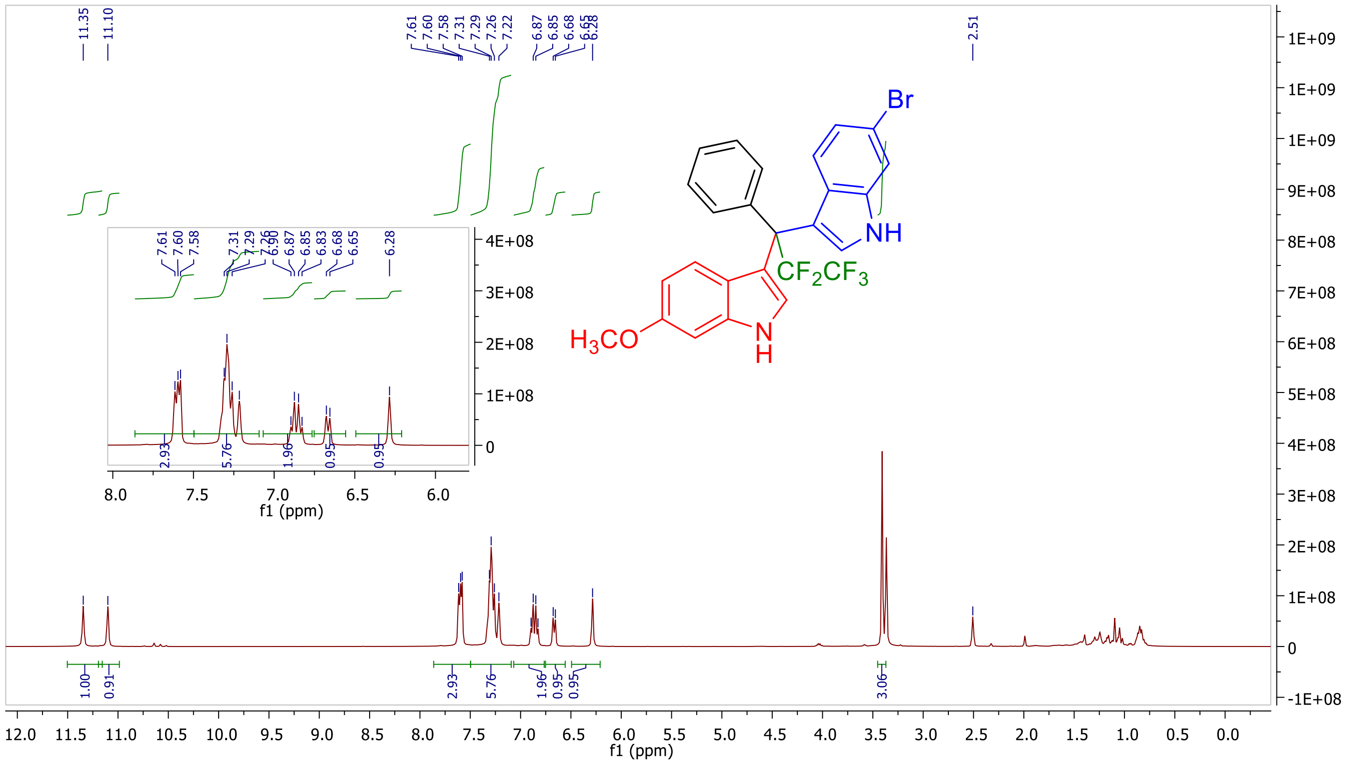

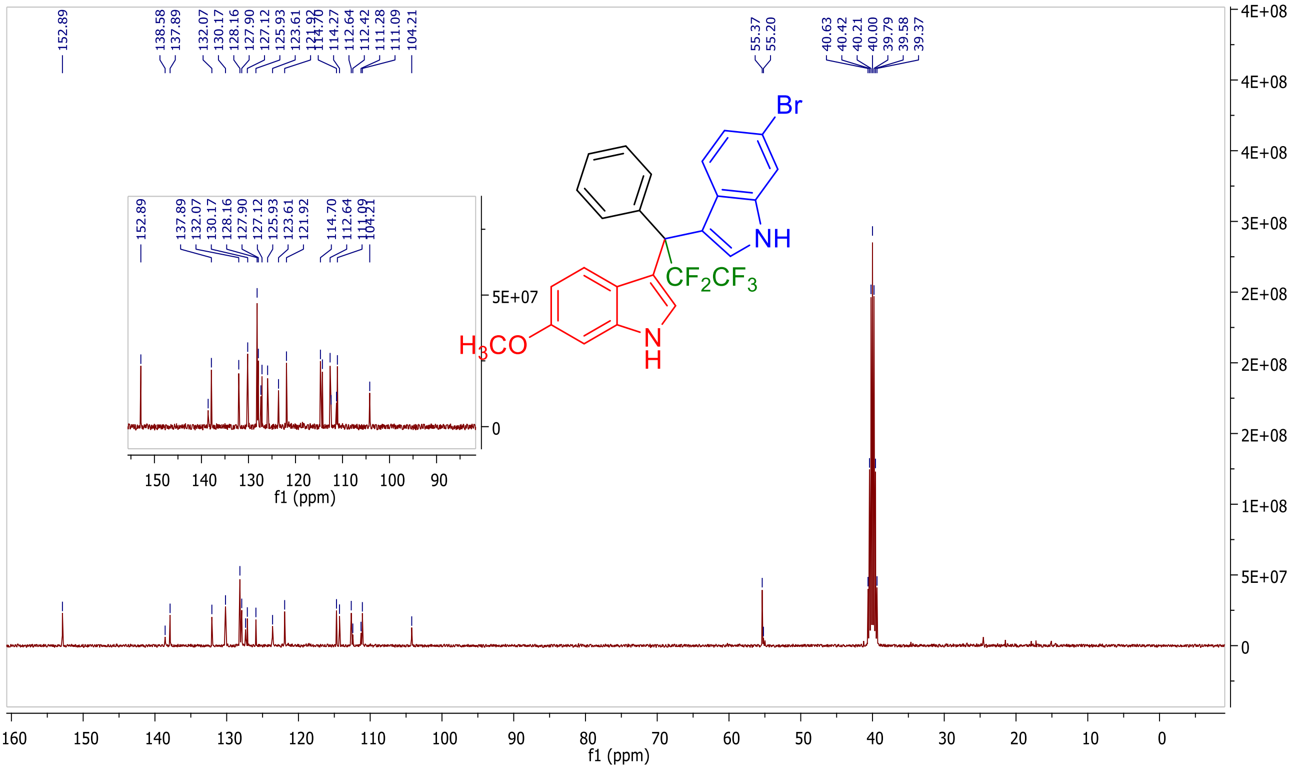

Figure S29. ^{1}H (600 MHz) and ^{13}C (151 MHz) Spectra of 6-bromo-3-(2,2,3,3,3-pentafluoro-1-(6-methoxy-1*H*-indol-3-yl)-1-phenylpropyl)-1*H*-indole (**3z**)

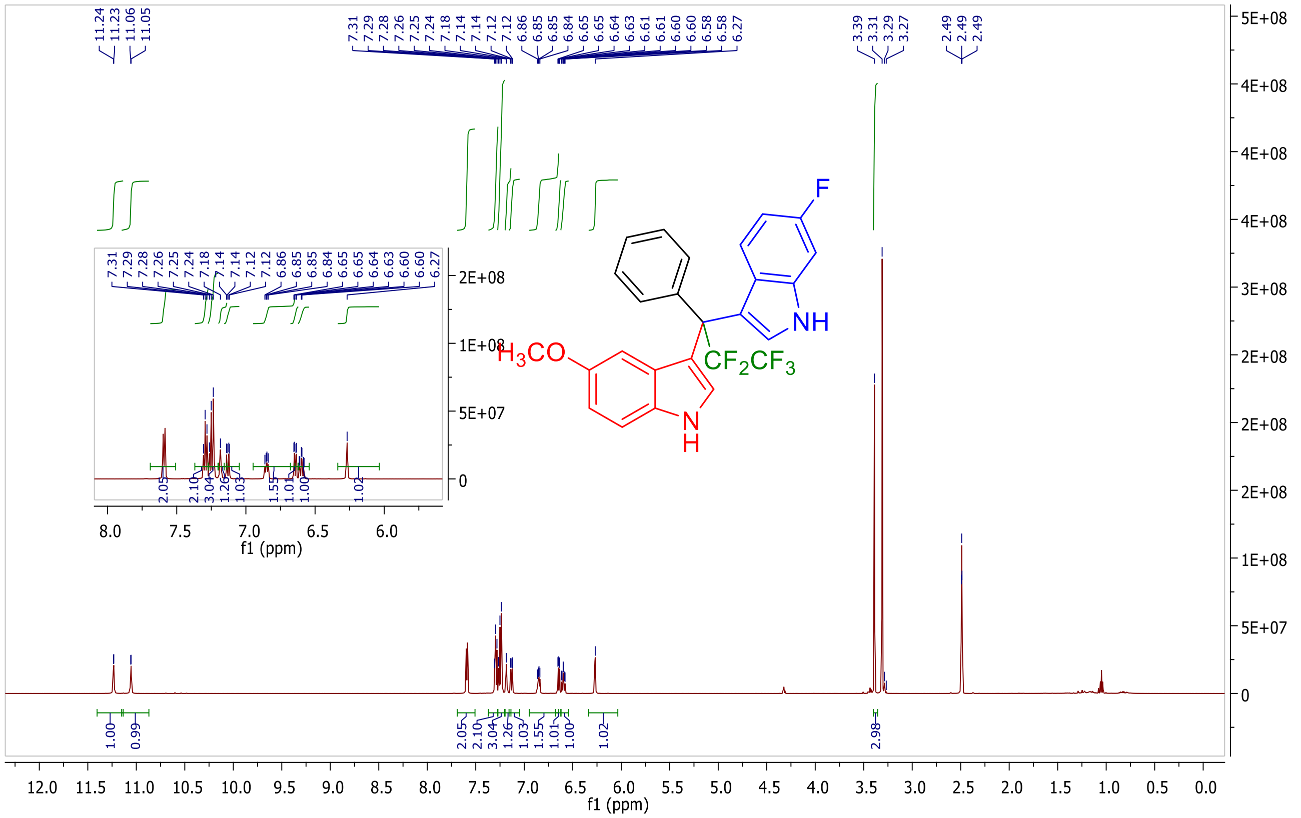

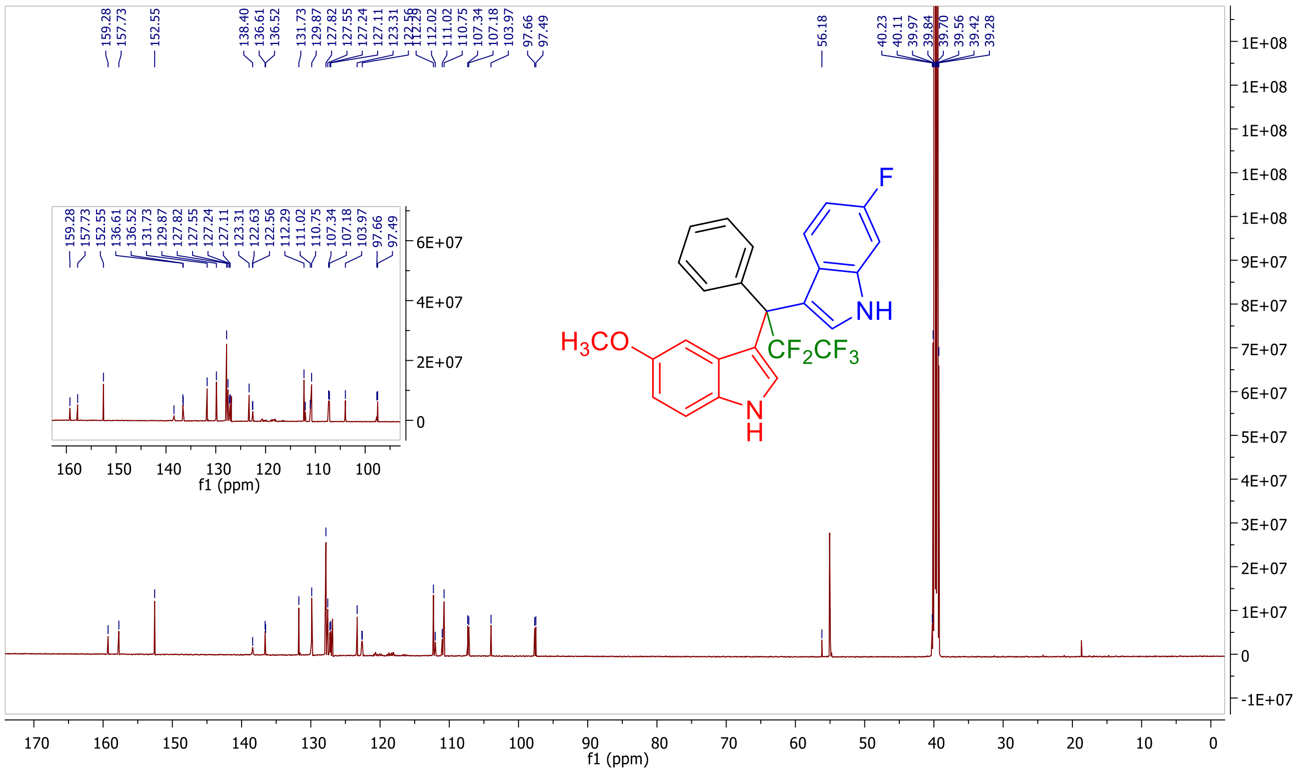

Figure S30. ^{1}H (600 MHz) and ^{13}C (151 MHz) Spectra of 6-fluoro-3-(2,2,3,3,3-pentafluoro-1-(5-methoxy-1*H*-indol-3-yl)-1-phenylpropyl)-1*H*-indole (**3aa**)

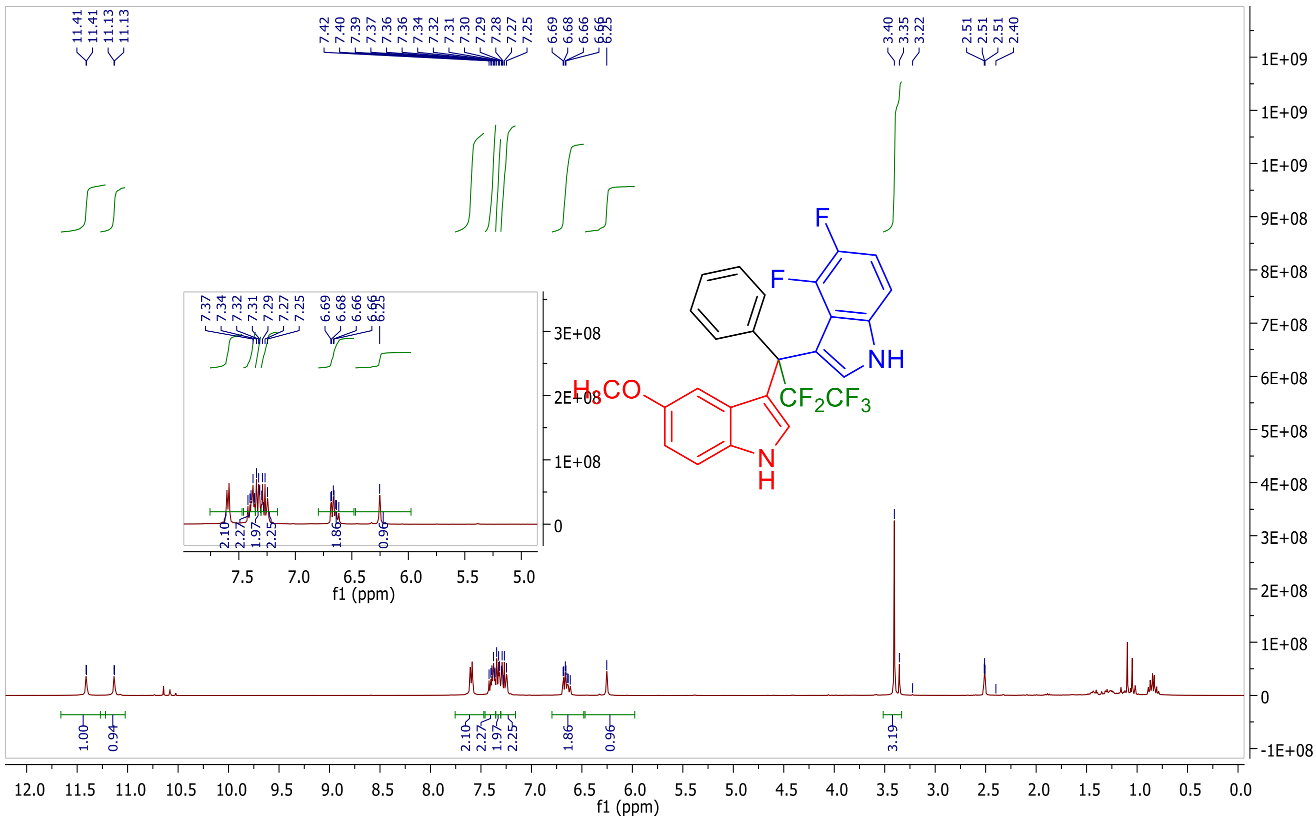

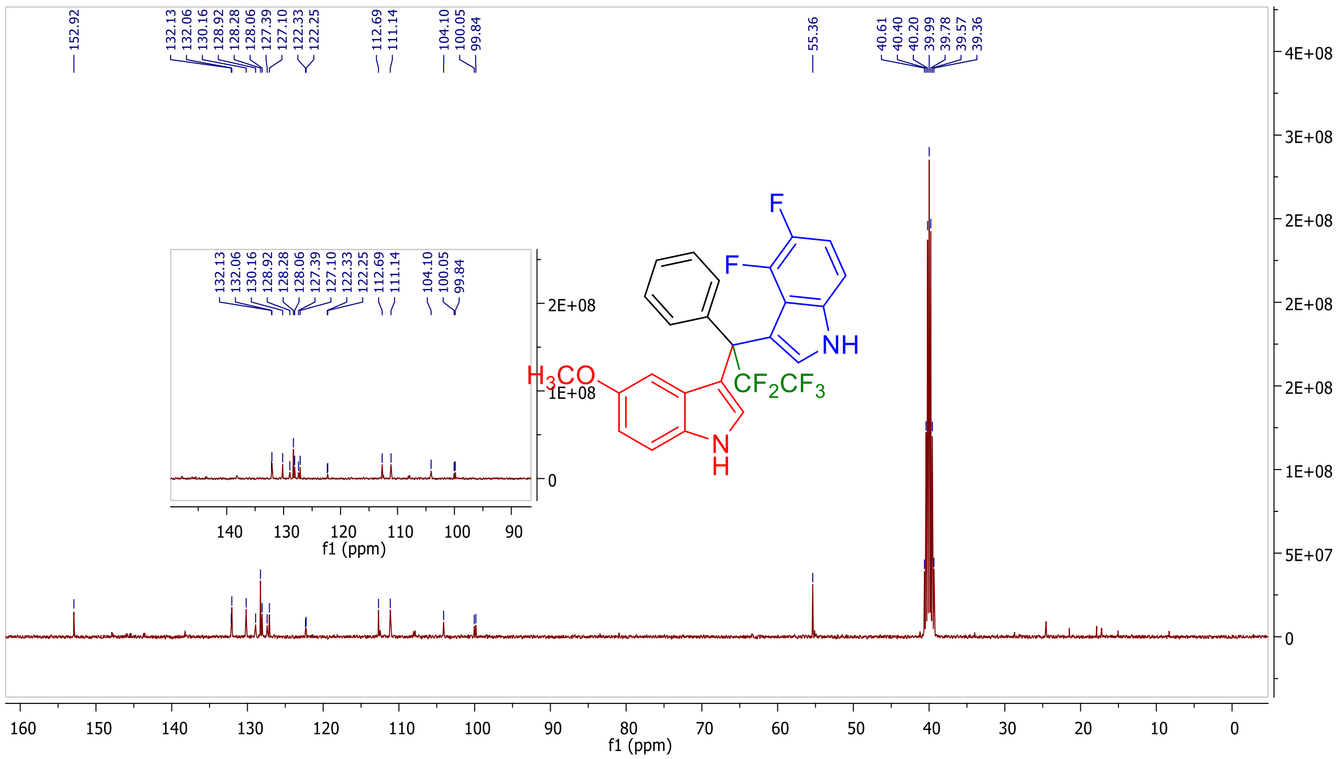

Figure S31. ^{1}H (600 MHz) and ^{13}C (151 MHz) Spectra of 4,5-difluoro-3-(2,2,3,3,3-pentafluoro-1-(5-methoxy-1*H*-indol-3-yl)-1-phenylpropyl)-1*H*-indole (**3ab**)

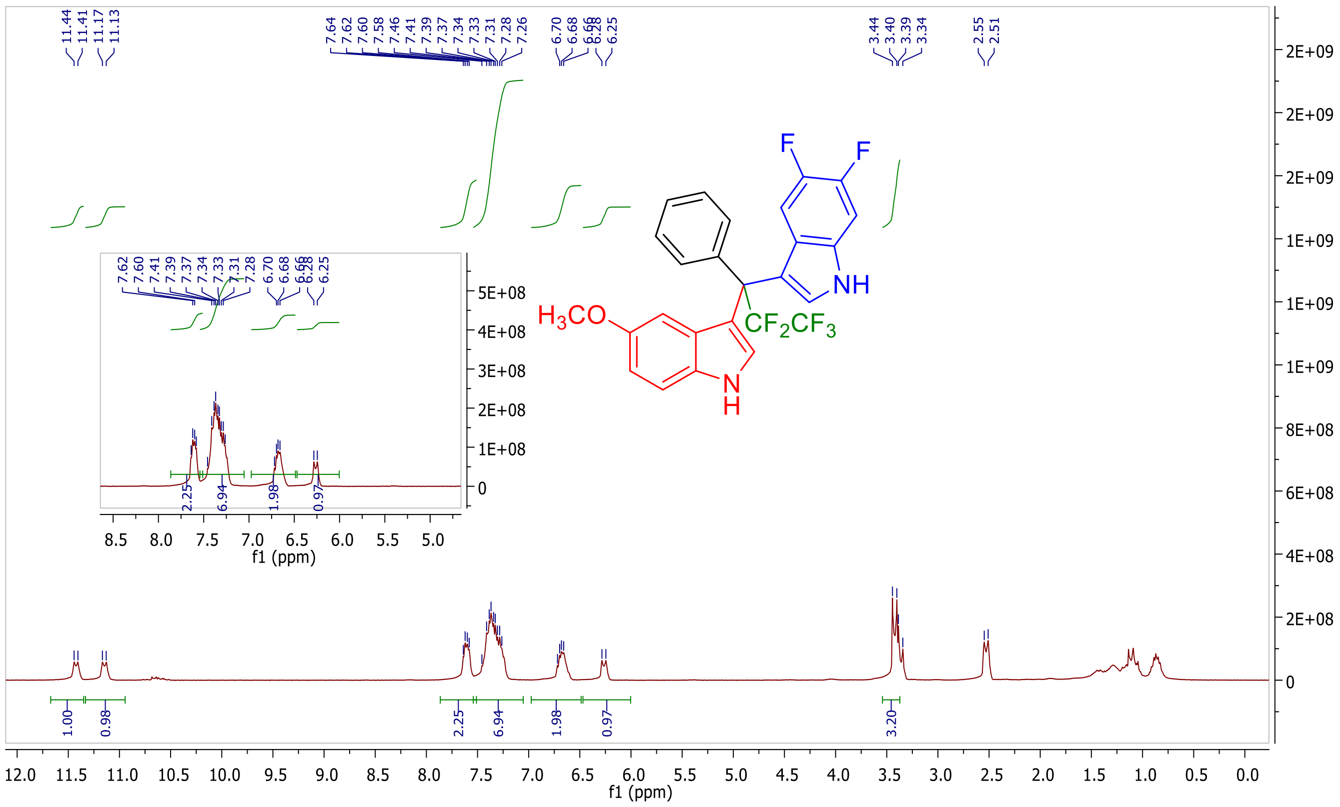

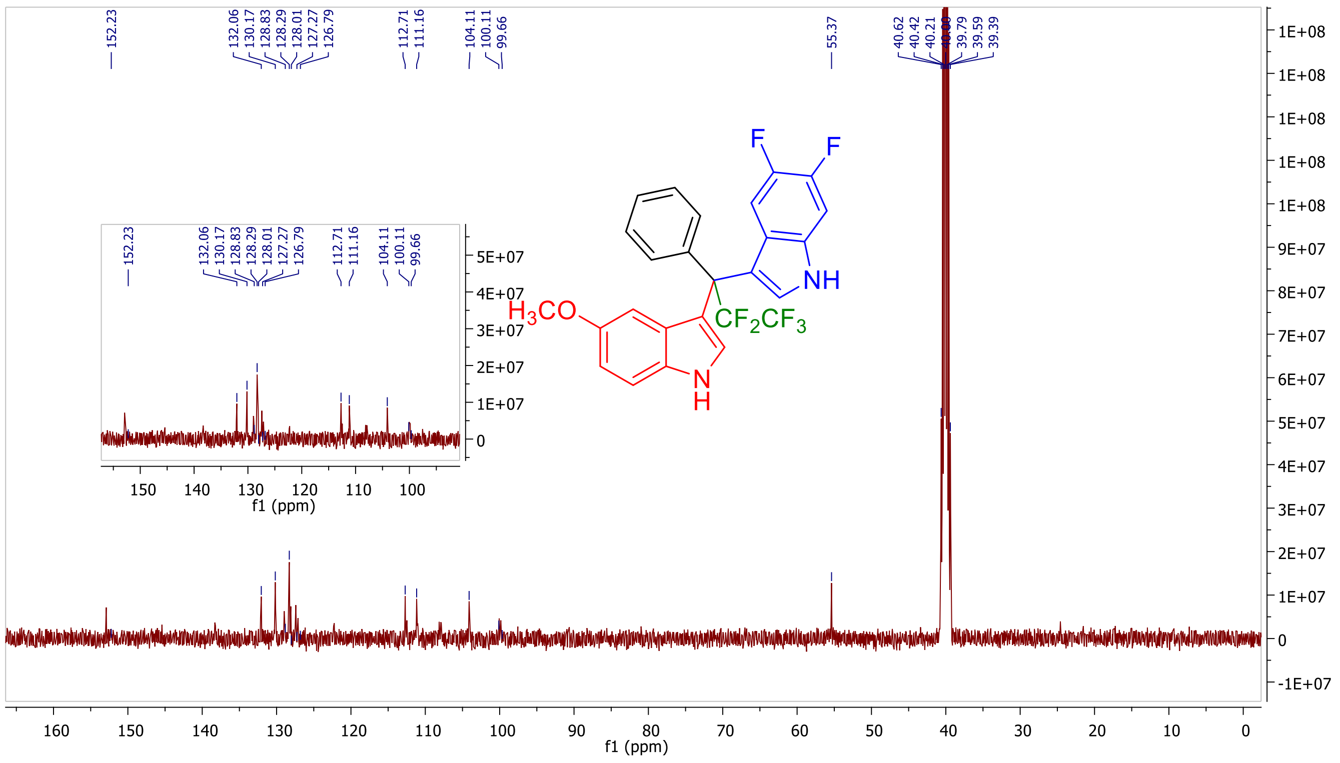

Figure S32. ^{1}H (600 MHz) and ^{13}C (151 MHz) Spectra of 5,6-difluoro-3-(2,2,3,3,3-pentafluoro-1-(5-methoxy-1*H*-indol-3-yl)-1-phenylpropyl)-1*H*-indole (**3ac)**

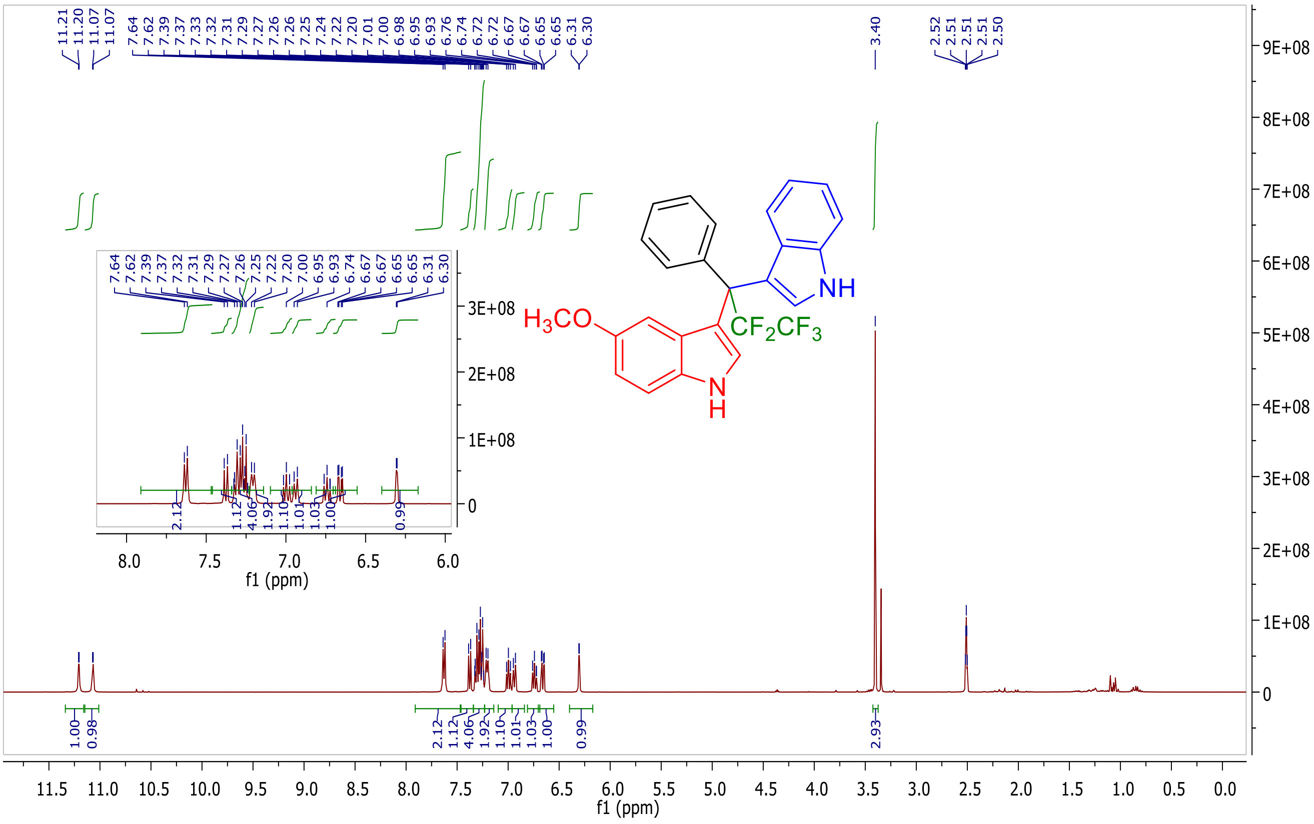

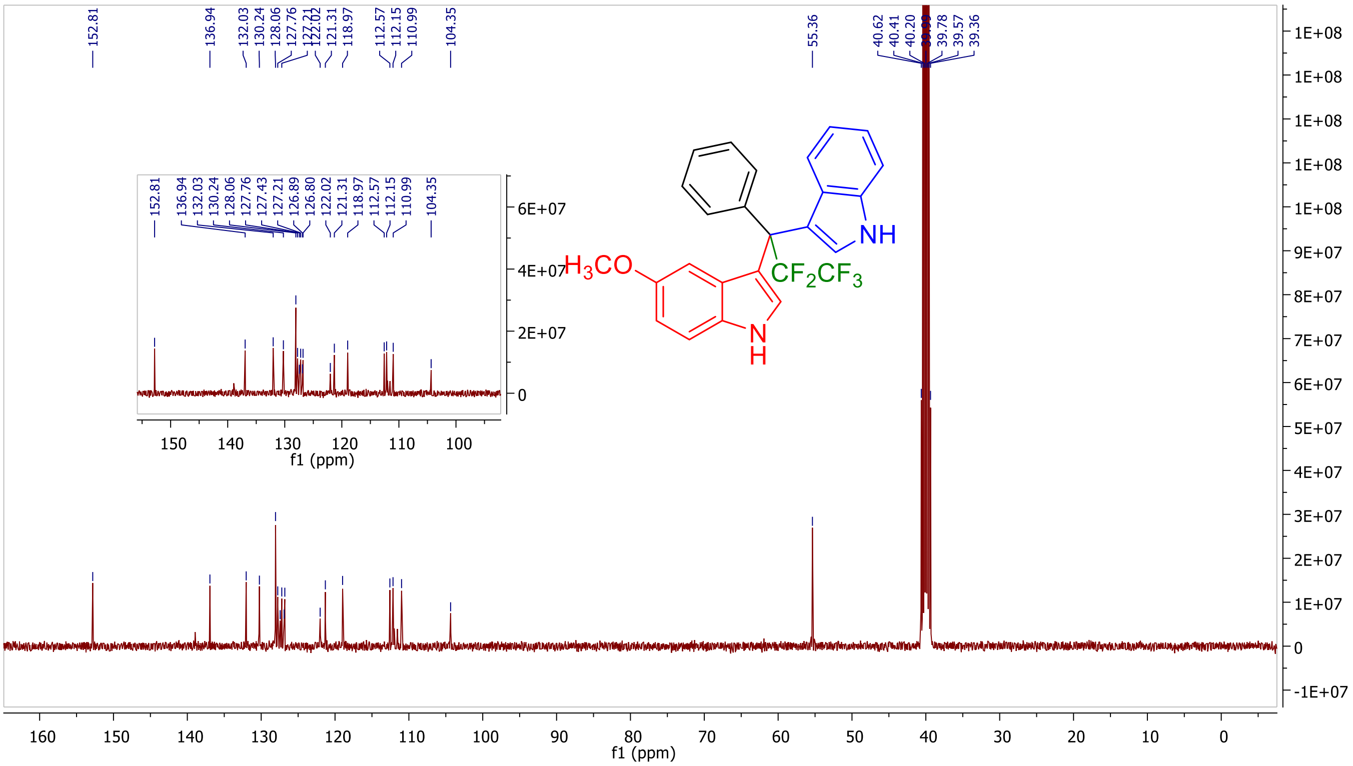

Figure S33. ^{1}H (400 MHz) and ^{13}C (151 MHz) Spectra of 5-methoxy-3-(2,2,3,3,3-pentafluoro-1-(1*H*-indol-3-yl)-1-phenylpropyl)-1*H*-indole (**3ad**)

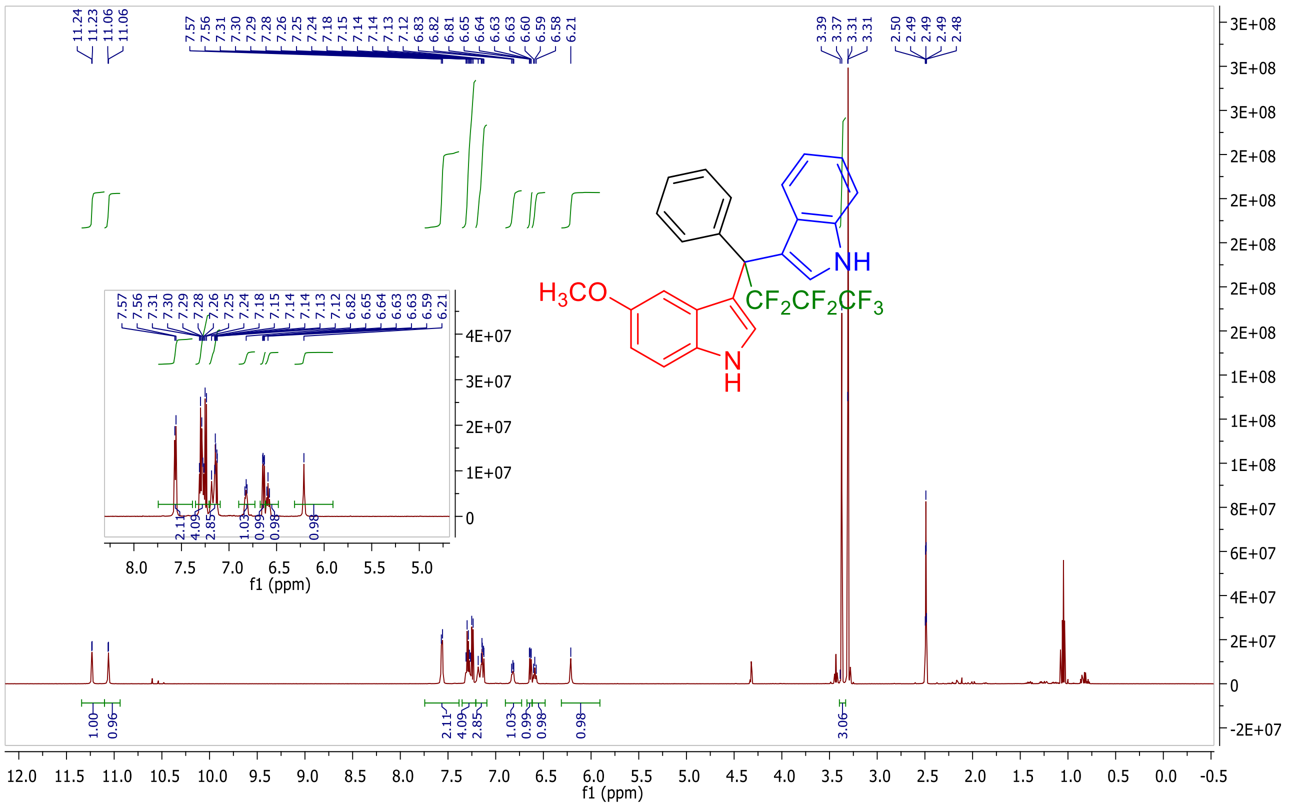

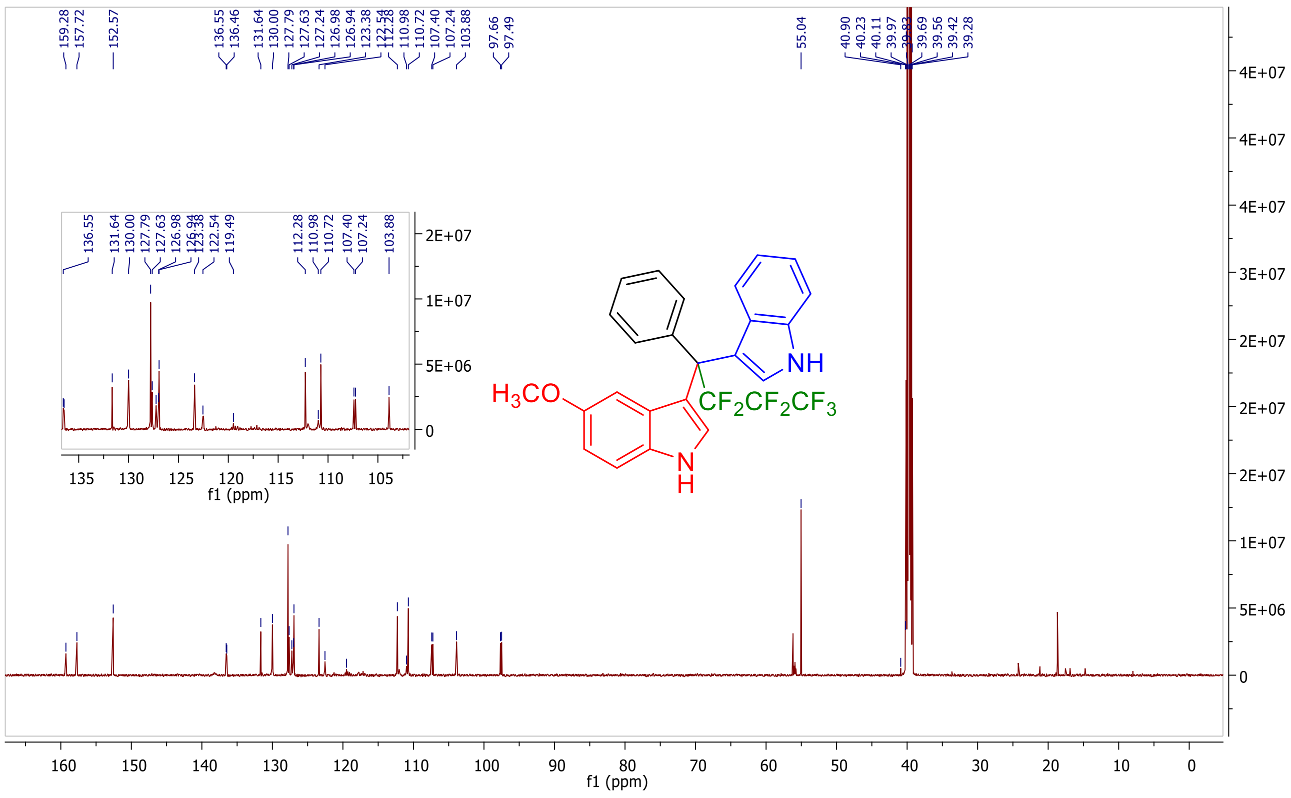

Figure S34. ^{1}H (600 MHz) and ^{13}C (151 MHz) Spectra of 3-(2,2,3,3,4,4,4-heptafluoro-1-(1*H*-indol-3-yl)-1-phenylbutyl)-5-methoxy-1*H*-indole (**3ae**)

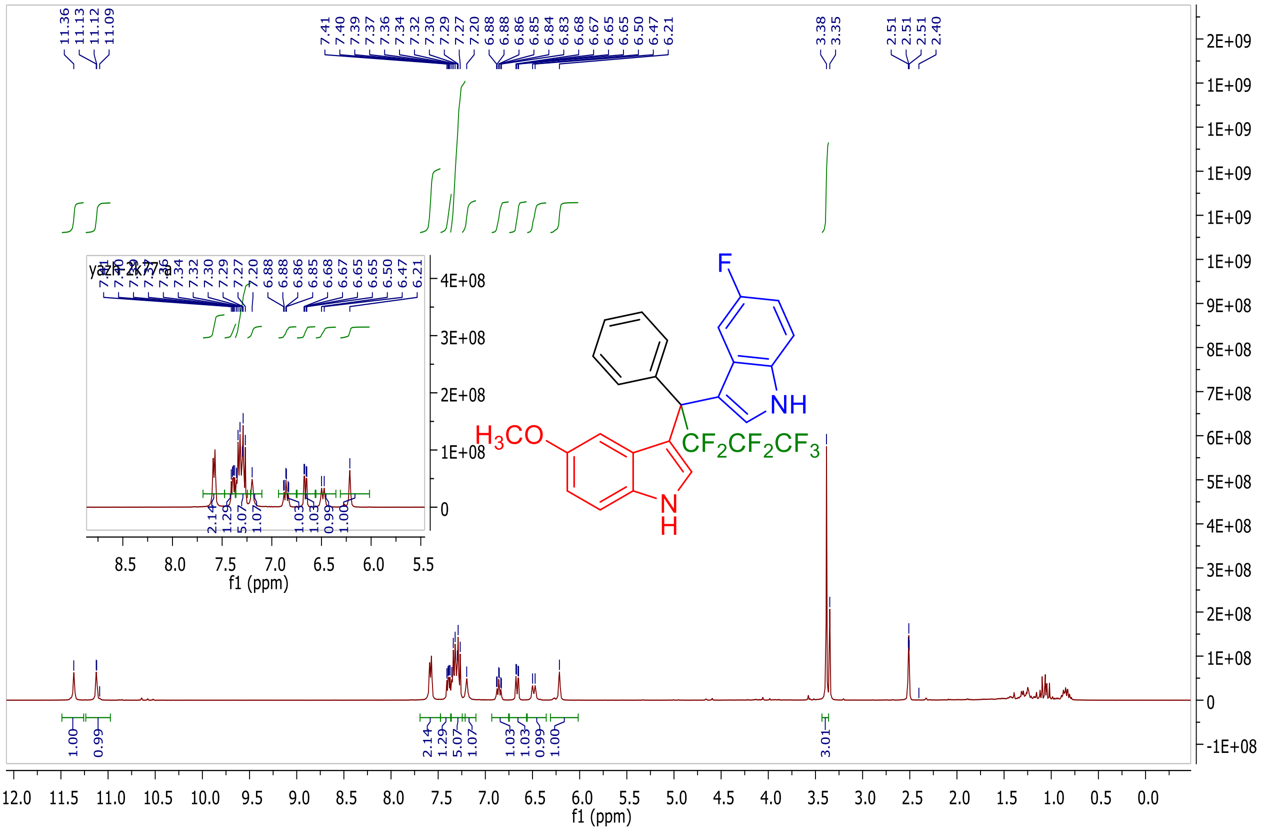

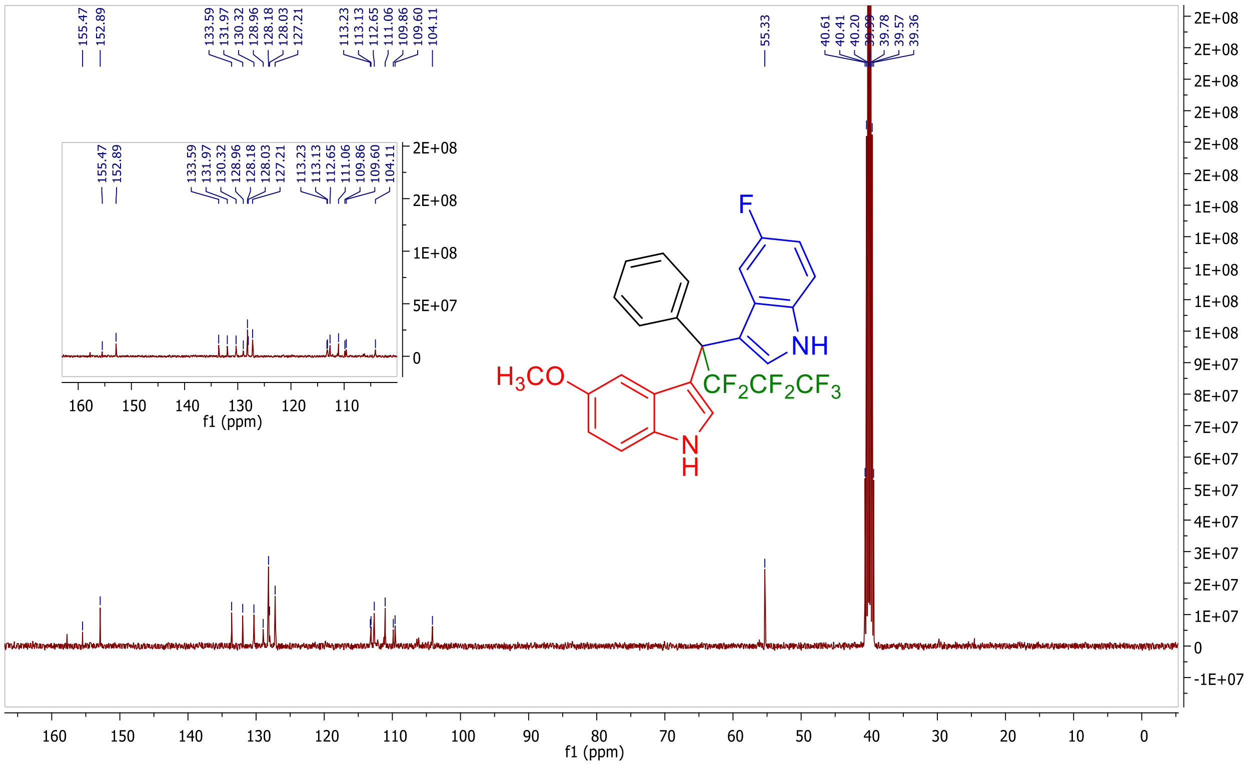

Figure S35. ^{1}H (400 MHz) and ^{13}C (151 MHz) Spectra of 5-fluoro-3-(2,2,3,3,4,4,4-heptafluoro-1-(5-methoxy-1*H*-indol-3-yl)-1-phenylbutyl)-1*H*-indole (**3af**)

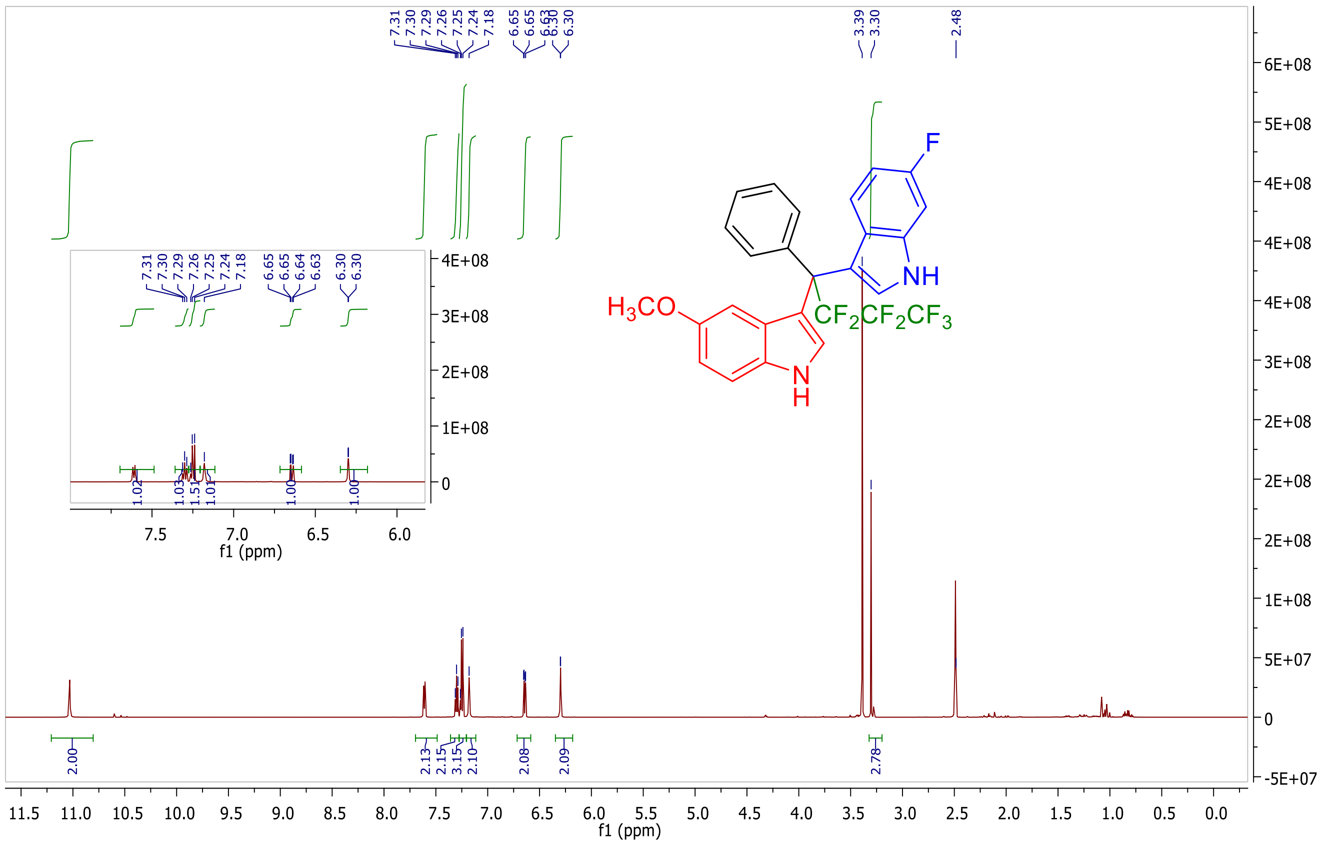

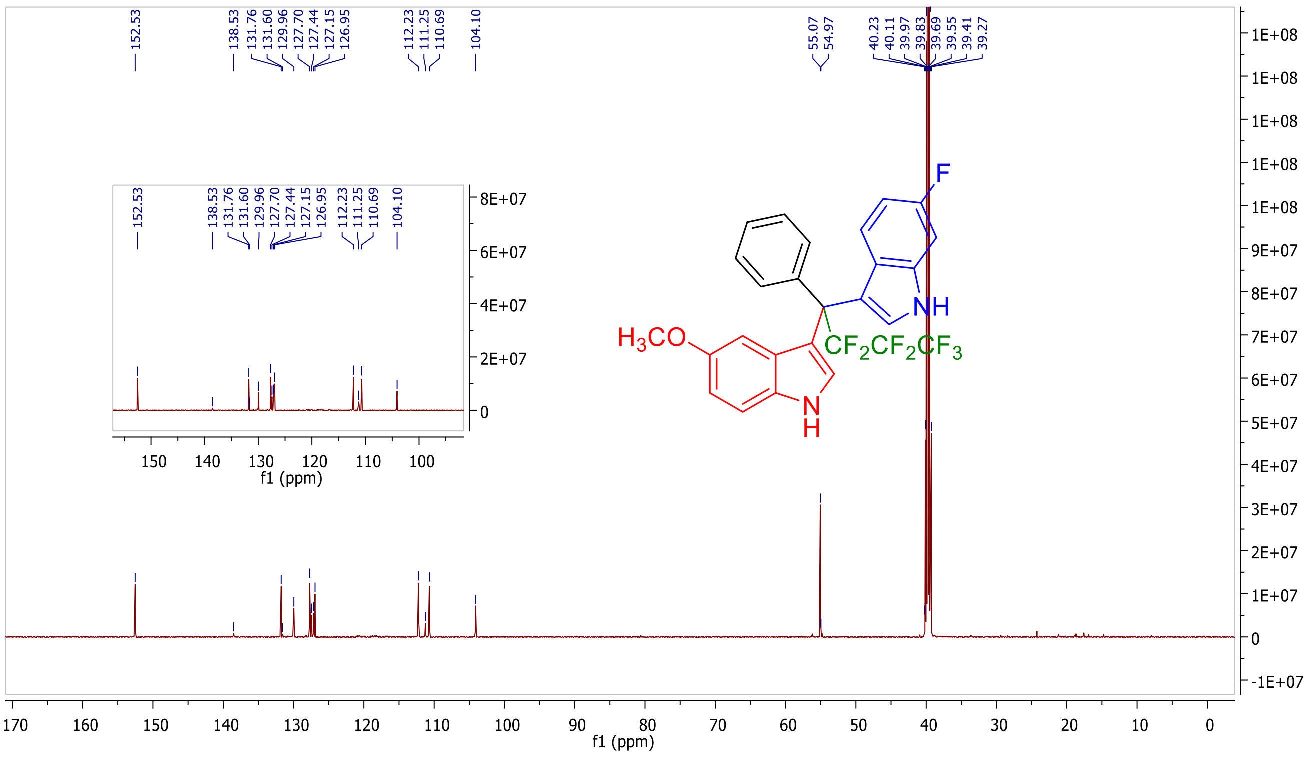

Figure S36. ^{1}H (600 MHz) and ^{13}C (151 MHz) Spectra of 6-fluoro-3-(2,2,3,3,4,4,4-heptafluoro-1-(5-methoxy-1*H*-indol-3-yl)-1-phenylbutyl)-1*H*-indole (**3ag**)

References

S1. Schoeder, C. T.; Mahardhika, A. B.; Drabczyńska, A.; Kieć-Kononowicz, K.; Müller, C. E. *ACS Med. Chem. Lett.* **2020**, 11, 2024-2031.

S2. Olsen, R. H. J.; DiBerto, J. F.; English, J. G.; Glaudin, A. M.; Krumm, B. E.; Slocum, S. T.; Che, T.; Gavin, A. C.; McCorvy, J. D.; Roth, B. L.; Strachan. R. T. *Nat. Chem. Biol.* **2020**, *16*, 841-849.

S3. Hinz, S.; Navarro, G.; Borroto-Escuela, D.; Seibt, B. F.; Ammon, Y. C.; de Filippo, E.; Danish, A.; Lacher, S. K.; Červinková, B.; Rafehi, M.; Fuxe, K.; Schiedel, A. C.; Franco, R.; Müller, C. E. *Oncotarget*, **2018**, *9*, 13593-13611.

S4. Pillaiyar, T.; Sedaghati, M.; Schnakenburg, G. *Beilstein J. Org. Chem.* **2020**, *16*, 778-790.
